# Supplementary material for: Principles and pitfalls of high-throughput analysis of microRNA-binding thermodynamics and kinetics by RNA Bind-n-Seq
Source: Cell Rep Methods. 2022 Mar 18;2(3):100185. doi: 10.1016/j.crmeth.2022.100185 (PMC9017153; doi:10.1016/j.crmeth.2022.100185)

Cell Reports Methods, Volume *2*

Supplemental information

Principles and pitfalls of high-throughput analysis of microRNA-binding thermodynamics and kinetics by RNA Bind-n-Seq

Karina Jouravleva, Joel Vega-Badillo, and Phillip D. Zamore

Table S1. Concentration of de novo identified binding sites in the RNA input pool, Related to STAR Methods

Concentrations of lsy-6, miR-1, miR-7, miR-124, miR-155, and let-7a^{hsa}, and let-7a^{mmu}, miR-34b and miR-449a sites were calculated in datasets generated in McGeary et al. (2019) and in this study, respectively.

lsy-6 sites	Concentration (nM)
8mer	0.071
7mer-m8	0.216
7mer-A1	0.141
6mer	0.571
6mer-m8	0.791
6mer-A1	0.699
5mer-m2.6	2.34
8mer-w8	0.053
8mer-b5.6A	0.015
8mer-b6.7G	0.014
8mer-x3U	0.067
8mer-x7A	0.066
10mer-m11.20	0.002

miR-1 sites	Concentration (nM)
8mer	0.069
7mer-m8	0.206
7mer-A1	0.221
6mer	0.656
6mer-m8	0.748
6mer-A1	0.637
5mer-m2.6	1.92
5mer-A1	2.83
8mer-w7	0.065
7mer-w7	0.185
8mer-b3.4U	0.019
8mer-b5.6A	0.017

miR-7 sites	Concentration (nM)
8mer	0.039
7mer-m8	0.103
7mer-A1	0.101
6mer	0.557
6mer-A1	0.506
5mer-m2.6	1.56
8mer-x7G	0.039
8mer-b6.7U	0.008
8mer-b6.7G	0.011
8mer-b7.8A	0.104

miR-124 sites	Concentration (nM)
8mer	0.042
7mer-m8	0.152
7mer-A1	0.139
6mer	0.521
6mer-m8	0.559
6mer-A1	0.594
5mer-m2.6	2.12
8mer-w5	0.038
AA-4mer-m5.8	0.606
8mer-b3.4A	0.011
8mer-b3.4C	0.011
8mer-b4.5A	0.011
8mer-b5.6U	0.012
8mer-b5.6G	0.011
8mer-b6.7A	0.011
8mer-b6.7U	0.011
A-7mer-b4.5A-w5	0.011
AA-7mer-b4.5U	0.002
AA-7mer-b5.6A	0.003
AA-7mer-b5.6U	0.002
10mer-m9.18	0.003
10mer-m10.19	0.002
10mer-m11.20	0.003
9mer-m11.19	0.009

miR-155 sites	Concentration (nM)
8mer	0.062
7mer-m8	0.169
7mer-A1	0.173
6mer	0.497
6mer-m8	0.754
8mer-w6	0.046
8mer-x5U	0.053
7mer-A1-x5U	0.015
10mer-m11.20	0.003
10mer-m12.21	0.002
10mer-m13.22	0.002
10mer-m13.22-w17	0.003
9mer-m13.21	0.009
10mer-m14.23	0.003
9mer-m14.22	0.008

let-7a^{hsa} sites	Concentration (nM)
8mer	0.071
7mer-m8	0.197
7mer-A1	0.121
6mer	0.331
6mer-m8	0.878
6mer-A1	0.716
5mer-m2.6	2.03
5mer-A1	3.16
8mer-w8	0.067
7mer-w8	0.182
8mer-b5.6A	0.018

let-7a^{mmu} sites	Concentration (nM)
8mer	0.039
7mer-m8	0.106
7mer-A1	0.049
6mer	0.136
6mer-m8	0.574
6mer-A1	0.335
5mer-m2.6	0.935
5mer-A1	2.06
8merw8	0.027
7mer-w8	0.077
8mer-w6	0.046
7mer-w6	0.127
8mer-b5.6A	0.009
10mer-m11.20	0.002
10mer-m12.21	0.001

miR-34b and miR-449a sites	Concentration (nM)
8mer	0.056
7mer-m8	0.149
7mer-A1	0.057
6mer	0.156
6mer-m8	0.645
6mer-A1	0.623
5mer-m2.6	1.78
8mer-w8	0.035
7mer-w8	0.098
8mer-w7	0.062

Table S2. RNA and DNA Oligos Used in This Study, Related to STAR Methods

RISC loading	Sequence<br>Seed; m indicates 2′-O-methyl ribose; p indicates 5′ monophosphate
Guide strand for unlabeled let-7a RISC	pUGA GGU AGU AGG UUG UAU AGU
Guide strand for Alexa let-7a RISC	pUGA GGU AGU AGG UUG UAU AGU-NH_2
Guide strand for miR-449a RISC	pUGG CAG UGU AUU GUU AGC UGG U
Guide strand for miR-34b RISC	pAGG CAG UGU AAU UAG CUG AUU GU
**RISC purification**	**Sequence**<br>RNA, DNA; m, 2′-O-methyl; p, 5′ phosphate; Bio, Biotin-6-carbon spacer
Capture Oligo to affinity purify let-7a RISC	Bio-mAmUmA mGmAmC mUmGmC mGmAmC mAmAmU mAmGmC mCmUmA mCmCmU mCmCmG mAmAmC mG
DNA competitor to elute let-7a RISC	CGT TCG GAG GTA GGC TAT TGT CGC AGT CTA T-Bio
Capture Oligo to affinity purify miR-449a RISC	Bio-mGmAmU mCmAmA mCmAmA mUmAmA mCmCmC mAmCmC mAmCmU mGmCmC mUmAmU mAmGmA
DNA competitor to elute miR-449a RISC	Bio-TTA TAG GCA GTG GTG GGT TAT TGT TGA TC
Capture Oligo to affinity purify miR-34b RISC	Bio-mGmAmU mAmUmU mCmAmA mGmCmU mAmCmC mAmCmC mAmCmU mGmCmC mUmAmU mAmA
DNA competitor to elute miR-34b RISC	Bio-TTA TAG GCA GTG GTG GTA GCT TGA ATA TC
**RISC quantification**	**Sequence**<br>RNA, DNA; p, 5′ phosphate
DNA probe to quantify total concentration of let-7a RISC by Northern Blot	GAT ACT ATA CAA CCT ACT ACC TCA ACC T
DNA probe to quantify total concentration of miR-449a RISC by Northern Blot	ATG ACC AGC TAA CAA TAC ACT GCC AAC T
DNA probe to quantify total concentration of miR-34b RISC by Northern Blot	ATG ACA ATC AGC TAA TTA CAC TGC CTA CT

RNA target to quantify active concentration of let-7a RISC by double-filter binding	pAAA AUG AUA ACA AGG AUC UAC CUC AAA A
RNA target to quantify active concentration of miR-449a and miR-34b RISCs by double-filter binding	pAUG AAA UCG AUA UCU AUC ACU GCC AAC U
**CoSMoS**	**Sequence**<br>RNA, DNA; Bio, Biotin-6-carbon spacer; target region; U, Alexa Fluor 647 deoxyuridine
Klenow polymerase template to synthesize 3′ DNA extension containing 17 Alexa Fluor 647 dyes for let-7a RNA with t10–t19 site adjacent to t3–t6 site	ATT GTT GTT ATT GTT GTT ATT GTT GTT ATT GTT GTT ATT GTT GTT ATT GTT<br>GTT ATT GTT GTT ATT GTT GTT ATT GTT GTT ATT GTT GTT ATT GTT GTT ATT<br>GTT GTT ATT GTT GTT ATT GTT GTT ATT GTT GTT ATT GTT GTT ATT TAC ATC<br>TAG GTC TTA GTC
Trap oligonucleotide for the preceding template (fully complementary)	GAC TAA GAC CTA GAT GTA AAT AAC AAC AAT AAC AAC AAT AAC AAC AAT AAC<br>AAC AAT AAC AAC AAT AAC AAC AAT AAC AAC AAT AAC AAC AAT AAC AAC AAT<br>AAC AAC AAT AAC AAC AAT AAC AAC AAT AAC AAC AAT AAC AAC AAT AAC AAC<br>AAT AAC AAC AAT
5′-tethered, RNA target with let-7a t10–t19 site adjacent to t3–t6 site and a 3′ DNA extension containing 17 Alexa Fluor 647 dyes	Bio-AUU AAA UAU ACA ACC UGA CUA AGA CCU AGA UGU AAA UAA CAA CAA UAA<br>CAA CAA UAA CAA CAA UAA CAA CAA UAA CAA CAA UAA CAA CAA UAA CAA CAA<br>UAA CAA CAA UAA CAA CAA UAA CAA CAA UAA CAA CAA UAA CAA CAA UAA CAA<br>CAA UAA CAA CAA UAA CAA CAA UAA CAA CAA U
Klenow polymerase template to synthesize 3′ DNA extension containing 17 Alexa Fluor 647 dyes for let-7a RNA with 6mer site	ATT GTT GTT ATT GTT GTT ATT GTT GTT ATT GTT GTT ATT GTT GTT ATT GTT<br>GTT ATT GTT GTT ATT GTT GTT ATT GTT GTT ATT GTT GTT ATT GTT GTT ATT<br>GTT GTT ATT GTT GTT ATT GTT GTT ATT GTT GTT ATT GTT GTT ATT TAC ATC<br>TAG AGA GGT ATA
Trap oligonucleotide for the preceding template (fully complementary)	TAT ACC TCT CTA GAT GTA AAT AAC AAC AAT AAC AAC AAT AAC AAC AAT AAC<br>AAC AAT AAC AAC AAT AAC AAC AAT AAC AAC AAT AAC AAC AAT AAC AAC AAT<br>AAC AAC AAT AAC AAC AAT AAC AAC AAT AAC AAC AAT AAC AAC AAT AAC AAC<br>AAT AAC AAC AAT
5′-tethered, RNA target with let-7a 6mer site and a 3′ DNA extension containing 17 Alexa Fluor 647 dyes	Bio-AUU AAA UAA UGG AGA AUU AAA UAU ACC UCU CUA GAU GUA AAU AAC AAC<br>AAU AAC AAC AAU AAC AAC AAU AAC AAC AAU AAC AAC AAU AAC AAC AAU AAC<br>AAC AAU AAC AAC AAU AAC AAC AAU AAC AAC AAU AAC AAC AAU AAC AAC AAU<br>AAC AAC AAU AAC AAC AAU AAC AAC AAU AAC AAC AAU
Klenow polymerase template to synthesize 3′ DNA extension containing 17 Alexa Fluor 647 dyes for other let-7a RNA targets	ATT GTT GTT ATT GTT GTT ATT GTT GTT ATT GTT GTT ATT GTT GTT ATT GTT<br>GTT ATT GTT GTT ATT GTT GTT ATT GTT GTT ATT GTT GTT ATT GTT GTT ATT<br>GTT GTT ATT GTT GTT ATT GTT GTT ATT GTT GTT ATT GTT GTT ATT TAC ATC<br>TTC GTC TTA GTC

Trap oligonucleotide for the preceding template (fully complementary)	GAC TAA GAC GAA GAT GTA AAT AAC AAC AAT AAC AAC AAT AAC AAC AAT AAC AAC AAT AAC AAC AAT AAC AAC AAT AAC AAC AAT AAC AAC AAT AAC AAC AAT AAC AAC AAT AAC AAC AAT AAC AAC AAT AAC AAC AAT AAC AAC AAT AAC AAC AAT AAC AAC AAT
5′-tethered, RNA target with let-7a t10–t19 site and a 3′ DNA extension containing 17 Alexa Fluor 647 dyes	Bio-AUU AAA UAU ACA ACC UGA CUA AGA CGA AGA UGU AAA UAA CAA CAA UAA CAA CAA UAA CAA CAA UAA CAA CAA UAA CAA CAA UAA CAA CAA UAA CAA CAA UAA CAA CAA UAA CAA CAA UAA CAA CAA UAA CAA CAA UAA CAA CAA UAA CAA CAA UAA CAA CAA UAA CAA CAA UAA CAA CAA U
5′-tethered, RNA target with let-7a 6mer-A1 site and a 3′ DNA extension containing 17 Alexa Fluor 647 dyes	Bio-AUC UAA AUA AAC CUC AGA CUA AGA CGA AGA UGU AAA UAA CAA CAA UAA CAA CAA UAA CAA CAA UAA CAA CAA UAA CAA CAA UAA CAA CAA UAA CAA CAA UAA CAA CAA UAA CAA CAA UAA CAA CAA UAA CAA CAA UAA CAA CAA UAA CAA CAA UAA CAA CAA UAA CAA CAA UAA CAA CAA U
5′-tethered, RNA target with let-7a 6mer-m8 site and a 3′ DNA extension containing 17 Alexa Fluor 647 dyes	Bio-AUU UCC AAG CCU ACC UGA CUA AGA CGA AGA UGU AAA UAA CAA CAA UAA CAA CAA UAA CAA CAA UAA CAA CAA UAA CAA CAA UAA CAA CAA UAA CAA CAA UAA CAA CAA UAA CAA CAA UAA CAA CAA UAA CAA CAA UAA CAA CAA UAA CAA CAA UAA CAA CAA UAA CAA CAA UAA CAA CAA U
**RBNS**	**Sequence**<br>RNA, DNA
RBNS RNA pool	GAG UUC UAC AGU CCG ACG AUC NNN NNN NNN NNN NNN NNN NNU GGA AUU CUC GGG UGC CAA
RT primer	CCT TGG CAC CCG AGA ATT CCA
PCR Forward primer	AAT GAT ACG GCG ACC ACC GAG ATC TAC ACG TTC AGA GTT CTA CAG TCC GAC GAT C
Multiplexing PCR Reverse Primer (XXXXXX represents barcode)	CAA GCA GAA GAC GGC ATA CGA GAT XXX XXX GTG ACT GGA GTT CCT TGG CAC CCG AGA ATT CCA

Table S3. Dissociation constants for 3′-only sites, Related to Figure 6

Values are reported in pM. Intervals correspond to 95% confidence intervals on the median.

miRNA	6mer	10mer-m10.19	10mer-m11.20	10mer-m12.21	10mer-m13.22	10mer-m14.23
let-7a^{mmu}	348 [337,387]	630 [469, 830]	158 [145, 165]	323 [297, 335]	NA	NA
let-7a^{hsa}	82.5 [81.7, 83.6]	689 [677, 698]	512 [507, 519]	1414 [1379, 1475]	2733 [2699, 2771]	NA
miR-124	475 [472, 478]	110 [89, 141]	226 [221, 233]	635 [624, 643]	1390 [1381, 1401]	NA
miR-155	154 [152, 155]	591 [586, 597]	250 [248, 252]	94.2 [92.7, 95.2]	36.7 [36.3, 37.0]	139 [138, 141]
miR-1	62.1 [62.0, 62.2]	2441 [2402, 2480]	1199 [1181, 1218]	1298 [1281, 1327]	2334 [2282, 2395]	NA
lsy-6	450 [447, 451]	241 [239, 243]	386 [380, 393]	776 [763, 784]	710 [704, 717]	NA
miR-7	140 [140, 140]	2128 [2090, 2175]	1420 [1394, 1439]	1397 [1373, 1414]	1745 [1711, 1771]	2301 [2262, 2338]
miR-34b	167 [720, 733]	3211 [3160, 3271]	726 [720, 733]	998 [986, 1010]	1058 [1049, 1067]	1712 [1702, 1726]
miR-449a	62.4 [57.5, 62.8]	448 [443, 451]	869 [852, 889]	679 [670, 687]	936 [921, 962]	NA

Table S4. Robustness of fitted k_{on} and k_{off} parameters, Related to Figure 7

Maximum likelihood estimation was performed on sub-datasets where each sample was removed one by one. Pearson's *r* was calculated for each of the fifty-five pairwise combinations.

	**0 s**	**15 s**	**30 s**	**1 min**	**2.5 min**	**5 min**	**10 min**	**15 min**	**30 min**	**1 h**
**15 s**	1.00									
**30 s**	0.993	0.993								
**1 min**	0.999	0.999	0.995							
**2.5 min**	1.00	1.00	0.993	0.999						
**5 min**	1.00	1.00	0.993	0.999	1.00					
**10 min**	1.00	1.00	0.994	0.999	1.00	1.00				
**15 min**	1.00	1.00	0.993	0.999	1.00	1.00	1.00			
**30 min**	1.00	1.00	0.993	0.999	1.00	1.00	1.00	1.00		
**1 h**	1.00	1.00	0.993	0.999	1.00	1.00	1.00	1.00	1.00	
**2 h**	1.00	1.00	0.993	0.999	1.00	1.00	1.00	1.00	1.00	1.00

SUPPLEMENTAL FIGURES AND LEGENDS

Figure S1. RBNS biases relative to RNA pool design, Related to Figure 1

(A) Frequency of the four nucleotides at each position within the randomized region of RNA molecules. Adenine (orange), uridine (magenta), cytidine (cyan), and guanosine (blue).

(**B**) Distribution of frequencies of 10-nt long motifs within RNA molecules. f_{expected} is the theoretical frequency of a given 10-mer, $1/4^{10} = 9.54\times10^{-7}$.

(C) Canonical sites have contiguous pairing (vertical bar) to the miRNA seed (bold), and some include an additional match to miRNA nucleotide 8 or an A opposite miRNA nucleotide g1. B indicates not A, i.e., C, G, or U.

(D) Enrichment of canonical sites at each position (counted from the 5′ end) within the randomized region of RNA molecules. We note that cytosine—the last nucleotide of the 5′ constant region and the t8 nucleotide of let-7a, miR-34b and miR-449a canonical binding sites—is adjacent to a favorable dinucleotide context (McGeary et al., 2019), yielding ~2–3-fold greater enrichment of sites starting at this position.

(E) Rastergram summaries of traces of individual target molecules, each in a single row and sorted according to their arrival time. Blue intervals indicate binding events. Above: representative fluorescence intensity time traces for miRISC (turquoise) binding RNA targets (magenta) with different extents of complementarity to the miRNA guide. Gray, background levels of green fluorescence; black,binding events.

(F) Comparison of RBNS results with and without DNA blocking oligonucleotides. Enrichment of miR-449a canonical sites was 2.5–5-fold greater when DNA blockers were used, reflecting a decreased fraction of RNA with no binding site (right).

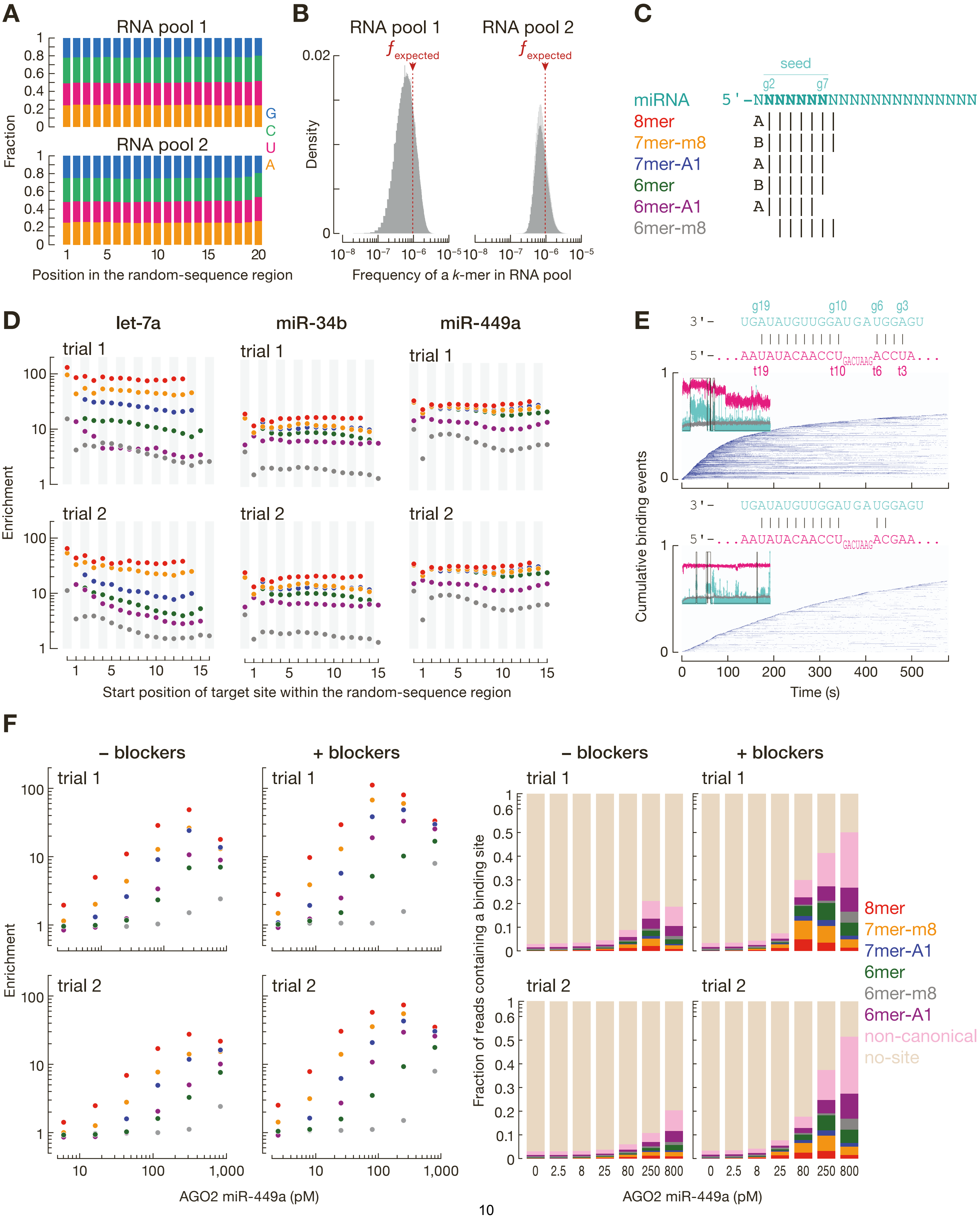
A
RNA pool 1
RNA pool 2
Fraction
G
C
U
A
Position in the random-sequence region
B
RNA pool 1
RNA pool 2
f expected
Density
Frequency of a k-mer in RNA pool
C
seed
g2
g7
miRNA
5'-NNNNNNNNNNNNNNNNNNNNN
8mer
7mer-m8
7mer-A1
6mer
6mer-A1
6mer-m8
D
let-7a
miR-34b
miR-449a
trial 1
trial 2
Enrichment
Start position of target site within the random-sequence region
E
g19
g10
g6
g3
3'- UGAUAUGUUGGAUGAUGGAGU
5'-...AAUAUACAACCUGACUAAGACCUA...
t19
t10
t6
t3
3'- UGAUAUGUUGGAUGAUGGAGU
5'-...AAUAUACAACCUGACUAAGACGAA...
Cumulative binding events
Time (s)
F
– blockers
+ blockers
trial 1
trial 2
Enrichment
AGO2 miR-449a (pM)
Fraction of reads containing a binding site
8mer
7mer-m8
7mer-A1
6mer
6mer-m8
6mer-A1
non-canonical
no-site
AGO2 miR-449a (pM)

Figure S2. RBNS biases relative to technical procedures, Related to Figure 3

(A) Overview of RNA Bind-n-Seq using pull-down on magnetic beads coupled to anti-FLAG antibody.

(B) Enrichment profile of canonical let-7a sites observed at each of the five miRISC concentrations in RBNS including pull-down step, determined as ratio of site frequency in protein sample over site frequency in the RNA pool.

(C) Comparison of binding sites identified by de novo discovery in RBNS including double-filter or pull-down step. Minus sign indicates sites that were not identified by de novo site analysis.

(D) Comparison of dissociation constants estimated from RBNS including double-filter or pull-down step. Error bars indicate 95% confidence intervals on the median. Indicated are the site types showing >2-fold difference. The solid orange and red lines indicate 2-fold and 10-fold difference, respectively. Dashed diagonal lines show $y = x$.

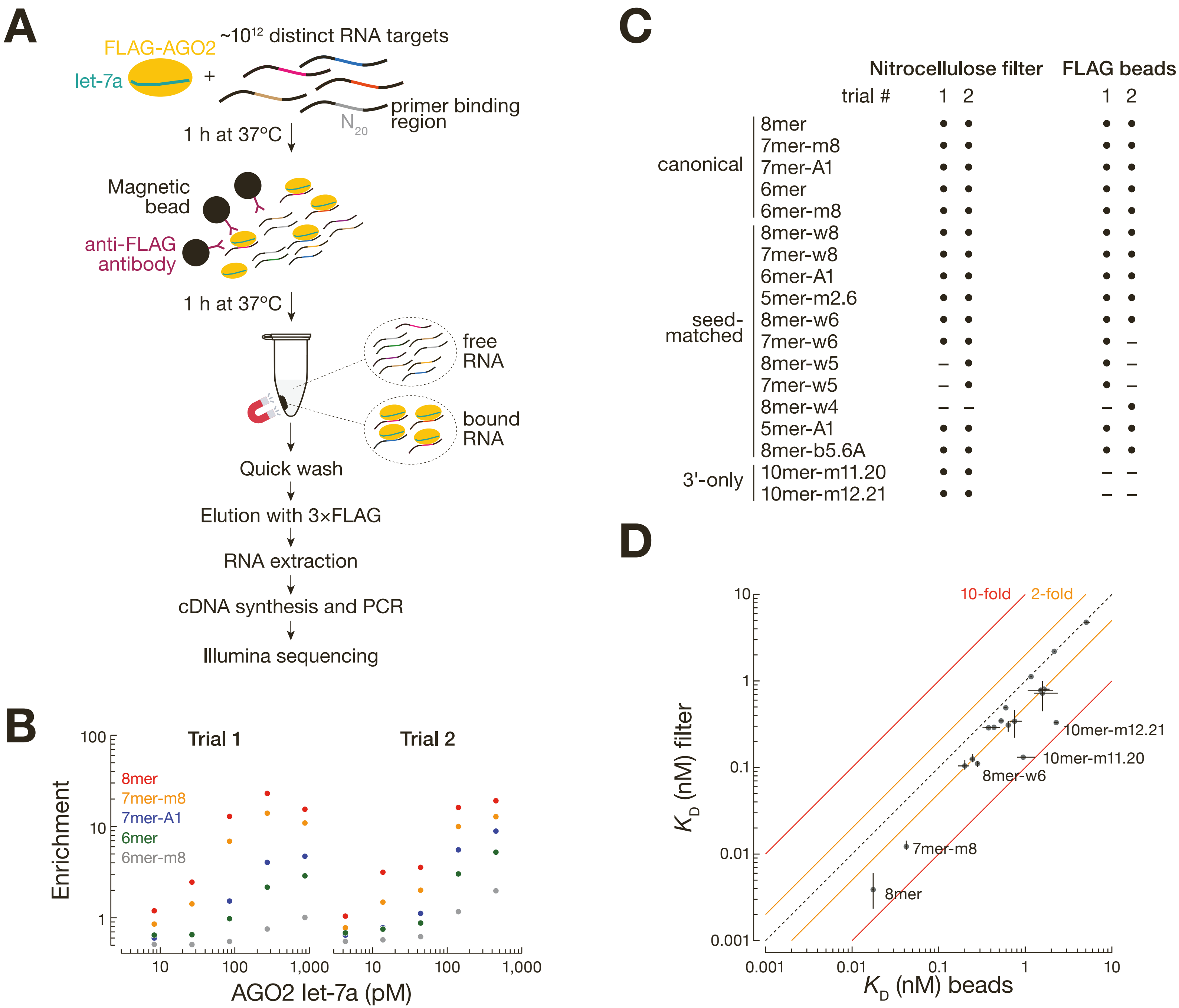
A
FLAG-AGO2
let-7a
+
~10^12 distinct RNA targets
primer binding region
N20
1 h at 37°C
Magnetic bead
anti-FLAG antibody
1 h at 37°C
free RNA
bound RNA
Quick wash
Elution with 3×FLAG
RNA extraction
cDNA synthesis and PCR
Illumina sequencing
B
Trial 1
Trial 2
8mer
7mer-m8
7mer-A1
6mer
6mer-m8
Enrichment
AGO2 let-7a (pM)
C
Nitrocellulose filter
FLAG beads
trial #
canonical
seed-matched
3'-only
8mer
7mer-m8
7mer-A1
6mer
6mer-m8
8mer-w8
7mer-w8
6mer-A1
5mer-m2.6
8mer-w6
7mer-w6
8mer-w5
7mer-w5
8mer-w4
5mer-A1
8mer-b5.6A
10mer-m11.20
10mer-m12.21
D
10-fold
2-fold
K_D (nM) filter
K_D (nM) beads
10mer-m12.21
10mer-m11.20
8mer-w6
7mer-m8
8mer

Figure S3. RBNS biases relative to protein concentration and significance criteria for de novo site discovery, Related to Figure 3

(A) Enrichment of reads containing canonical let-7a sites in no-RISC and guide-only binding reactions. Dashed line indicates $y = x$.

(B) Comparison of binding sites identified by de novo discovery when using binding reactions of different miRISC concentrations.

(C) Illustration of de novo site discovery algorithm, using enrichment threshold instead of *Z*-score threshold presented in Figure 3C.

(D) Comparison of binding sites identified by de novo discovery in RBNS including double-filter or pull-down step when using enrichment threshold.

(E) Distribution of *Z*-scores in RBNS data including double-filter or pull-down step and choice of the *Z*-score for significance.

A

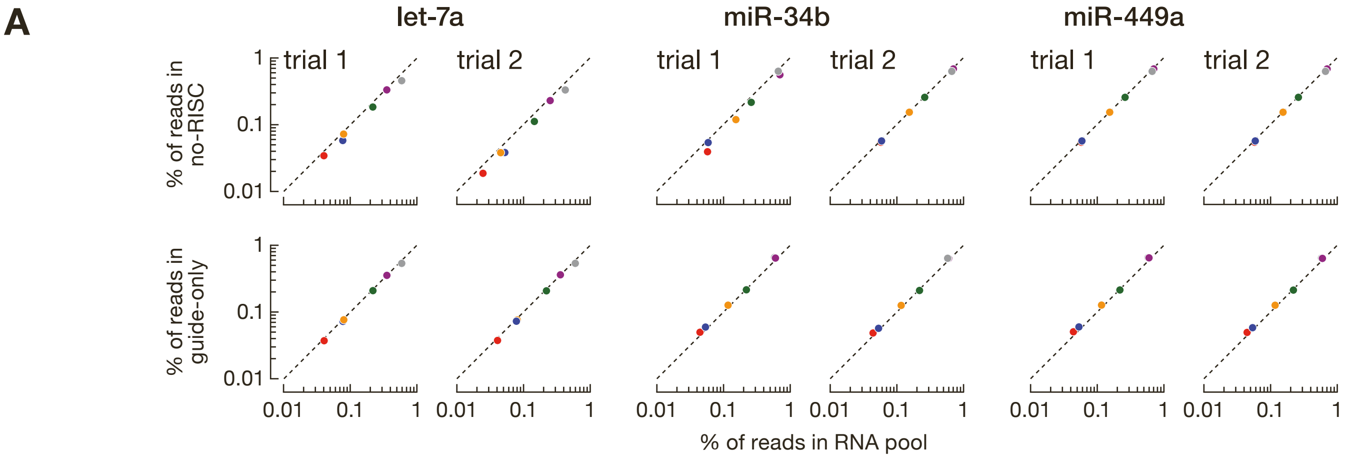

B

	8 pM	25 pM	80 pM	253 pM	800 pM
8mer	•	•	•	•	•
7mer-m8	•	•	•	•	•
7mer-A1	–	•	•	•	•
6mer	–	–	•	•	•
6mer-A1	–	–	•	•	•
6mer-m8	–	–	•	•	•
10mer-m11.20	–	–	•	•	•
8mer-w6	–	–	•	•	•
8mer-w8	–	–	–	•	•
7mer-w8	–	–	–	–	•
7mer-w6	–	–	–	–	•
10mer-m12.21	–	–	–	–	•
8mer-b5.6A	–	–	–	–	•
5mer-m2.6	–	–	–	–	•
5mer-A1	–	–	–	–	•

C

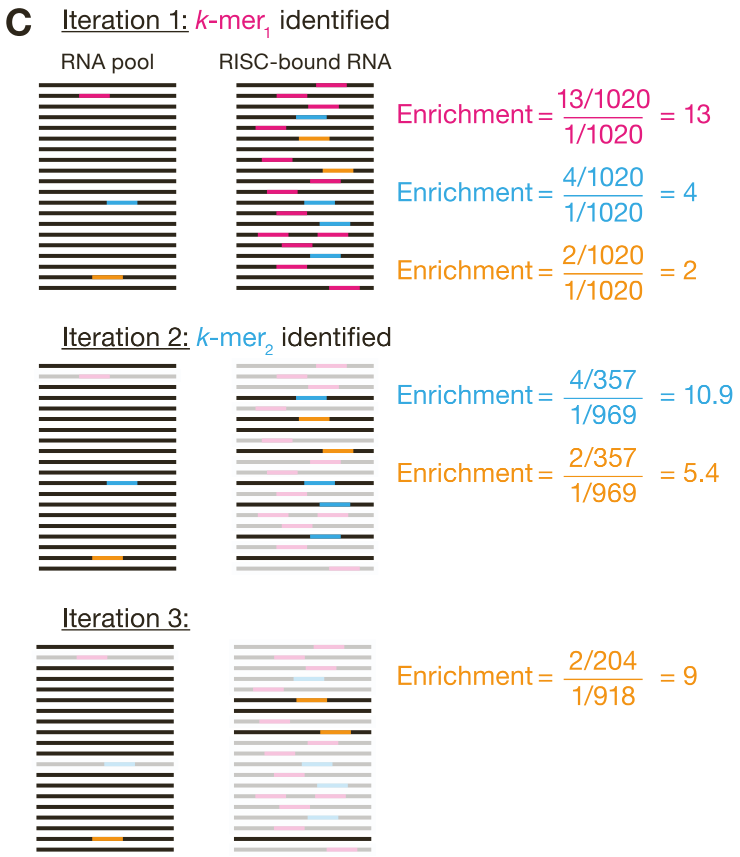

D

		nitrocellulose filter		FLAG beads	
	trial #	1	2	1	2
canonical	8mer	•	•	•	•
	7mer-m8	•	•	•	•
	7mer-A1	•	•	•	•
	6mer	•	•	–	–
	6mer-m8	•	•	–	–
seed-matched	8mer-w8	•	•	–	•
	7mer-w8	•	•	–	–
	6mer-A1	•	•	–	–
	5mer-m2.6	•	•	–	–
	8mer-w6	•	•	•	–
	7mer-w6	•	•	–	–
	8mer-w4	•	•	–	–
	8mer-w5	•	•	–	–
	7mer-w5	•	•	–	–
	5mer-A1	•	•	–	–
	8mer-b5.6A	•	•	–	–
3'-only	10mer-m11.20	•	•	–	–
	10mer-m12.21	•	•	–	–

E

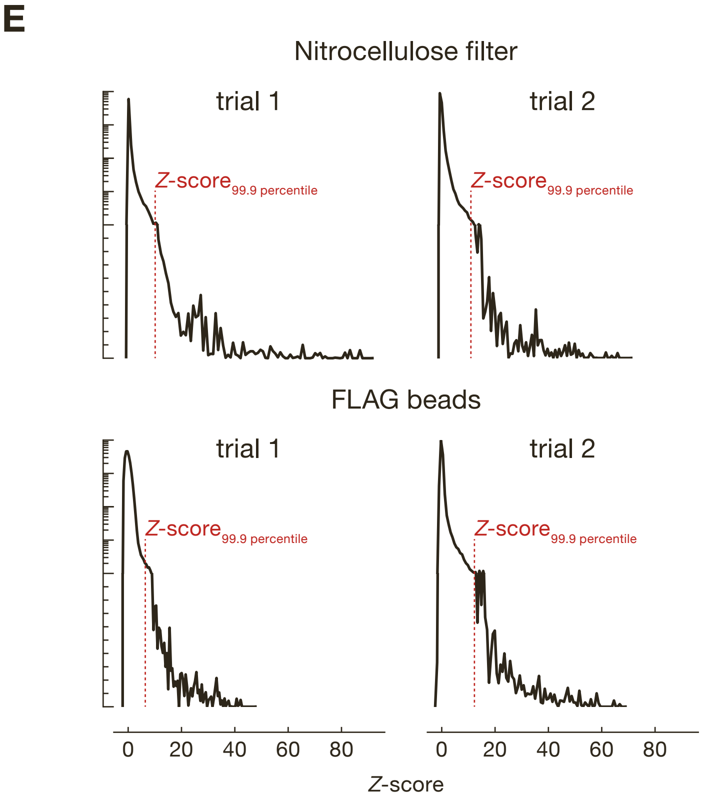

Figure S4. Estimation of K_D values by RBNS is robust and reproducible, Related to Figure 4

(A–B) Testing K_D estimation with simulated data. RBNS data were modeled by simulating miRISC binding to RNA pool containing four binding sites and no-site molecules. Stock concentration of miRISC was equal to 2.1 nM (dataset 1) or 8.1 nM (datasets 2 and 3). Each simulated RBNS experiment contained six binding reactions—five logarithmically spaced miRISC dilutions and a no-RISC binding reaction—and RNA pool at 100 nM. Background was set to 0.1 nM and $K_{D,\ nosite}$ was set to 5 nM. **(A)** Convergence of a representative MLE fit. **(B)** Each fitting optimization was performed 1,000 times using a different set of initial parameter values. Intervals correspond to 95% confidence intervals on the median.

(C) To estimate the robustness of fitted parameters, maximum likelihood estimation was performed on sub-datasets where each sample was removed one by one. Pearson's *r* was calculated for each of the fifteen pairwise possibilities.

(D) K_D values for t2–t8, t2–t7 and t2–t6 let-7a targets with different t1 nucleotides. Adenine (orange), uridine (magenta), cytidine (cyan), and guanosine (blue).

(E, F) Measuring k_{on}, k_{off} and K_D values of 6mer, 6mer-A1 and 6mer-m8 let-7a sites by CoSMoS. **(F)** Representative fluorescence intensity time traces of miRISC (turquoise) binding RNA target (magenta) with different extents of complementarity to the miRNA guide. Gray indicates background levels of green fluorescence, whereas the black line denotes binding events. Cumulative fraction of let-7a-guided AGO2 molecules binding for the first time to a single RNA target and dwell time distribution of these binding events. Data points are plotted in black, and the curve in blue shows the rate of binding or departure after correcting for non-specific association of miRISC with the glass surface. k_{on} and k_{off} values were derived from data collected from >900 individual RNA target molecules; standard error from bootstrapping is reported.

A

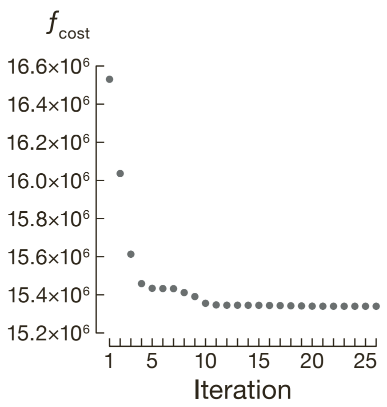

B

		[RISC]$_{stock}$	$K_{D,nosite}$	background
**Set 1**	Ground truth	2.1 nM	5 nM	0.1 nM
	Estimated	3.7 nM [3.4, 3.9]	2.3 nM [2.2, 2.4]	0.09 nM [0.09, 0.10]
**Set 2**	Ground truth	8.1 nM	5 nM	0.1 nM
	Estimated	8.7 nM [8.1, 9.3]	1.5 nM [1.4, 1.5]	0.03 nM [0.03, 0.03]
**Set 3**	Ground truth	8.1 nM	5 nM	0.1 nM
	Estimated	25 nM [25, 26]	3.7 nM [3.7, 3.8]	0.05 nM [0.05, 0.06]

C

	**0 pM**	**8 pM**	**25 pM**	**80 pM**	**250 pM**
**8 pM**	0.999				
**25 pM**	1.00	0.999			
**80 pM**	1.00	1.00	1.00		
**250 pM**	1.00	0.999	1.00	1.00	
**800 pM**	0.979	0.969	0.978	0.974	0.979

E

**Binding site**	k_{on} (M^{-1} s^{-1})	k_{off} (s^{-1})	K_D (nM)
6mer	1.00 ± 0.02 × 10^8	0.040 ± 0.001	0.40 ± 0.02
6mer-m8	1.40 ± 0.02 × 10^8	0.290 ± 0.002	2.1 ± 0.04
6mer-A1	1.50 ± 0.01 × 10^8	0.330 ± 0.002	2.2 ± 0.03

D

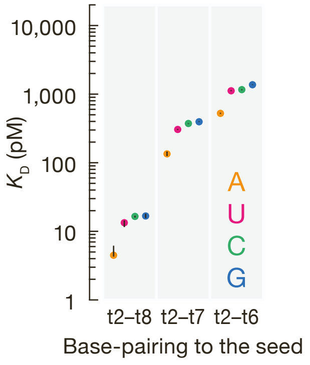

F

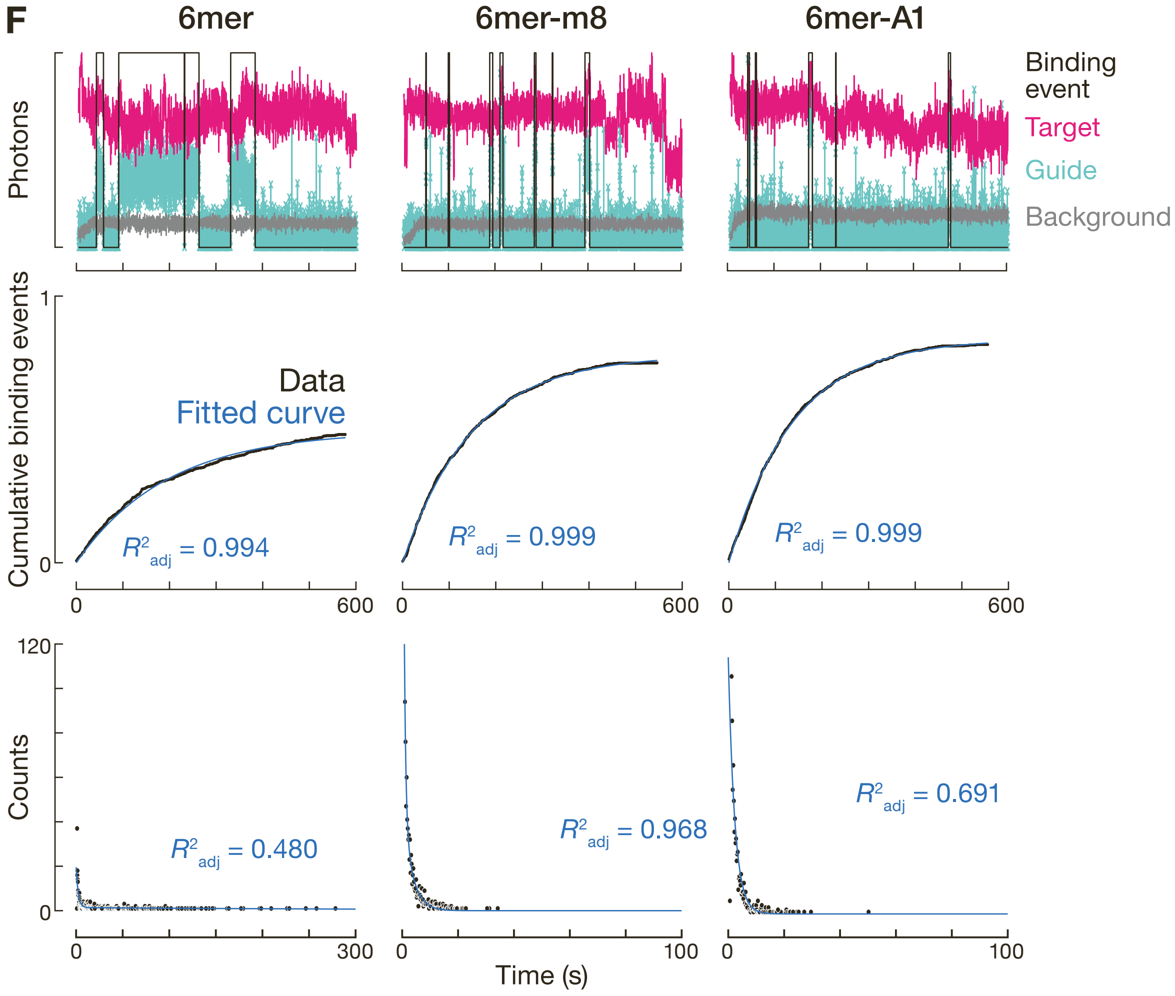

Figure S5. Sequence-specific differences in miRNA affinities, Related to Figure 6

(A) Dissociation constants fitted for AGO2 miR-1, lsy-6 and miR-155 RBNS from a prior study (McGeary et al., 2019). On the left is pairing of enriched sites identified by de novo site discovery. Enrichment profile of canonical sites is shown on the right. **(B)** The relationship between the observed and predicted K_D values for t2–t8, t2–t7, t2–t6 and t3–t8 binding sites of eight miRNAs. While affinity increased with increased predicted pairing stability, the correlation between measured affinity and affinity predicted by nearest-neighbor free energy was significant only for 7mer-m8 binding sites. Nearest neighbor analysis predicts that miR-449, the most stably pairing miRNA in our study, should bind much more tightly to its targets than lsy-6: >17,500-fold for a 6mer, >8,300-fold for a 5mer-m2.6, and >4,200-fold for 6mer-m8 sites. Yet when bound to AGO2, the actual ratios of the affinities of the two miRNAs are quite small: 6.9-fold for a 6mer, 2.6-fold for a 5mer-m2.6, and 0.97-fold for 6mer-m8 sites. In other words, AGO2 reduces the dynamic range of intrinsic differences in seed-pairing stabilities by several orders of magnitude.

Error bars indicate 95% confidence intervals of the median.

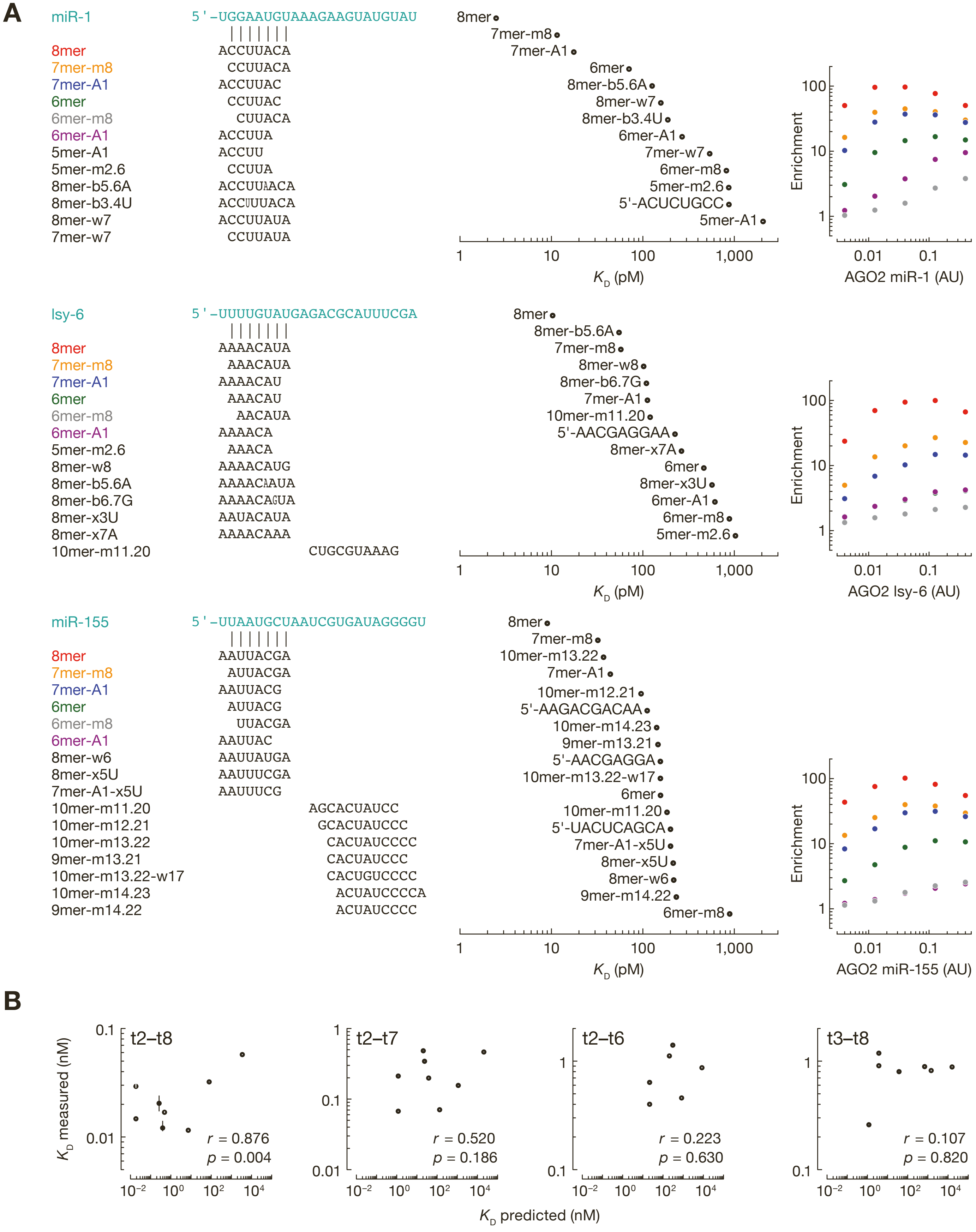
A
miR-1
5'-UGGAAUGUAAAGAAGUAUGUAU
8mer ACCUUACA
7mer-m8 CCUUACA
7mer-A1 ACCUUAC
6mer CCUUAC
6mer-m8 CUUACA
6mer-A1 ACCUUA
5mer-A1 ACCUU
5mer-m2.6 CCUUA
8mer-b5.6A
8mer-b3.4U
8mer-w7 ACCUUAUA
7mer-w7 CCUUAUA
8mer
7mer-m8
7mer-A1
6mer
8mer-b5.6A
8mer-w7
8mer-b3.4U
6mer-A1
7mer-w7
6mer-m8
5mer-m2.6
5'-ACUCUGCC
5mer-A1
1
10
100
1,000
K_D (pM)
Enrichment
100
10
1
0.01
0.1
AGO2 miR-1 (AU)
lsy-6
5'-UUUUGUAUGAGACGCAUUUCGA
8mer AAAACAUA
7mer-m8 AAACAUA
7mer-A1 AAAACAU
6mer AAACAU
6mer-m8 AACAUA
6mer-A1 AAAACA
5mer-m2.6 AAACA
8mer-w8 AAAACAUG
8mer-b5.6A
8mer-b6.7G
8mer-x3U AAUACAUA
8mer-x7A AAAACAAA
10mer-m11.20 CUGCGUAAAG
8mer
8mer-b5.6A
7mer-m8
8mer-w8
8mer-b6.7G
7mer-A1
10mer-m11.20
5'-AACGAGGAA
8mer-x7A
6mer
8mer-x3U
6mer-A1
6mer-m8
5mer-m2.6
1
10
100
1,000
K_D (pM)
Enrichment
100
10
1
0.01
0.1
AGO2 lsy-6 (AU)
miR-155
5'-UUAAUGCUAAUCGUGAUAGGGGU
8mer AAUUACGA
7mer-m8 AUUACGA
7mer-A1 AAUUACG
6mer AUUACG
6mer-m8 UUACGA
6mer-A1 AAUUAC
8mer-w6 AAUUAUGA
8mer-x5U AAUUUCGA
7mer-A1-x5U AAUUUCG
10mer-m11.20 AGCACUAUCC
10mer-m12.21 GCACUAUCCC
10mer-m13.22 CACUAUCCCC
9mer-m13.21 CACUAUCCC
10mer-m13.22-w17 CACUGUCCCC
10mer-m14.23 ACUAUCCCCA
9mer-m14.22 ACUAUCCCC
8mer
7mer-m8
10mer-m13.22
7mer-A1
10mer-m12.21
5'-AAGACGACAA
10mer-m14.23
9mer-m13.21
5'-AACGAGGA
10mer-m13.22-w17
6mer
10mer-m11.20
5'-UACUCAGCA
7mer-A1-x5U
8mer-x5U
8mer-w6
9mer-m14.22
6mer-m8
1
10
100
1,000
K_D (pM)
Enrichment
100
10
1
0.01
0.1
AGO2 miR-155 (AU)
B
K_D measured (nM)
t2–t8
r = 0.876
p = 0.004
t2–t7
r = 0.520
p = 0.186
t2–t6
r = 0.223
p = 0.630
t3–t8
r = 0.107
p = 0.820
K_D predicted (nM)

Figure S6. Dissociation constants of enriched sites estimated for miR-7, miR-124, miR-34b, miR-449a and let-7a RBNS datasets, Related to Figure 6

Dissociation constants fitted for AGO2 miR-7 and miR-124 RBNS from a prior study (McGeary et al., 2019) **(A)**, and miR-34b **(B)**, miR-449a **(C)** and let-7a **(D)** from this study. Error bars indicate 95% confidence intervals on the median. **(A, B and D)** Pairing is shown for enriched sites identified by de novo site discovery. **(C)** Pairing of enriched sites as in **(B)**. **(A and B)** Enrichment profile of canonical sites is shown on the right.

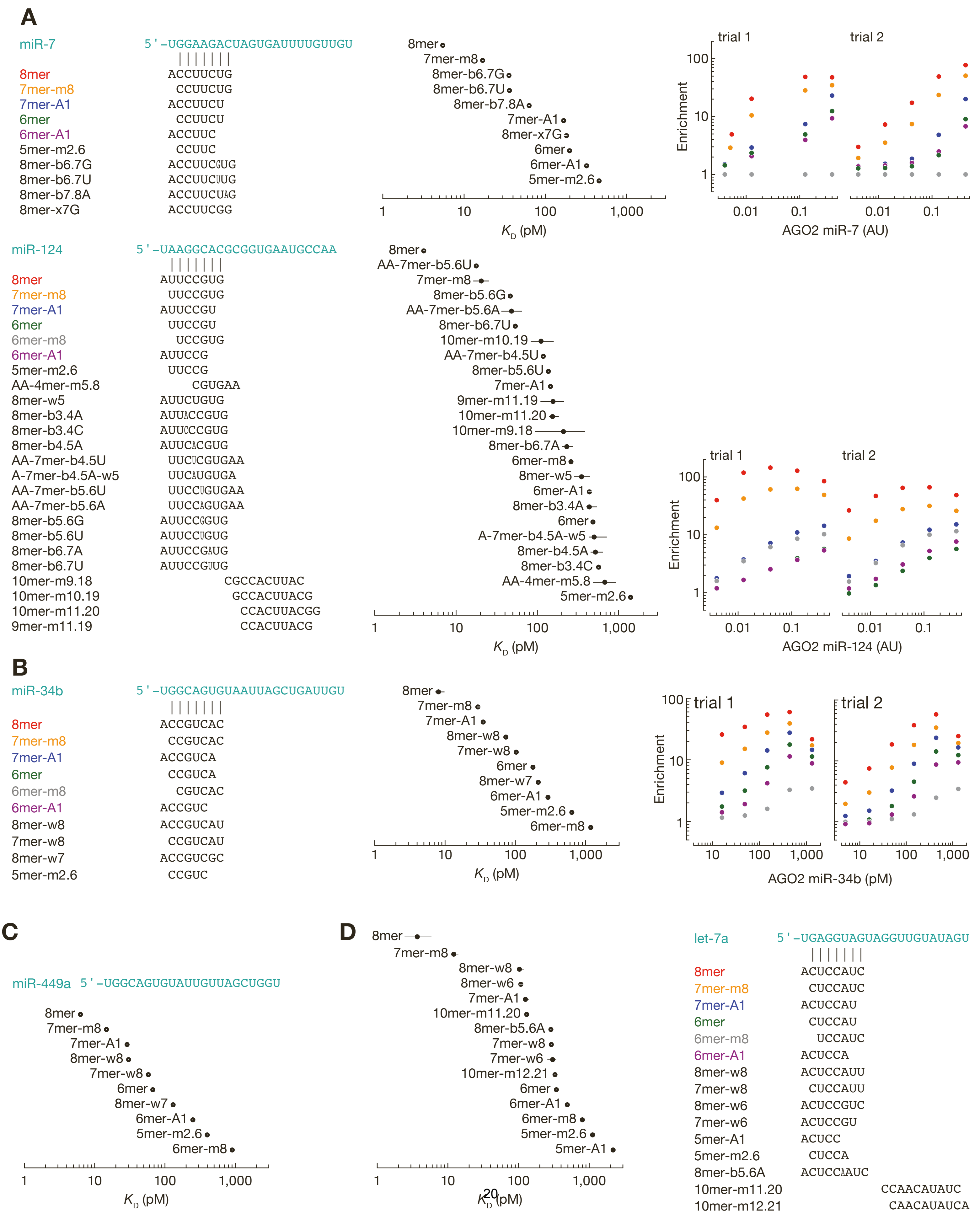
A
miR-7 5'-UGGAAGACUAGUGAUUUUGUUGU
8mer ACCUUCUG
7mer-m8 CCUUCUG
7mer-A1 ACCUUCU
6mer CCUUCU
6mer-A1 ACCUUC
5mer-m2.6 CCUUC
8mer-b6.7G ACCUUCGUG
8mer-b6.7U ACCUUCUUG
8mer-b7.8A ACCUUCUAG
8mer-x7G ACCUUCGG
8mer
7mer-m8
8mer-b6.7G
8mer-b6.7U
8mer-b7.8A
7mer-A1
8mer-x7G
6mer
6mer-A1
5mer-m2.6
1
10
100
1,000
KD (pM)
trial 1
trial 2
100
10
1
Enrichment
0.01
0.1
AGO2 miR-7 (AU)
miR-124 5'-UAAGGCACGCGGUGAAUGCCAA
8mer AUUCCGUG
7mer-m8 UUCCGUG
7mer-A1 AUUCCGU
6mer UUCCGU
6mer-m8 UCCGUG
6mer-A1 AUUCCG
5mer-m2.6 UUCCG
AA-4mer-m5.8 CGUGAA
8mer-w5 AUUCUGUG
8mer-b3.4A AUUACCGUG
8mer-b3.4C AUUCCCGUG
8mer-b4.5A AUUCACGUG
AA-7mer-b4.5U UUCUCGUGAA
A-7mer-b4.5A-w5 UUCAUGUGA
AA-7mer-b5.6U UUCCUGUGAA
AA-7mer-b5.6A UUCCAGUGAA
8mer-b5.6G AUUCCGGUG
8mer-b5.6U AUUCCUGUG
8mer-b6.7A AUUCCGAUG
8mer-b6.7U AUUCCGUUG
10mer-m9.18 CGCCACUUAC
10mer-m10.19 GCCACUUACG
10mer-m11.20 CCACUUACGG
9mer-m11.19 CCACUUACG
8mer
AA-7mer-b5.6U
7mer-m8
8mer-b5.6G
AA-7mer-b5.6A
8mer-b6.7U
10mer-m10.19
AA-7mer-b4.5U
8mer-b5.6U
7mer-A1
9mer-m11.19
10mer-m11.20
10mer-m9.18
8mer-b6.7A
6mer-m8
8mer-w5
6mer-A1
8mer-b3.4A
6mer
A-7mer-b4.5A-w5
8mer-b4.5A
8mer-b3.4C
AA-4mer-m5.8
5mer-m2.6
1
10
100
1,000
KD (pM)
trial 1
trial 2
100
10
1
Enrichment
0.01
0.1
AGO2 miR-124 (AU)
B
miR-34b 5'-UGGCAGUGUAAUUAGCUGAUUGU
8mer ACCGUCAC
7mer-m8 CCGUCAC
7mer-A1 ACCGUCA
6mer CCGUCA
6mer-m8 CGUCAC
6mer-A1 ACCGUC
8mer-w8 ACCGUCAU
7mer-w8 CCGUCAU
8mer-w7 ACCGUCGC
5mer-m2.6 CCGUC
8mer
7mer-m8
7mer-A1
8mer-w8
7mer-w8
6mer
8mer-w7
6mer-A1
5mer-m2.6
6mer-m8
1
10
100
1,000
KD (pM)
100
10
1
Enrichment
trial 1
trial 2
10
100
1,000
AGO2 miR-34b (pM)
C
miR-449a 5'-UGGCAGUGUAUUGUUAGCUGGU
8mer
7mer-m8
7mer-A1
8mer-w8
7mer-w8
6mer
8mer-w7
6mer-A1
5mer-m2.6
6mer-m8
1
10
100
1,000
KD (pM)
D
8mer
7mer-m8
8mer-w8
8mer-w6
7mer-A1
10mer-m11.20
8mer-b5.6A
7mer-w8
7mer-w6
10mer-m12.21
6mer
6mer-A1
6mer-m8
5mer-m2.6
5mer-A1
1
10
100
1,000
KD (pM)
let-7a 5'-UGAGGUAGUAGGUUGUAUAGU
8mer ACUCCAUC
7mer-m8 CUCCAUC
7mer-A1 ACUCCAU
6mer CUCCAU
6mer-m8 UCCAUC
6mer-A1 ACUCCA
8mer-w8 ACUCCAUU
7mer-w8 CUCCAUU
8mer-w6 ACUCCGUC
7mer-w6 ACUCCGU
5mer-A1 ACUCC
5mer-m2.6 CUCCA
8mer-b5.6A ACUCCAAUC
10mer-m11.20 CCAACAUAUC
10mer-m12.21 CAACAUAUCA

Figure S7. Estimation of k_{on} and k_{off} values by RBNS, Related to Figure 7

(A) Overview of RNA Bind-n-Seq extended to kinetic studies.

(B) Testing k_{on} and k_{off} estimation with simulated data. RBNS data were modeled by simulating miRISC binding to RNA pool containing four binding sites and no-site molecules. Total miRISC concentration was equal to 0.15 nM. Background was set to 0.1 nM. Dataset 1 contains binding sites with similar k_{off} but different k_{on} values. Dataset 2 contains binding sites with similar k_{on} but different k_{off} values. Estimated absolute values of kinetic parameters are not accurately recovered from the ground truth.

(C) k_{on} values for let-7a 7mer-A1, 6mer-A1 and 5mer-A1 sites separated into 4^4 = 256 sites according to the dinucleotide sequences immediately flanking the 5′ and the 3′ ends of the site. To increase statistical power, we classified these 256 sites into 5 bins based on the number of A/U and G/C nucleotides and determined k_{on} for each. Error bars indicate 95% confidence intervals on the median. G and C flanking nucleotides reduced k_{on}, likely by stabilizing RNA secondary structures that occlude the binding site. This effect of flanking dinucleotides on k_{on} was consistent among the three site types, but was greater for shorter, lower-affinity sites: for the 7mer-A1 sites, the most favorable (A/U-rich) contexts were just twice as fast as the most detrimental (G/C-rich), but for the 5mer-A1 sites, the A/U-rich contexts were 29-fold faster than the G/C-rich. Similarly, k_{on} for a 7mer-A1 bracketed by di-adenosines was 7-fold faster than when flanked by di-guanosines; for the 5mer-A1 flanked by di-adenosines was ~93-fold faster than with di-guanosines.

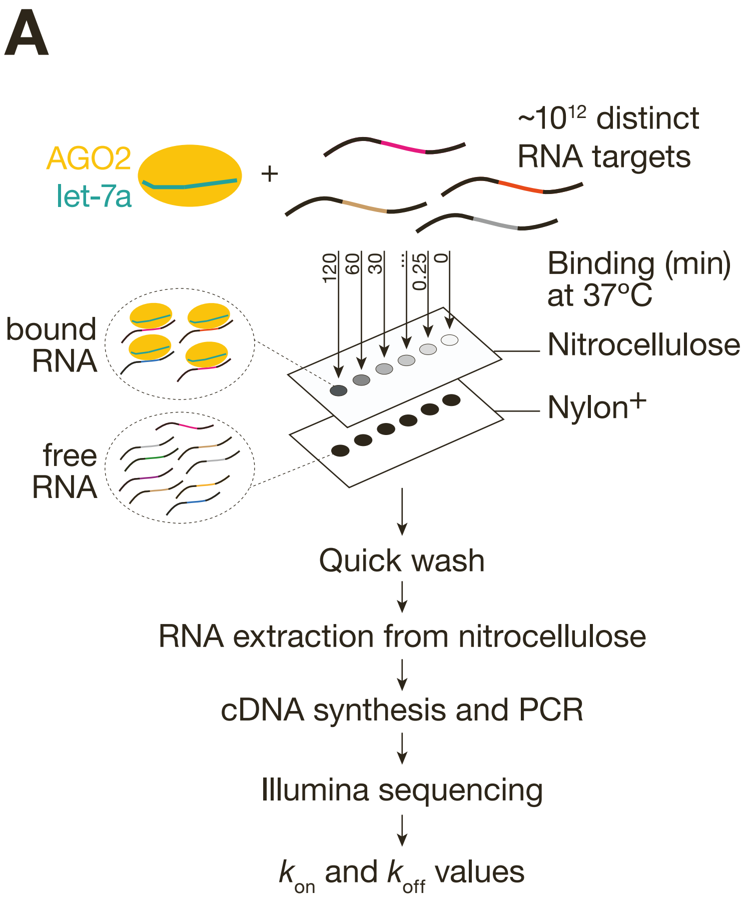

B

		Dataset 1		Dataset 2	
		True	Estimated	True	Estimated
k_{on} (nM^{-1} s^{-1})	Site 1	0.1	5.04	0.1	2.08
	Site 2	0.01	0.543	0.1	2.01
	Site 3	0.001	0.056	0.1	1.95
	Site 4	0.0001	0.007	0.1	5.72
	No-site	0.0001	0.026	0.001	0.010
k_{off} (s^{-1})	Site 1	0.0001	0.001	0.00001	0.0005
	Site 2	0.01	0.285	0.001	0.048
	Site 3	0.01	0.323	0.001	0.495
	Site 4	0.01	0.363	0.1	11.9
	No-site	10	18.1	10	40.5
background (nM)		0.1	0.150	0.1	0.088

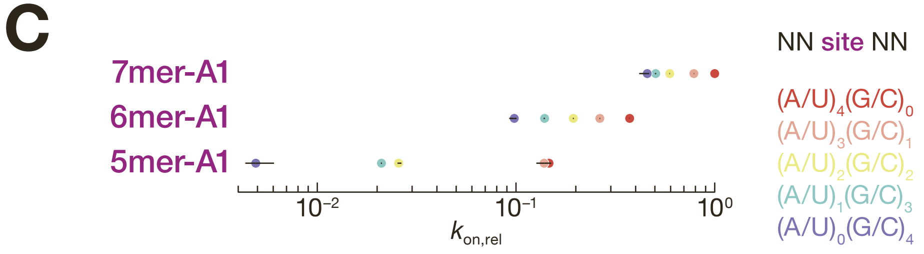

Supplement: Document S1. Figures S1–S7 and Tables S1–S4 [file mmc1.pdf]
