# Supplementary material for: Principles and pitfalls of high-throughput analysis of microRNA-binding thermodynamics and kinetics by RNA Bind-n-Seq
Source: Cell Rep Methods. 2022 Mar 18;2(3):100185. doi: 10.1016/j.crmeth.2022.100185 (PMC9017153; doi:10.1016/j.crmeth.2022.100185)

Cell Reports Methods

Article

Principles and pitfalls of high-throughput analysis of microRNA-binding thermodynamics and kinetics by RNA Bind-n-Seq

Graphical abstract

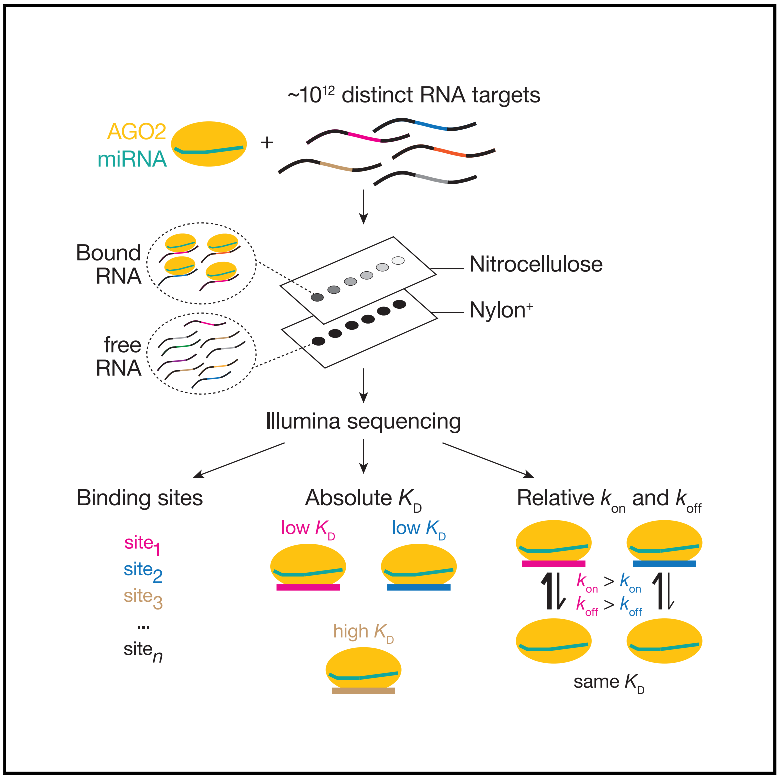

Highlights

- Several factors may influence the outcome of the RBNS analysis
- An analytical strategy estimates absolute binding affinities from RBNS data
- A simple framework to compute relative association and dissociation rate constants
- Absolute affinities of microRNA-directed binding of AGO2 to a variety of sites

Authors

Karina Jouravleva, Joel Vega-Badillo, Phillip D. Zamore

Correspondence

karina.jouravleva@umassmed.edu (K.J.), phillip.zamore@umassmed.edu (P.D.Z.)

In brief

Jouravleva et al. show that, when designed and analyzed with potential biases in mind, RNA Bind-n-Seq provides a high-throughput route to discovering the sequence preferences of RNA-binding proteins, defining their absolute equilibrium binding dissociation constants, and delineating their relative association and dissociation rate constants for sites of disparate sequence and affinity.

Jouravleva et al., 2022, Cell Reports Methods 2, 100185
March 28, 2022 © 2022 The Author(s).
https://doi.org/10.1016/j.crmeth.2022.100185

CellPress

Cell Reports Methods

CellPress
OPEN ACCESS

Article

Principles and pitfalls of high-throughput analysis of microRNA-binding thermodynamics and kinetics by RNA Bind-n-Seq

Karina Jouravleva,[1,*] Joel Vega-Badillo,[1] and Phillip D. Zamore[1,2,3,*]

[1]RNA Therapeutics Institute, University of Massachusetts Medical School, 368 Plantation Street, Worcester, MA 01605, USA
[2]Howard Hughes Medical Institute, University of Massachusetts Medical School, 368 Plantation Street, Worcester, MA 01605, USA
[3]Lead contact
*Correspondence: karina.jouravleva@umassmed.edu (K.J.), phillip.zamore@umassmed.edu (P.D.Z.)
https://doi.org/10.1016/j.crmeth.2022.100185

MOTIVATION The speed at which regulatory proteins find their RNA targets and the time they spend bound to specific regulatory sequence motifs govern the specificity and extent of target repression or activation. RBNS was developed to qualitatively describe the specificity of RNA-binding proteins. Here, we present an analytical strategy to measure absolute affinity values and relative association and dissociation rate constants from RBNS data. The resulting analysis provides a resource for replacing >500 biochemical binding assays in examining physicochemical properties and kinetic behavior of miRNA-guided Argonaute (AGO) proteins. Our quantitative measurements should allow prediction of complex and dynamic miRNA-mRNA regulatory networks.

SUMMARY

RNA Bind-n-Seq (RBNS) is a cost-effective, high-throughput method capable of identifying the sequence preferences of RNA-binding proteins and of qualitatively defining relative dissociation constants. Although RBNS is often described as an unbiased method, several factors may influence the outcome of the analysis. Here, we discuss these biases and present an analytical strategy to estimate absolute binding affinities from RBNS data, extend RBNS to kinetic studies, and develop a framework to compute relative association and dissociation rate constants. As proof of principle, we measured the equilibrium binding properties of mammalian Argonaute2 (AGO2) guided by eight microRNAs (miRNAs) and kinetic parameters for let-7a. The miRNA-binding site repertoires, dissociation constants, and kinetic parameters calculated from RBNS data using our methods correlate well with values measured by traditional ensemble and single-molecule approaches. Our data provide additional quantitative measurements for Argonaute-bound miRNA binding that should facilitate development of quantitative targeting rules for individual miRNAs.

INTRODUCTION

Predicting the regulatory consequences of molecular interactions based on measured biochemical properties is a longstanding goal in biology. The strength of an interaction is quantitatively described by its equilibrium dissociation constant (K_D), the substrate concentration required for half-maximal complex formation. By definition, K_D is the ratio of the rate of complex dissolution, described by the dissociation rate constant, k_{off}, and the rate of complex formation, described by the association rate constant, k_{on}. Interactions with the same affinity may arise from different kinetic behaviors that may vary by orders of magnitude: one set of interactions may be driven by rapid recognition and binding (large k_{on}), while another may be driven by slower k_{on} but increased complex stability (small k_{off}). Knowledge of k_{on} limits the possible mechanisms for target finding, e.g., whether a binding event is diffusion-limited or whether additional proteins can speed the search for high-affinity binding sites. k_{off} determines the lifespan of the binding interaction and thus provides insight into whether a process is likely to be driven by a hit-and-run mechanism or require continued site occupancy to exert a regulatory effect. Thus, the affinities and the dynamics of molecular interaction provide critical information for developing quantitative models of a regulatory network.

Interactions between RNA-binding proteins (RBPs) and mRNAs are dynamic (Müller-McNicoll and Neugebauer, 2013; Licatalosi et al., 2020) and at the core of gene regulation (Moore, 2005). Current approaches to determine thermodynamic and

This is an open access article under the CC BY license (http://creativecommons.org/licenses/by/4.0/).

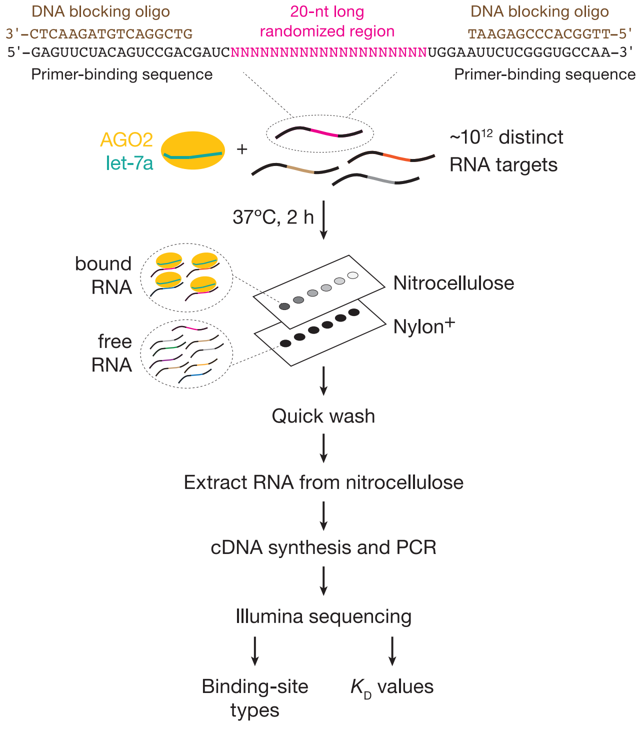

Figure 1. Overview of RNA Bind-n-Seq

kinetic properties generally involve compromises in throughput, comprehensiveness, and quantitative precision. For example, targeted biochemical approaches—including electrophoretic mobility shift assays and fluorescence resonance energy transfer—provide quantitative biophysical measurements but can only interrogate known interactions and are therefore inherently low throughput (Ladbury, 1995; Ladbury and Chowdhry, 1996; Garland, 1996; Schuck, 1997; Frey and Albin, 2001; Erickson et al., 2001, 2003; Hellman and Fried, 2007; Shi and Herschlag, 2009). By contrast, high-throughput sequencing methods that rely on crosslinking and immunoprecipitation yield comprehensive lists of RNA-binding motifs but do not enable quantitative assessment of binding affinities (Licatalosi et al., 2008; König et al., 2010; Zhao et al., 2010; Danan et al., 2016; Sugimoto et al., 2012; Kishore et al., 2011; Campbell and Wickens, 2015). Other strategies provide high-throughput, quantitative information for intermolecular associations but use complicated experimental setups unavailable to many laboratories (Buenrostro et al., 2014; Tome et al., 2014; Nutiu et al., 2011; Maerkl and Quake, 2007; Martin et al., 2012; Sharma et al., 2021).

RNA Bind-n-Seq (RBNS) determines the specificity of proteins for nucleic acids *in vitro* using a single-step binding assay and a high-throughput sequencing readout, making the method widely accessible and cost-effective. Originally developed to identify the repertoire of DNA sequence motifs by zinc-finger proteins and calculate their relative binding affinities (Zykovich et al., 2009), it was subsequently applied to RBPs (Lambert et al., 2014; Taliaferro et al., 2016; Dominguez et al., 2018; Hale et al., 2018; McGeary et al., 2019; Van Nostrand et al., 2020). Experimental procedures for RBNS and the computational strategy to identify RBP binding sites and their relative K_D values have been presented previously (Lambert et al., 2015; McGeary et al., 2019).

Here, we revisit RBNS, providing practical guidelines for performing the assay and highlighting potential biases and pitfalls influencing the outcome of RBNS analysis. We report a novel computational approach that extracts absolute K_D values from RBNS data, providing a high-throughput route to quantitatively describe intermolecular interactions. Finally, we extend RBNS to measure relative k_{on} and k_{off} values, enabling a deeper understanding of reversible bimolecular interactions. To test our method, we use published (McGeary et al., 2019) and new data for target binding by mammalian AGO2 loaded with different miRNAs. *In vivo*, miRNAs guide AGO2 to repress gene expression (Liu et al., 2004; Meister et al., 2004; Baek et al., 2008; Selbach et al., 2008; Guo et al., 2010; Doench and Sharp, 2004; Hendrickson et al., 2009; Huntzinger and Izaurralde, 2011; Bazzini et al., 2012). Unlike most RBPs (69 of 78 assayed), which have detectable binding affinity for just one or two sequence motifs (Van Nostrand et al., 2020), AGO proteins take their binding specificity from their 21-nt-long miRNA guides. Consequently, AGO proteins can bind a variety of sites with affinities ranging from ∼1 pM to ∼10 nM. AGO proteins are ideal for illustrating the biases inherent in RBNS, discussing experimental considerations, and benchmarking our strategies for measuring absolute K_D values and relative k_{on} and k_{off} values.

RESULTS

Overview of RNA Bind-n-Seq

RBNS begins with incubation of a purified RBP with a large pool of RNA molecules, each containing a region of random sequence (Figure 1). After reaching equilibrium, protein-bound RNA is separated from unbound RNA and then extracted from the nitrocellulose membrane, reverse transcribed, amplified, and sequenced. Analysis of sequencing data readily identifies a list of preferentially bound sequence motifs. Performing multiple individual binding reactions across a broad range of RBP concentration allows measurement of relative K_D (Lambert et al., 2015).

Experimental design considerations

RISC purification and choice of concentration range

Assembly and purification of miRISC (RNA-induced silencing complex) comprising purified human or mouse AGO2 and a synthetic miRNA guide has been described elsewhere (Figure 2A) (Flores-Jasso et al., 2013). Because different miRISC preparations can have different percent activities for the same amount of protein, a titration experiment to quantify the concentration of binding-competent miRISC is a key step for determining the protein concentrations to use in RBNS reactions. We estimate total miRISC concentration by northern blot and use double-filter binding assays to measure the equilibrium binding of active miRISC with a high-affinity RNA target (Figures 2B and 2C). Concentration of RNA target is chosen to be much greater than its measured K_D, and miRISC concentration is varied by an order of magnitude above and below the target

CellPress
OPEN ACCESS

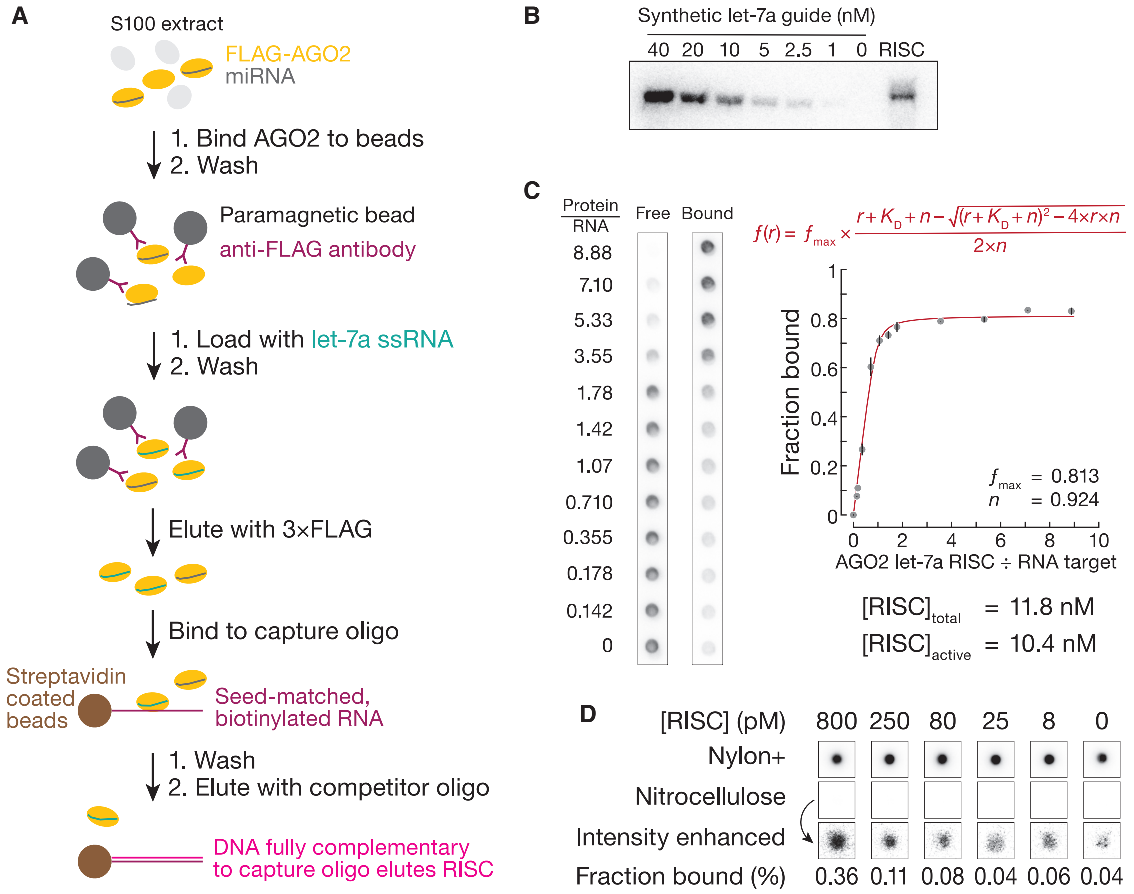

Figure 2. Sequential steps of RBNS

(A) Purification of miRISC containing a single, unique small RNA guide.

(B) Total concentration of purified miRISC is estimated by northern blot.

(C) Concentration of binding-competent miRISC is measured by a titration experiment. K_D is the apparent dissociation constant, r is the molar ratio of [RISC] to [RNA], n is the stoichiometric equivalence point, f is the fraction bound, and f_{max} is the maximum fraction bound. Error bars report propagated SD.

(D) After the binding step of RBNS, the two membranes are separated, imaged, and quantified.

concentration. The titration data are then fit to a quadratic equation (Figure 2C). The stoichiometry of the bound complex is 1:1 (Wee et al., 2012); therefore, the breakpoint in fraction bound versus the ratio of protein to ligand indicates the amount of active miRISC.

To examine binding to both high- and low-affinity sites, RBP concentrations in RBNS reactions should span and exceed K_D values of those sites. miRISC has subnanomolar affinity for its canonical sites—mouse AGO2 loaded with let-7a has K_D ~10 pM for an 8mer site but ~200 pM for a 6mer (Becker et al., 2019; Flores-Jasso et al., 2013; Salomon et al., 2015). Therefore, we surveyed logarithmically spaced miRISC concentrations from ~5 pM to ~1 nM. In addition, we performed a no-RISC binding experiment to detect potential method-specific biases in background binding (Figure 2D).

RNA design

The length of the random sequence region of RNA ligands is an important aspect of RBNS design (Lambert et al., 2015). miRNA canonical binding sites are 6–8-nt long, whereas noncanonical sites can be 11–12 nt long (Shin et al., 2010). To capture binding at both, we randomized 20 nt, an RNA pool of $4^{20} = 1.1 \times 10^{12}$ distinct RNA sequences. Each binding reaction was carried out with 2 pmol of RNA—1.2×10^{12} molecules; thus, the pool theoretically contained $>10^4$ copies of each of the $4^{12} = 1.7 \times 10^7$ possible 12mers and $>10^8$ copies of each of the $4^6 = 4.1 \times 10^3$ possible 6mers. High-throughput sequencing of the RNA pool generally confirmed these expectations and showed an overall balanced base composition, albeit with a slightly reduced frequency of guanine and slightly higher than expected frequency of cytosine (Figure S1A). In addition, some 10mers were depleted 10-fold, and others were over-represented by 5-fold compared with expected frequency (Figure S1B). Such departures from randomness in the RNA pool is corrected for by calculating the ratio of the frequency of each 10mer protein-bound RNA to that in the starting pool.

In typical RBNS experiments, the random sequence RNA region is flanked by constant primer-binding regions used for sequencing (Figure 1). This design simplifies library preparation, avoids biases that can result from RNA ligation, and ensures that any RNA carried over from protein purification will not

contaminate the sequenced library. However, fixed primer-binding sequences can affect the secondary structure ensemble of the RNA pool (Cook et al., 2017) and bias interpretation of RBNS assays with RBPs that recognize structured elements. miRISC binds single-stranded sequence motifs; therefore, secondary structures will have little effect on miRISC binding unless they occlude a site. We observed a ~1.5-fold higher enrichment of canonical binding sites at the 5′ end of the random sequence region for miRISC bearing let-7a, miR-34b, or miR-449a (Figures S1C and S1D).

Nevertheless, constant primer-binding sequences may bias the RBNS assay if they contain a motif for miRISC binding. In this case, miRISC may bind to virtually any RNA molecule in the pool, underestimating the enrichment of authentic binding motifs within the randomized sequence region, reducing RBNS sensitivity. miRNAs guide miRISC to their targets primary through Watson-Crick pairing with their "seed" sequence, miRNA nucleotide positions g2–g8 (Lewis et al., 2003, 2005; Rajewsky and Socci, 2004; Krek et al., 2005), which is displayed by AGO2 in a helical geometry ready to base pair (Parker et al., 2005; Wang et al., 2008; Elkayam et al., 2012; Nakanishi et al., 2012). Figure S1C presents the nomenclature for canonical site type classification. Given the high diversity and short length of miRNA seed sequences, the potential for miRISC binding to the constant regions is high. For example, constant regions in the RNA pool used by McGeary et al. (2019) contained at least one canonical site for 170 human miRNAs, including miR-7.

Even noncanonical, seed-matched sites with undetectable affinity may impact binding by interacting with higher affinity sites, e.g., those with slow k_{on}. miRISC binds rapidly to a short, seed-matched 4mer-m3.6 site, but this transient binding does not produce a measurable interaction. In contrast, 3′-only sites typically have slower association rate constants (~10^7 M^{-1} s^{-1}) than seed-matched sites (Salomon et al., 2015), because miRISC does not pre-organize the 3′ region of its miRNA guide. Yet a 3′-only 10mer-m10.19 site placed adjacent to a 4mer-m3.6 site present in the constant region of the RNA target displayed a diffusion-limited apparent k_{on} (Figure S1E). Constant regions cannot be readily modified to avoid biases, as they must remain compatible with Illumina sequencing. Inspired by Becker et al. (2019), we use cDNA oligonucleotides to block the common sequences present in each RNA molecule. Making these regions double-stranded prevents miRISC binding and disfavors intramolecular secondary structures, leaving only the randomized sequence and four 5′ and 3′ flanking nucleotides accessible (Figure 1). We benchmarked the blocker strategy using miR-449a miRISC, which has a low-affinity seed-matched binding site (5mer-A1) in the 3′ constant region of the RNA pool. DNA blocking oligonucleotides increased the enrichment of canonical sites 2.5- to 5-fold by decreasing the fraction of RNA with no binding site in the random sequence region (Figure S1F).

Time to reach equilibrium

K_D, the equilibrium dissociation constant, must be measured after binding reactions have reached equilibration. As reviewed by Jarmoskaite et al. (2020), binding follows an exponential curve characterized by its half-life, $t_{1/2}$. A conservative standard for equilibration is five half-lives, corresponding to 96.6% completion. Half-life is also linked to the equilibration rate constant k_{eq}:

$$k_{eq} = \frac{\ln 2}{t_{1/2}}.$$

Thus, the time for a binding reaction to reach 96.6% of completion is given by

$$T = 5 \times t_{1/2} = 5 \times \frac{\ln 2}{k_{eq}}.$$

For the binding equilibrium where miRISC interacts with an RNA $site_i$, the equilibration rate constant is described by

$$k_{eq} = k_{on} \times [\text{miRISC}] \times [\text{site}_i] + k_{off}.$$

The longer-lived the complex, the longer the incubation time required to reach equilibrium. For the let-7a 8mer site, $k_{on} = 2.4 \pm 0.1 \times 10^8$ $M^{-1}s^{-1}$, $k_{off} = 0.0036 \pm 0.0003$ s^{-1} (Salomon et al., 2015) and the concentration of the 8mer site in the RNA pool used for RBNS is 40 pM (Table S1). Under these conditions, the binding reaction with the lowest miRISC concentration (8 pM) should reach equilibrium in 16 ± 2 min. To provide sufficient time for equilibration, we incubated binding reactions for 2 h.

RNA pool concentration

In RBNS, the RNA pool concentration is the same for all binding reactions, while the RBP concentration varies. To measure K_D, the "titration" regime—in which the concentration of a binding site is much greater than K_D—must be avoided (reviewed by Jarmoskaite et al., 2020). We measured the frequency of known canonical and noncanonical binding sites in the RNA pool by high-throughput sequencing. Under our experimental conditions, concentrations of known binding sites are of the same order of magnitude as their K_D values. This is an acceptable regime, and K_D can be determined by an appropriate binding equation that explicitly accounts for bound protein and does not rely on the common assumption that $[\text{Protein}]_{free} \sim [\text{Protein}]_{total}$ (see STAR Methods).

Separation of bound and free RNA molecules

After reaching equilibrium, bound and free RNA are separated, e.g., by electrophoretic mobility shift assay, capture on beads coupled to specific antibodies that recognize the RBP or a ligand attached to the synthetic miRNA, and a double-filter binding assay.

The double-filter method has been used extensively to investigate the equilibrium binding and kinetic properties of protein-nucleic acid interactions (Wong and Lohman, 1992, 1993; Riggs et al., 1970a, 1970b; Winter and von Hippel, 1981; Clore et al., 1982; Wee et al., 2012; Xiao and MacRae, 2020). The nitrocellulose filter preferentially retains protein and protein-bound nucleic acids. The positively charged nylon filter placed directly beneath the nitrocellulose membrane traps protein-free nucleic acids not retained by the nitrocellulose.

RBNS using miRISC immobilized on paramagnetic beads provides an obvious route to high-throughput automation of the method, a prerequisite for defining the site types and their K_D values for all known mammalian miRNAs. We compared the double-filter binding assay with miRISC immobilized on

paramagnetic beads via an antibody-FLAG epitope tag interaction (Figures 1 and S2A). As the readout, we calculated the enrichment of canonical binding sites: the frequency of a binding site in miRISC-bound reads divided by its frequency in the starting pool (Figures 3B and S2B). Enrichment of canonical sites was 5- to 10-fold lower when bound RNA was recovered using bead-immobilized miRISC. Concomitantly, RNA non-specifically associated with miRISC was more abundant for the beads than in the double-filter binding assay, diluting the specifically recovered RNA and reducing RBNS sensitivity. We developed an alternative method for enrichment analysis to account for this higher background. Using this approach (described below) for the two methods yielded highly similar lists of binding sites (Figure S2C) with comparable affinities ($\leq$2-fold difference for 12 of 18 binding sites) (Figure S2D). While magnetic bead assays present a clear advantage for automation, the greater sensitivity and reduced handling of the double-filter binding assay is generally simpler, especially for kinetic assays.

De novo identification of binding sites

Principle

The number of occurrences of all *k*-mers of a specific length is counted over all the reads in a binding reaction. These counts are then divided by the total count of all *k*-mers to yield the *k*-mer frequency. The same procedure is repeated over the reads from the RNA input pool to account for any sequence biases present in the random region of RNA molecules. Finally, enrichment of a *k*-mer is defined as the frequency of the *k*-mer in protein-bound reads divided by its frequency in the RNA input pool (Figure 3C). A motif is identified as a binding site if (1) its enrichment is either above an arbitrarily defined value (McGeary et al., 2019) or is greater than a chosen *Z* score threshold (Lambert et al., 2014; Dominguez et al., 2018; Van Nostrand et al., 2020) and (2) it is not enriched in the no-RISC control reaction. Identified binding sites were also not enriched in a binding reaction containing the miRNA but not AGO2, indicating miRISC-specific binding (Figure S3A).

RBNS sensitivity depends on RBP concentration

Enrichment values are dependent on the concentration of RBP and produce a characteristic unimodal curve (Lambert et al., 2014). At low protein concentrations, enrichment of a motif increases with increasing protein concentration, as increasing amounts of bound RNA improve signal over a constant background of RNA molecules recovered even in the absence of protein. At high RBP concentrations, high-affinity motifs are saturated, and binding is driven toward lower affinity sites, resulting in a lower fraction of high-affinity motifs (Figure 3B). In our experiments using AGO2 miRISC, the relative rankings of binding motifs obtained at different protein concentrations were always preserved (Figures 3B, S1F, and S2B) and highly correlated (Spearman's $\rho > 0.83$ at adjacent protein concentrations), the binding reaction with the highest protein concentration offered the greatest sensitivity for low-affinity sites (Figure S3B). To limit sensitivity bias when comparing binding sites within various miRNAs, it is preferable to measure the active concentration of AGO2 and perform site discovery at similar active miRISC concentrations.

Sensitivity depends on the criteria chosen for significance

Motifs are typically considered enriched if their enrichment scores (McGeary et al., 2019) or *Z* scores (Lambert et al., 2014; Dominguez et al., 2018; Van Nostrand et al., 2020) exceed an arbitrarily defined threshold. Because binding site enrichment scores depend on the amount of RNA non-specifically recovered in the assay—increased background dilutes the specific signal, lowering enrichment scores—using a fixed threshold to compare different datasets may bias motif discovery. For example, a threshold of 10 (Figure S3C) (McGeary et al., 2019) applied to our AGO2 let-7a datasets generated by the double-filter binding method would yield a list of 17 enriched sites, but only 4 binding sites when using bead-immobilized miRISC (Figure S3D). This bias is prevented by using *Z* scores, which indicate the number of standard deviations by which enrichment values differ from the mean enrichment (Figure S2C). Previous reports used various values of *Z* score for different proteins and motif lengths (Lambert et al., 2014; Dominguez et al., 2018; Van Nostrand et al., 2020). To standardize the procedure of choosing a threshold, we consider a motif significantly enriched if its *Z* score $\geq$ 99.9 percentile (Figure S3E).

Finally, site discovery may be biased by "shadow motifs"—*k*-mers occurring in reads with high-affinity binding motifs but not conferring binding by themselves. An iterative procedure allows detection of "true binders" by masking reads harboring the most enriched binding site in the protein sample and in the RNA pool, then repeating the analysis iteratively (Figure 3C) (McGeary et al., 2019; Dominguez et al., 2018; Van Nostrand et al., 2020). All enrichments are recalculated on the masked reads to obtain the resulting most enriched motif, with this process continuing until the enrichment *Z* score (calculated from the original enrichment values) no longer meets the criterion for significance.

Motif size

Known miRISC binding sites are 5–12 nt long. As enrichment is calculated as the ratio of motif frequency in protein sample over RNA input pool, and division by zero is not supported, *k*-mers must be present in sequenced reads of the RNA pool. With current sequencing costs, one can readily sequence ~20 million reads for each condition. At this depth, ~200 reads of every possible 10mer will be present within the 20-nt-long randomized central region, but not all 11mers will be sampled. As sequencing costs fall, deeper sequencing should allow longer motif discovery.

Estimation of absolute K_D values using synthetic datasets

k-mer enrichment approximates relative binding affinities, because high-affinity binding sites are more enriched than lower affinity sites (Zykovich et al., 2009). However, enrichment depends on RBP concentration, saturation of high-affinity sites, and background binding (Figures 3B, S1F, and S2B). Consequently, enrichment (1) has an upper limit that depends on the length of both *k*-mers and the RNA molecules and (2) is not directly proportional to binding affinities (Lambert et al., 2015). Recently, a strategy was proposed to estimate relative K_D values solely from sequenced data without prior information on miRISC concentration, fraction bound of RNA molecules, and non-

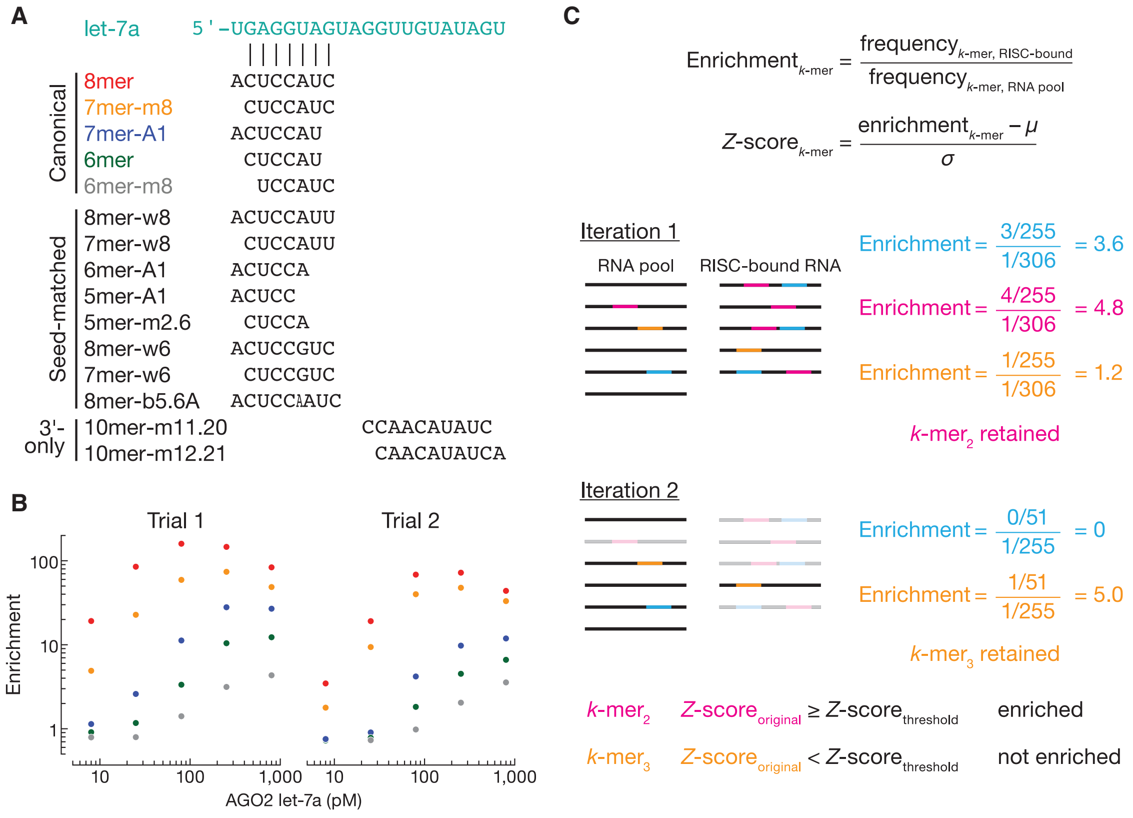

Figure 3. RBNS enables *de novo* discovery of binding sites

(A) Pairing of enriched sites.

(B) Enrichment profile of canonical let-7a sites observed at each of the five miRISC concentrations tested.

(C) Illustration of *de novo* site discovery strategy. Occurrences of all *k*-mers are calculated in all the sequencing reads (black lines). Each λ-nt-long read contains $\lambda - k + 1$ motifs. For example, the magenta 10mer appears four times in the miRISC-bound sequencing data (five reads total), therefore its frequency in this sample is $4 \div (5 \times 55)$. The most enriched motif is selected, and its *Z* score is compared with the *Z* score threshold. The magenta motif is the most enriched, and its *Z* score is above the *Z* score threshold; therefore, this motif is retained. Next, all reads containing the identified motif are masked in the miRISC-bound and RNA pool sequencing data (gray lines). All enrichment values are recalculated on masked reads to eliminate "shadow" motifs (blue). The orange motif is the most enriched at this iteration and is retained. Nevertheless, its *Z* score (calculated from the original enrichment values) is below the *Z* score threshold; therefore, the orange *k*-mer is not considered as enriched. This iterative procedure continues until the *Z* score of the most enriched binding site is below the *Z* score threshold.

specific RNA recovered as background (McGeary et al., 2019). We modified this procedure to simultaneously determine *absolute* K_D values using maximum likelihood estimation (MLE) (Figure 4A). In this statistical model, the parameter values—the K_D values of various sites, miRISC concentration, and the background of RNA interacting non-specifically with the beads, membrane, or tubes—of a mathematical model predicting expected sequencing counts are fit to maximize the likelihood of observing the predicted sequencing counts in experimental data. We tested our approach using simulated RBNS datasets containing four binding sites and corresponding to our typical experimental conditions. Figure S4A shows the convergence of a typical MLE fit. Our method accurately estimated the ground truth affinity values of the four binding sites (Figures 4B and S4B).

Estimation of absolute K_D values using experimental datasets

Next, we applied our MLE procedure to experimental let-7a datasets. To test whether the method's K_D estimates were robust, we removed one sample from the dataset and re-fitted the parameters using the remaining binding reactions. Estimates obtained by this leave-one-out procedure strongly correlated in pairwise comparisons (Pearson's r $\geq$ 0.969 for each of the 15 pairwise comparisons), even when we removed the highest miRISC concentration or the no-RISC binding reaction (Figures 4C and S4C). Estimates were also highly reproducible in two independent experiments, each comprising five protein concentrations and no-RISC control (Pearson's r $\geq$ 0.974; Figure 4D).

To estimate K_D values and their 95% confidence intervals (CI), we first performed fitting optimization 2,000 times. Each binding experiment used two independent trials to account for errors caused by sample-to-sample variation. Second, we bootstrapped sequencing data 10 times to account for the error caused by the multinomial down-sampling of RNA molecules during sequencing. Third, each fitting optimization was performed 100 times using independent and partially randomized starting points to account for the error of ending at a local minimum. Importantly, while values for the initial guess spanned a >20-fold

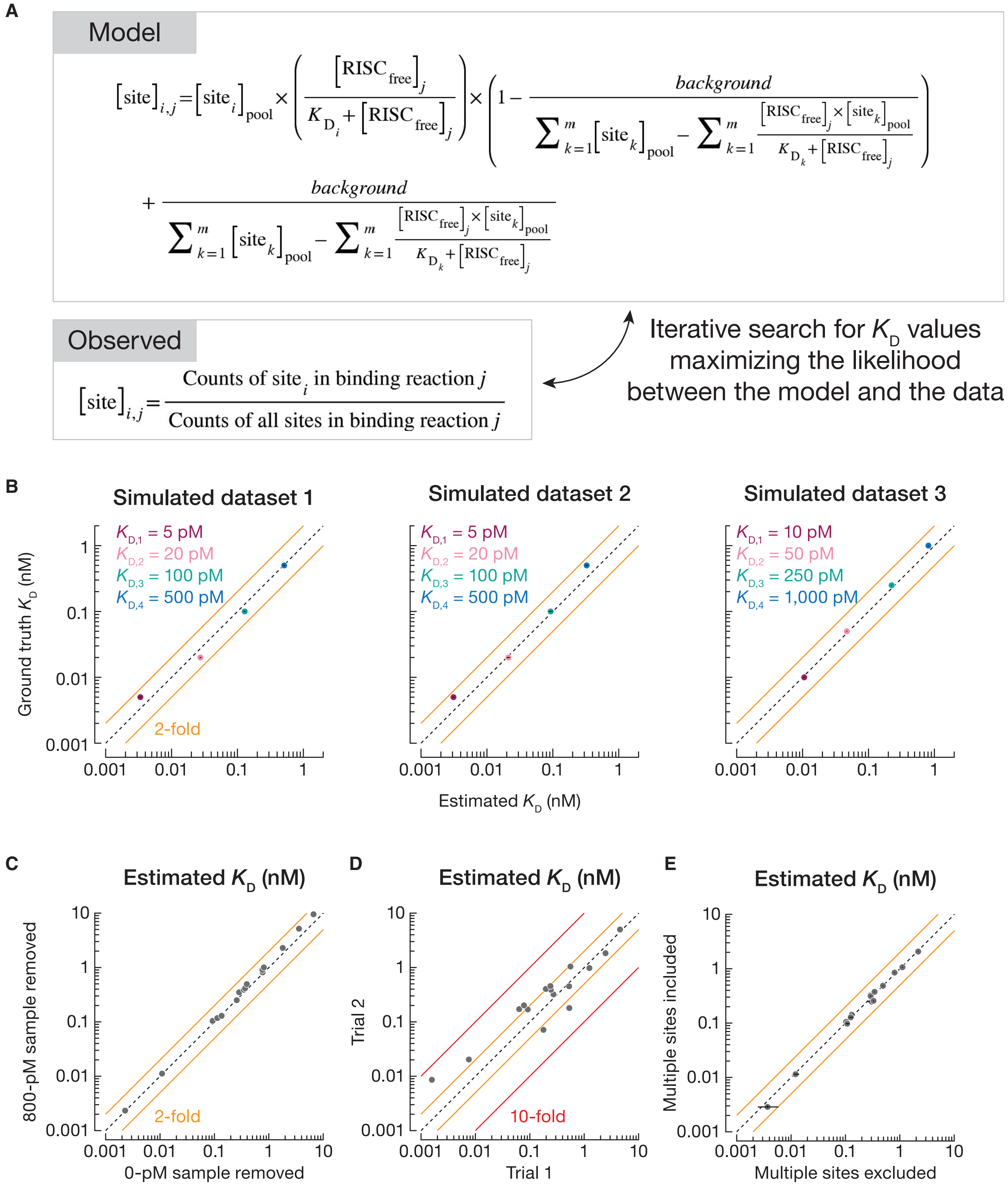

Figure 4. Estimation of K_D values by RBNS is robust and reproducible

(A) The mathematical equation derived from the biochemical model at equilibrium describes predicted concentration of binding site *i* in sequencing data, given K_D values of all binding sites, miRISC concentration, and background. These parameters are fit simultaneously to maximize the likelihood of observing predicted sequencing counts in experimental data.

(legend continued on next page)

range, fitted parameters were robustly estimated, yielding narrow 95% CI (Figures 5, 6, and S2D).

We note that our procedure for estimating K_D values uses only reads containing one miRNA-binding site. Reads containing multiple instances of binding sites represented $\leq 1\%$ of sequence reads in the starting library. Omission of these reads did not affect estimation of K_D values: fractionally assigning such multi-site reads to appropriate binding sites yielded the same results as excluding them (Figure 4E).

Fitted parameters were also insensitive to the number and identity of binding sites. For example, when optimization was performed with 15 statistically enriched site types (Figure 5A) our analysis for let-7a sites estimated K_D^{8mer} = 4 pM (95% CI = [2, 6]), $K_D^{7mer-A1}$ = 125 pM (95% CI = [116, 140]), and $K_D^{6mer-A1}$ = 490 pM (95% CI = [484, 497]). Using 12 seed-matched site types with different t1 nucleotides (Figure S4D) gave essentially the same results: K_D^{8mer} = 4 pM (95% CI = [4, 6]), $K_D^{7mer-A1}$ = 135 pM (95% CI = [125, 146]), and $K_D^{6mer-A1}$ = 524 pM (95% CI = [516, 535]). Our results agreed well with K_D values measured by ensemble biochemistry and single-molecule approaches (Figures 5A, S4E, and S4F) (Wee et al., 2012; Salomon et al., 2015; Becker et al., 2019; Sheu-Gruttadauria et al., 2019) and with the relative binding affinities measured by McGeary et al. (2019) (Figure 5B).

High-throughput absolute K_D estimation provides insights into miRNA targeting

Our analytical approach can estimate the affinity of any binding site of interest—enriched or not—provided the motif is represented in the sequenced data. We used our strategy to compute the K_D values for *de novo* identified binding sites from previously published RBNS datasets (McGeary et al., 2019) and from additional datasets generated in this study (Figures 5B, S5A, and S6). We found that canonical binding sites display miRNA-specific differences in their affinities. For example, the *C. elegans* miRNA, lsy-6, loaded into human AGO2 had a 5-fold weaker affinity for an 8mer than AGO2:miR-1 miRISC (Figure S5A). Notably, the seed of miR-1 is predicted by nearest neighbor methods (Turner and Mathews, 2010) to pair more strongly with its target than the seed of the lsy-6 miRNA (Figure S5B), consistent with the known relationship between the predicted strength of seed-pairing and the efficacy of target mRNA repression (Garcia et al., 2011). While affinity increased with increased predicted pairing stability, the correlation between measured affinity and affinity predicted by nearest neighbor free energy was significant only for 7mer-m8 binding sites (Figure S5B). Affinity of seed-matched sites was also increased by A at position t1 (Figure 6A), which preferentially interact with a t1-nucleotide-binding pocket in AGO2 (Schirle et al., 2015).

Importantly, miRNAs bound some noncanonical sites with greater affinity than a canonical 6mer site (Figures 5B, S5A, and S6). These included 3′-only sites that extensively pair to the 3′ region of the miRNA without pairing to the seed. These sites were identified in the let-7a, lsy-6, miR-155, and miR-124 datasets using the *Z* score approach and display as much as 4-fold greater binding affinity than that of the canonical 6mer site (Table S3). By contrast, miR-1, miR-7, miR-449a, and miR-34b bound 3′-only sites poorly (K_D > 1.2 nM). We note that the seed regions of these miRNAs are predicted by nearest neighbor analysis to pair more strongly and their 3′ regions more weakly than the equivalent sites for let-7a, lsy-6, miR-155, and miR-124, likely explaining the pronounced miRNA-specific differences in the repertoire of noncanonical sites.

Single target mismatches, insertions, and deletions disrupting the seed detectably increased K_D (Figures 6B–6D) (Wee et al., 2012; Becker et al., 2019; Sheu-Gruttadauria et al., 2019). Surprisingly, some sites bearing a central 1-nt bulge in the seed were detected among enriched motifs (Figures 3A, 5B, S5A, and S6), suggesting that these bulges are better tolerated. For example, insertion of guanosine between positions 5 and 6 (b5.6G) in miR-124 seed-matched sites decreased affinity by only 21-fold (Figure 6D). This binding site corresponds to a nucleation-bulge site and was previously identified by high-throughput crosslinking and immunoprecipitation (Chi et al., 2012). Nevertheless, 67% of enriched sites (8 of 12) with one target nucleotide inserted in the center of the seed do not use this mode of binding, suggesting that bulged sites may be more common than previously appreciated, but are not used by all miRNAs.

Determining relative kinetic parameters by RBNS using synthetic datasets

Kinetic studies of molecular interactions provide powerful insights into the underlying microscopic mechanisms and functional outcome. We reasoned that RBNS might enable high-throughput measurement of the kinetic behavior of an RBP if the bound RNA was recovered at different times after the binding reaction was started (Figure S7A). Traditional association kinetics experiments monitor the time-dependent progress of RNA binding at different protein concentrations. To simplify the experiment, one usually uses pseudo first-order conditions, with a low concentration of one reactant (e.g., RNA) and $\geq$10-fold higher concentration of the second reactant (e.g., RBP). Under these conditions, the observed rate constant k_{obs} is related to RBP concentration and the association and dissociation rate constants as $k_{obs} = k_{on} \times [RBP] + k_{off}$. A plot of k_{obs} versus [RBP] is linear, with slope k_{on} and y intercept k_{off}. Therefore, association kinetics requires performing a time series with several protein concentrations. An alternative approach

(B) Testing K_D estimation with simulated data. RBNS data were modeled by simulating miRISC binding to RNA pool containing four binding sites and no-site molecules. Stock concentration of miRISC was equal to 2.1 nM (dataset 1) or 8.1 nM (datasets 2 and 3). Background was set to 0.1 nM and $K_{Dnosite}$ was set to 5 nM. Error bars indicate 95% CI on the median.
(C) Comparison of sub-datasets when the highest miRISC concentration and no-RISC binding reaction were removed (Pearson's r = 0.979). Binding sites identified in Figure 3 were used to compute K_D.
(D) Correspondence between fitted K_D values of enriched binding sites estimated from two independent binding experiments (Pearson's r = 0.974). The solid orange and red lines indicate 2- and 10-fold differences, respectively. Dashed diagonal lines show $y = x$.
(E) Comparison of fitted KD values when multi-site reads were fractionally assigned to corresponding site types or excluded from the analysis.

CellPress
OPEN ACCESS

A

	K_D (pM)			
Site type	RBNS [95%CI]	Published Value	Study	Method
8mer	4 [2, 6]	15 ± 2	Salomon et al.	CoSMoS
		27	Becker et al.	High-throughput imaging
7mer-m8	12 [11, 14]	26 ± 2	Wee et al.	Double filter binding
		42 ± 2	Sheu-Gruttadauria et al.	Double filter binding
7mer-A1	125 [116, 140]	480 ± 60	Salomon et al.	CoSMoS
6mer	343 [342, 345]	400 ± 2	This study	CoSMoS
6mer-A1	490 [484, 497]	1002 ± 200	Salomon et al.	CoSMoS
		2200 ± 30	This study	CoSMoS
6mer-m8	800 [797, 802]	2100 ± 40	This study	CoSMoS

B

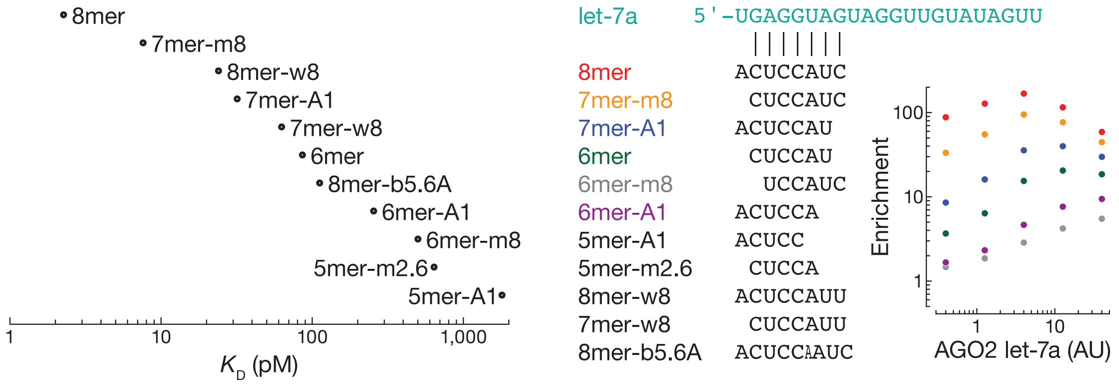

Figure 5. K_D values estimated by RBNS agree with previous results

(A) Comparison of K_D values estimated by RBNS and measured by ensemble biochemistry and single-molecule approaches.

(B) K_D values fitted for AGO2 let-7a RBNS from McGeary et al. (2019). Error bars indicate 95% CI on the median. Center: pairing of enriched sites identified by *de novo* site discovery. Right: enrichment profile of canonical let-7a sites.

measures binding at different time points under pseudo first-order kinetics, while blocking reassociation by dilution or adding a high-affinity RNA competitor target at high concentration. In this experimental setup, a single protein concentration is used; k_{off} is first determined from the dissociation and is then used to infer k_{on} from k_{obs}.

We developed a computational strategy that does not assume pseudo first-order kinetics and simultaneously fits association and dissociation rate constants using the same RBNS dataset (Figure 7A). Our mathematical model predicts the read counts for each site type across the time series as a function of k_{on} and k_{off} values for each miRNA site type (including "no-site"). We also estimated a constant amount of RNA molecules recovered as background in all samples.

To test our approach, we used simulated kinetic RBNS datasets, in which the observations were modeled to match our typical experimental conditions (Figure S7B). Our analytical method did not recover the absolute ground truth parameters (Figure S7B). The cost function f_{cost}, which we minimize during the optimization procedure, is a linear combination of exponential functions. This function has multiple minima, and f_{cost} was routinely trapped at a local minimum yielding k_{on} and k_{off} values different from the ground truth (Figure 7B). Nevertheless, our method was able to discriminate between fast and slow binders—but maybe was not able to distinguish rank order within these broad classes of sites—and preserved the relative differences in kinetic parameters among binding sites (Figure 7C).

Estimation of relative kinetic parameters using experimental datasets

To benchmark our approach, we used AGO2 let-7a miRISC, whose kinetics have been measured previously (Figures S5D and S5E) (Becker et al., 2019; Salomon et al., 2015). Leave-one-out validation showed that the fitted k_{on} and k_{off} parameters were robustly estimated (Pearson's r $\geq$ 0.995 for each of the 55 pairwise comparisons; Table S4). We note that estimation of k_{off} for long-lived binding events was sensitive to removing the first and the equilibrium time points from the kinetic series (Figure 7D). Estimates differed by $\leq$2-fold between two independent experiments using two different miRISC preparations (Pearson's r = 0.86) with two noteworthy exceptions (Figure 7E). The k_{on} of 5mer-m8 and the k_{off} of 11mer-m11.21 differed between experiments by 5- and 3.5-fold, respectively. These observations highlight two pitfalls of RBNS that we also observed measuring binding affinities: estimation is less accurate for (1)

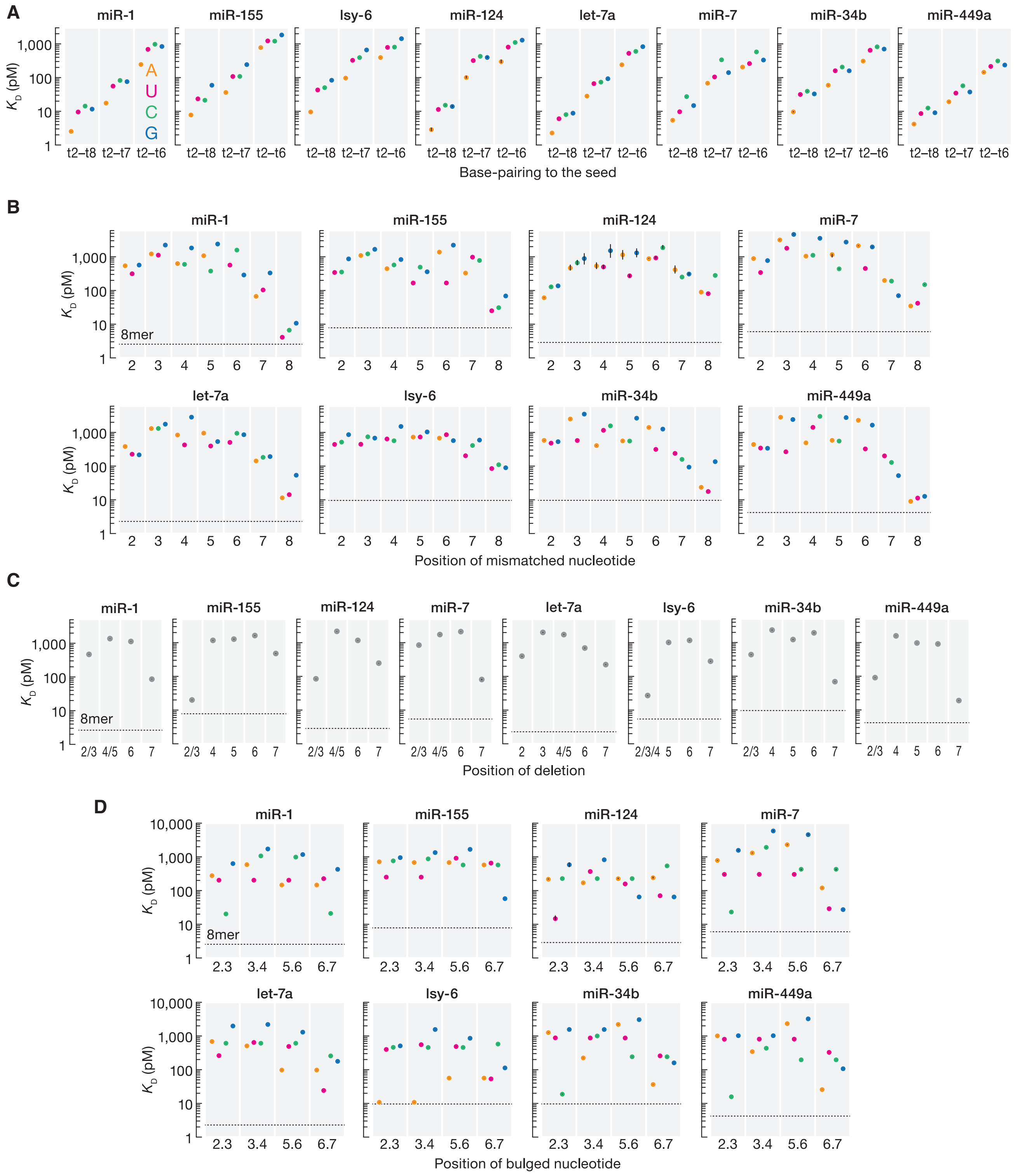

Figure 6. RBNS provides insight into miRNA targeting

(A) K_D values for t2–t8, t2–t7, and t2–t6 targets with different t1 nucleotides.

(B–D) K_D values for t2–t8 targets with t1A and different one-nucleotide mismatches (B), deletions (C), and bulges (D) at indicated positions. We note that deletion of *N* nt and *N*+1 nt may yield the same sequence. Because we cannot discriminate between these two sites, they are annotated as "*N*/*N*+1".

Error bars indicate 95% CI on the median. Horizontal dashed line indicates K_D^{8mer}. Adenine (orange), uridine (magenta), cytidine (cyan), and guanosine (blue).

CellPress
OPEN ACCESS

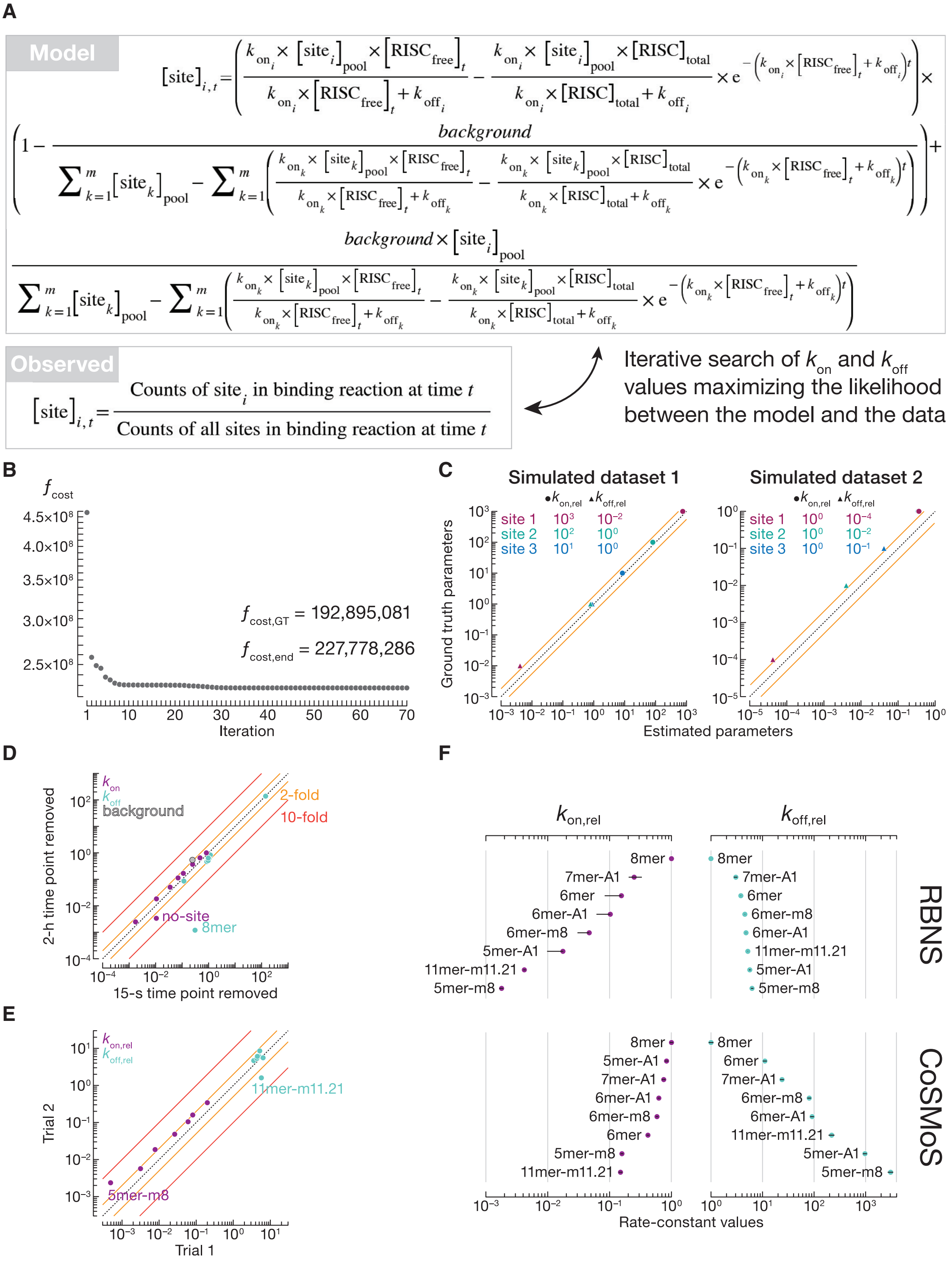

Figure 7. Estimation of k_{on} and k_{off} values by RBNS

(A) The mathematical equation derived from the biochemical model describes the predicted concentration of binding site *i* at a time point *t* in the sequencing data, given k_{on} and k_{off} values of all binding sites, and background. These parameters are fit simultaneously to maximize the likelihood of observing predicted sequencing counts in experimental data.

(B and C) Testing k_{on} and k_{off} estimation with simulated data. RBNS data were modeled by simulating miRISC binding to RNA pool containing four binding sites and no-site molecules. Dataset 1 contains binding sites with similar k_{off} but different k_{on} values. Dataset 2 contains binding sites with similar k_{on} but different k_{off}

(legend continued on next page)

longer and therefore more sparse motifs, and (2) for no-site motifs. (The 5mer-m8 site type was not enriched in our RBNS equilibrium data but was included in the kinetic analysis for comparison with single-molecule results.) Importantly, our mathematical model and fitting algorithm successfully discriminated between fast—the canonical seed-matched 8mer, 7mer-m8, 7mer-A1, and 6mer sites—and slow binders—the 3′-only site 11mer-m11.21 and the 5mer-m8 (Figure 7F). The same hierarchy of fast and slow binding sites was observed by single-molecule assays, albeit the dynamic range of k_{on} was compressed relative to RBNS. This variance reflects technical differences between the two methods. First, single-molecule approaches use one unique RNA target >20 nt long, whereas RBNS measures binding for sites $\leq$12 nt, because longer motifs are too infrequent in the sequencing data. Second, the identity of nucleotides flanking binding sites impacts k_{on} *in vitro*, with different flanking contexts spanning as much as a 100-fold range (Figure S7C) (Becker et al., 2019). RBNS averages these differences while single-molecule assays are typically performed using the most favorable context.

Our approach readily distinguished between long-lived and short-lived interactions: canonical seed-matched sites had the slowest departure rates (small k_{off}), whereas the 11mer-m11.21 and 5mer-m8 sites displayed the fastest (Figure 7F). Although consistent with single-molecule measurements, the dynamic range of k_{off} values estimated by RBNS was narrower than that offered by single-molecule assays. We do not currently understand the source of the compression of dynamic range in k_{off}, but it is unlikely to arise from technical issues in the MLE procedure, because our method preserved the relative relationship among k_{off} values with simulated data (Figure 7C). If sufficient protein sample is available, we suggest performing both equilibrium and kinetic series to obtain K_D and k_{on} and use these values to infer k_{off}.

DISCUSSION

RBNS is a straightforward and cost-effective strategy to interrogate the sequence specificities of RBPs. RBNS is often characterized as an *unbiased* method for site discovery *in vitro*. The method does enable simultaneous binding of RBPs to an exhaustive list of motifs. But as discussed above, several factors in the experimental design and computational analysis can alter the outcome of the assay. Nevertheless, when designed and analyzed with potential biases in mind, RBNS provides a high-throughput route to assessing the biochemical and kinetic properties of RBPs.

Despite its greater utility, RBNS has mainly been used to obtain a list of binding motifs (Taliaferro et al., 2016; Dominguez et al., 2018; Hale et al., 2018; Van Nostrand et al., 2020). Notably, some studies have employed RBNS to obtain qualitative, relative binding affinities (Lambert et al., 2014; McGeary et al., 2019). Here, we present a computational method that estimates the *absolute* values of dissociation constants for an RBP of interest without prior knowledge of miRISC concentration, fraction of RNA molecules bound, or non-specific RNA recovered as background. Our procedure uses sequencing reads containing single sites or a no-site. Because the proportion of reads with multiple sites is low ($\leq$1%), we did not consider a more complex model considering all states in which the AGO-miRNA complex is bound to target sites. Importantly, estimating absolute—rather than relative—affinities does not require considering non-specific binding to no-site RNAs, which display more variance in affinity estimates than sequence-specific binding sites.

Applying our approach to simulated RBNS datasets showed that the method accurately estimated the ground truth K_D values of the binding sites. Our ability to measure absolute K_D values enables direct comparison of binding affinities for the same regulatory site type among different miRNAs. For example, our analysis revealed a 5-fold range in binding affinities for 8mers of human miR-1 and worm lsy-6. If miR-1 and lsy-6 were present in the same concentrations in a cell and the distribution of their 8mer sites was the same for the two, one would observe a smaller perturbation of gene expression for a loss of lsy-6 function than that of miR-1.

We benchmarked our strategy using the well-studied AGO2:let-7a miRISC and obtained robust results in good agreement with previous biochemical and single-molecule measurements (Becker et al., 2019; Salomon et al., 2015; Sheu-Gruttadauria et al., 2019; Wee et al., 2012; Schirle et al., 2015). As affinities for all motifs are assessed simultaneously, RBNS combined with our computational methods may become an alternative to traditional low-throughput quantitative methods. For example, our analysis of published and newly generated AGO2 datasets provides a resource for replacing >450 binding assays. Our estimation of dissociation constants for canonical and noncanonical sites for eight different miRNAs gives further support to binding models established using one or two miRNA sequences (Becker et al., 2019; Salomon et al., 2015; Sheu-Gruttadauria et al., 2019; Wee et al., 2012; Schirle et al., 2015).

We envision that absolute K_D values measured using RBNS may enable prediction of changes in the occupancy of miRNA-guided AGO2 or other RBPs at sites across the transcriptome

values. (B) Convergence of a representative MLE fit. The cost function f_{cost} is minimized to a final value $f_{cost,end}$. $f_{cost,\ GT}$: f_{cost} calculated with ground truth parameters. (C) Fitted k_{on} and k_{off} values are reported relative to those of site 4.
(D) Comparison of sub-datasets when the shortest and the longest times were removed (Pearson's r = 0.998). Indicated are the site types showing >2-fold difference. Fitted parameters include k_{on} ($nM^{-1}\ s^{-1}$), k_{off} (s^{-1}), and background (nM).
(E) Correspondence between fitted k_{on} and k_{off} values estimated from two independent binding experiments (Pearson's r = 0.86). Kinetic parameters are reported relative to those of 8mer site. Indicated are the site types showing >4-fold difference.
(F) Comparison of k_{on} and k_{off} values estimated by RBNS and measured by co-localization single-molecule spectroscopy (CoSMoS). CoSMoS data from Figure S5D and (Salomon et al., 2015). Error bars indicate 95% CI on the median (RBNS) or propagated error on the mean (CoSMoS). RNA molecules used to measure binding of 5mer-A1 by CoSMoS unintentionally contained an additional 5mer-m2.6-w2 binding site. The solid orange and red lines indicate 2- and 10-fold difference, respectively. Dashed diagonal lines show $y = x$.

in response to developmental and external stimuli, thereby allowing the modeling of the resulting changes in regulatory activity. For miRNAs, RBNS using miRISC immobilized on paramagnetic beads promises to allow determination of the site types and equilibrium binding affinities for all mammalian miRNAs, a prerequisite for predicting the occupancy and identity of miRNA target sites *in vivo*. Finally, k_{on} and k_{off} measured by RBNS may prove useful for understanding the mechanisms of molecular interactions, providing additional information for developing quantitative models of biology.

Limitations of the study

The method presented here has broad utility in quantitatively assessing the specificity of RNA- or DNA-binding proteins for nucleic acids. We consider miRISC in solution with the RNA pool, and we assume that miRISC may bind one of the *k*-mers within the RNA molecule with 1:1 stoichiometry and a Hill coefficient of 1 and recovery of bound RNA is complete. For K_D estimation, we also assume that the reaction has reached equilibrium, and concentrations of binding sites are below or of the same order of magnitude as their K_D values. *De novo* identification of binding sites, and reliable measurement of K_D, k_{on}, and k_{off} values of motifs of interest are achieved if these sites are represented in the sequenced reads. For RNA molecules with 20-nt random region and datasets consisting of 10–20 million sequenced reads after filtering steps, the maximum effective motif size for *de novo* site discovery is 10 nt. Interrogation of longer motifs could be achieved by including a constant region, such as an imperfect seed sequence, in addition to a random region (McGeary et al., 2022). For fitting K_D, k_{on}, and k_{off} values, longer motifs can be interrogated, as the sequenced data are not required to contain *all* *k*-mers of a certain length. While our approach correctly ranked long-lived and short-lived interactions, fitted k_{off} values may underestimate differences among the binding motifs. Therefore, we suggest fitting K_D and k_{on} and using these values to infer k_{off}.

STAR★METHODS

Detailed methods are provided in the online version of this paper and include the following:

- KEY RESOURCES TABLE
- RESOURCE AVAILABILITY
  - Lead contact
  - Materials availability
  - Data and code availability
- EXPERIMENTAL MODEL AND SUBJECT DETAILS
  - Cell lines
- METHOD DETAILS
  - RISC purification
  - Northern blotting
  - Quantification of active miRISC by double-filter binding assay
  - Co-localization single-molecule spectroscopy
  - RNA bind-*n*-Seq for de novo site discovery and K_D measurements
  - RNA bind-*n*-Seq using paramagnetic beads
  - RNA bind-*n*-Seq for kinetic measurements
- QUANTIFICATION AND STATISTICAL ANALYSIS
  - Quantification of active miRISC by double-filter binding assay
  - Co-localization single-molecule spectroscopy
  - Quality control of high-throughput sequencing data
  - Enrichment values
  - De novo site discovery
  - Read assignments
  - Modeling of RBNS experiments
  - Maximum likelihood estimation (MLE) of parameters from RBNS data
  - Cost function for parameter fitting
  - Implementation of MLE
  - Mathematical model for MLE of K_D values
  - Parameters fitted during MLE of K_D values
  - Derivation of f_{cost} for MLE of K_D values
  - Initial guess for MLE of K_D values and calculation of 95% CIs
  - Mathematical model for MLE of k_{on} and k_{off} values
  - Parameters fitted during MLE of k_{on} and k_{off} values
  - Derivation of f_{cost} for MLE of k_{on} and k_{off} values
  - Initial guess for MLE of k_{on} and k_{off} values and calculation of 95% CIs

SUPPLEMENTAL INFORMATION

Supplemental information can be found online at https://doi.org/10.1016/j.crmeth.2022.100185.

ACKNOWLEDGMENTS

We thank members of the Zamore laboratory for critical comments on the manuscript, Ildar Gainetdinov for advice on designing the RNA library, Amena Arif for gift of pScalps Puro EGFP plasmid, and Ken-Edwin Aryee for providing psPAX2 and pMD2.G plasmids. This work was supported in part by National Institutes of Health grant R35 GM136275 to P.D.Z. K.J. was supported by a Charles A. King Trust Postdoctoral Fellowship.

AUTHOR CONTRIBUTIONS

Conceptualization, K.J., J.V.-B., and P.D.Z.; methodology, K.J. and J.V.-B.; investigation, K.J. and J.V.-B.; formal analysis, K.J.; software, K.J.; writing – original draft, K.J.; writing – review & editing, K.J. and P.D.Z.; supervision, P.D.Z.; funding acquisition, P.D.Z.

DECLARATION OF INTERESTS

The authors declare no competing interests.

Received: September 27, 2021
Revised: January 18, 2022
Accepted: February 25, 2022
Published: March 18, 2022

REFERENCES

Aitken, C.E., Marshall, R.A., and Puglisi, J.D. (2008). An oxygen scavenging system for improvement of dye stability in single-molecule fluorescence experiments. Biophys. J. *94*, 1826–1835.

Baek, D., Villén, J., Shin, C., Camargo, F.D., Gygi, S.P., and Bartel, D.P. (2008). The impact of microRNAs on protein output. Nature *455*, 64–71.

Bazzini, A.A., Lee, M.T., and Giraldez, A.J. (2012). Ribosome profiling shows that miR-430 reduces translation before causing mRNA decay in zebrafish. Science *336*, 233–237.

Becker, W.R., Ober-Reynolds, B., Jouravleva, K., Jolly, S.M., Zamore, P.D., and Greenleaf, W.J. (2019). High-throughput analysis reveals rules for target RNA binding and cleavage by AGO2. Mol. Cell *75*, 741–755.e11.

Buenrostro, J.D., Araya, C.L., Chircus, L.M., Layton, C.J., Chang, H.Y., Snyder, M.P., and Greenleaf, W.J. (2014). Quantitative analysis of RNA-protein interactions on a massively parallel array reveals biophysical and evolutionary landscapes. Nat. Biotechnol. *32*, 562–568.

Campbell, Z.T., and Wickens, M. (2015). Probing RNA-protein networks: biochemistry meets genomics. Trends Biochem. Sci. *40*, 157–164.

Chi, S.W., Hannon, G.J., and Darnell, R.B. (2012). An alternative mode of microRNA target recognition. Nat. Struct. Mol. Biol. *19*, 321–327.

Clore, G.M., Gronenborn, A.M., and Davies, R.W. (1982). Theoretical aspects of specific and non-specific equilibrium binding of proteins to DNA as studied by the nitrocellulose filter binding assay. Co-operative and non-co-operative binding to a one-dimensional lattice. J. Mol. Biol. *155*, 447–466.

Cook, K.B., Vembu, S., Ha, K.C.H., Zheng, H., Laverty, K.U., Hughes, T.R., Ray, D., and Morris, Q.D. (2017). RNAcompete-S: combined RNA sequence/structure preferences for RNA binding proteins derived from a single-step in vitro selection. Methods *126*, 18–28.

Crawford, D.J., Hoskins, A.A., Friedman, L.J., Gelles, J., and Moore, M.J. (2008). Visualizing the splicing of single pre-mRNA molecules in whole cell extract. RNA *14*, 170–179.

Danan, C., Manickavel, S., and Hafner, M. (2016). PAR-CLIP: a method for transcriptome-wide identification of RNA binding protein interaction sites. Methods Mol. Biol. *1358*, 153–173.

Dave, R., Terry, D.S., Munro, J.B., and Blanchard, S.C. (2009). Mitigating unwanted photophysical processes for improved single-molecule fluorescence imaging. Biophys. J. *96*, 2371–2381.

Dignam, J.D., Lebovitz, R.M., and Roeder, R.G. (1983). Accurate transcription initiation by RNA polymerase II in a soluble extract from isolated mammalian nuclei. Nucleic Acids Res. *11*, 1475–1489.

Doench, J.G., and Sharp, P.A. (2004). Specificity of microRNA target selection in translational repression. Genes Dev. *18*, 504–511.

Dominguez, D., Freese, P., Alexis, M.S., Su, A., Hochman, M., Palden, T., Bazile, C., Lambert, N.J., Van Nostrand, E.L., Pratt, G.A., et al. (2018). Sequence, structure, and context preferences of human RNA binding proteins. Mol. Cell *70*, 854–867.e9.

Elkayam, E., Kuhn, C.D., Tocilj, A., Haase, A.D., Greene, E.M., Hannon, G.J., and Joshua-Tor, L. (2012). The structure of human argonaute-2 in complex with miR-20a. Cell *150*, 100–110.

Erickson, M.G., Alseikhan, B.A., Peterson, B.Z., and Yue, D.T. (2001). Preassociation of calmodulin with voltage-gated Ca(2+) channels revealed by FRET in single living cells. Neuron *31*, 973–985.

Erickson, M.G., Liang, H., Mori, M.X., and Yue, D.T. (2003). FRET two-hybrid mapping reveals function and location of L-type Ca2+ channel CaM preassociation. Neuron *39*, 97–107.

Flores-Jasso, C.F., Salomon, W.E., and Zamore, P.D. (2013). Rapid and specific purification of Argonaute-small RNA complexes from crude cell lysates. RNA *19*, 271–279.

Frey, K.A., and Albin, R.L. (2001). Receptor binding techniques. Curr. Protoc. Neurosci. *1*, 1.4.

Garcia, D.M., Baek, D., Shin, C., Bell, G.W., Grimson, A., and Bartel, D.P. (2011). Weak seed-pairing stability and high target-site abundance decrease the proficiency of lsy-6 and other microRNAs. Nat. Struct. Mol. Biol. *18*, 1139–1146.

Garland, P.B. (1996). Optical evanescent wave methods for the study of biomolecular interactions. Q. Rev. Biophys. *29*, 91–117.

Guo, H., Ingolia, N.T., Weissman, J.S., and Bartel, D.P. (2010). Mammalian microRNAs predominantly act to decrease target mRNA levels. Nature *466*, 835–840.

Hale, M.A., Richardson, J.I., Day, R.C., McConnell, O.L., Arboleda, J., Wang, E.T., and Berglund, J.A. (2018). An engineered RNA binding protein with improved splicing regulation. Nucleic Acids Res. *46*, 3152–3168.

Harris, C.R., Millman, K.J., van der Walt, S.J., Gommers, R., Virtanen, P., Cournapeau, D., Wieser, E., Taylor, J., Berg, S., Smith, N.J., et al. (2020). Array programming with NumPy. Nature *585*, 357–362.

Hellman, L.M., and Fried, M.G. (2007). Electrophoretic mobility shift assay (EMSA) for detecting protein-nucleic acid interactions. Nat. Protoc. *2*, 1849–1861.

Hendrickson, D.G., Hogan, D.J., McCullough, H.L., Myers, J.W., Herschlag, D., Ferrell, J.E., and Brown, P.O. (2009). Concordant regulation of translation and mRNA abundance for hundreds of targets of a human microRNA. PLoS Biol. *7*, e1000238.

Huntzinger, E., and Izaurralde, E. (2011). Gene silencing by microRNAs: contributions of translational repression and mRNA decay. Nat. Rev. Genet. *12*, 99–110.

Jarmoskaite, I., AlSadhan, I., Vaidyanathan, P.P., and Herschlag, D. (2020). How to measure and evaluate binding affinities. Elife *9*, e57264.

Kishore, S., Jaskiewicz, L., Burger, L., Hausser, J., Khorshid, M., and Zavolan, M. (2011). A quantitative analysis of CLIP methods for identifying binding sites of RNA-binding proteins. Nat. Methods *8*, 559–564.

König, J., Zarnack, K., Rot, G., Curk, T., Kayikci, M., Zupan, B., Turner, D.J., Luscombe, N.M., and Ule, J. (2010). iCLIP reveals the function of hnRNP particles in splicing at individual nucleotide resolution. Nat. Struct. Mol. Biol. *17*, 909–915.

Krek, A., Grün, D., Poy, M.N., Wolf, R., Rosenberg, L., Epstein, E.J., MacMenamin, P., da Piedade, I., Gunsalus, K.C., Stoffel, M., et al. (2005). Combinatorial microRNA target predictions. Nat. Genet. *37*, 495–500.

Ladbury, J.E. (1995). Counting the calories to stay in the groove. Structure *3*, 635–639.

Ladbury, J.E., and Chowdhry, B.Z. (1996). Sensing the heat: the application of isothermal titration calorimetry to thermodynamic studies of biomolecular interactions. Chem. Biol. *3*, 791–801.

Lambert, N., Robertson, A., Jangi, M., McGeary, S., Sharp, P.A., and Burge, C.B. (2014). RNA Bind-n-Seq: quantitative assessment of the sequence and structural binding specificity of RNA binding proteins. Mol. Cell *54*, 887–900.

Lambert, N.J., Robertson, A.D., and Burge, C.B. (2015). RNA bind-n-seq: measuring the binding affinity landscape of RNA-binding proteins. Methods Enzymol. *558*, 465–493.

Lewis, B.P., Shih, I.H., Jones-Rhoades, M.W., Bartel, D.P., and Burge, C.B. (2003). Prediction of mammalian microRNA targets. Cell *115*, 787–798.

Lewis, B.P., Burge, C.B., and Bartel, D.P. (2005). Conserved seed pairing, often flanked by adenosines, indicates that thousands of human genes are microRNA targets. Cell *120*, 15–20.

Licatalosi, D.D., Mele, A., Fak, J.J., Ule, J., Kayikci, M., Chi, S.W., Clark, T.A., Schweitzer, A.C., Blume, J.E., Wang, X., et al. (2008). HITS-CLIP yields genome-wide insights into brain alternative RNA processing. Nature *456*, 464–469.

Licatalosi, D.D., Ye, X., and Jankowsky, E. (2020). Approaches for measuring the dynamics of RNA-protein interactions. Wiley Interdiscip. Rev. RNA *11*, e1565.

Liu, J., Carmell, M.A., Rivas, F.V., Marsden, C.G., Thomson, J.M., Song, J.J., Hammond, S.M., Joshua-Tor, L., and Hannon, G.J. (2004). Argonaute2 is the catalytic engine of mammalian RNAi. Science *305*, 1437–1441.

Maerkl, S.J., and Quake, S.R. (2007). A systems approach to measuring the binding energy landscapes of transcription factors. Science *315*, 233–237.

Martin, L., Meier, M., Lyons, S.M., Sit, R.V., Marzluff, W.F., Quake, S.R., and Chang, H.Y. (2012). Systematic reconstruction of RNA functional motifs with high-throughput microfluidics. Nat. Methods *9*, 1192–1194.

McGeary, S.E., Lin, K.S., Shi, C.Y., Pham, T.M., Bisaria, N., Kelley, G.M., and Bartel, D.P. (2019). The biochemical basis of microRNA targeting efficacy. Science *366*, eaav1741.

McGeary, S.E., Bisaria, N., Pham, T.M., Wang, P.Y., and Bartel, D.P. (2022). MicroRNA 3'-compensatory pairing occurs through two binding modes, with affinity shaped by nucleotide identity and position. Elife *11*, e69803.

Meister, G., Landthaler, M., Patkaniowska, A., Dorsett, Y., Teng, G., and Tuschl, T. (2004). Human Argonaute2 mediates RNA cleavage targeted by miRNAs and siRNAs. Mol. Cell *15*, 185–197.

Moore, M.J. (2005). From birth to death: the complex lives of eukaryotic mRNAs. Science *309*, 1514–1518.

Müller-McNicoll, M., and Neugebauer, K.M. (2013). How cells get the message: dynamic assembly and function of mRNA–protein complexes. Nat. Rev. Genet. *14*, 275–287.

Nakanishi, K., Weinberg, D.E., Bartel, D.P., and Patel, D.J. (2012). Structure of yeast Argonaute with guide RNA. Nature *486*, 368–374.

Nutiu, R., Friedman, R.C., Luo, S., Khrebtukova, I., Silva, D., Li, R., Zhang, L., Schroth, G.P., and Burge, C.B. (2011). Direct measurement of DNA affinity landscapes on a high-throughput sequencing instrument. Nat. Biotechnol. *29*, 659–664.

Pall, G.S., and Hamilton, A.J. (2008). Improved northern blot method for enhanced detection of small RNA. Nat. Protoc. *3*, 1077–1084.

Parker, J.S., Roe, S.M., and Barford, D. (2005). Structural insights into mRNA recognition from a PIWI domain-siRNA guide complex. Nature *434*, 663–666.

Rajewsky, N., and Socci, N.D. (2004). Computational identification of micro-RNA targets. Dev. Biol. *267*, 529–535.

Riggs, A.D., Bourgeois, S., and Cohn, M. (1970a). The lac repressor-operator interaction. 3. Kinetic studies. J. Mol. Biol. *53*, 401–417.

Riggs, A.D., Suzuki, H., and Bourgeois, S. (1970b). Lac repressor-operator interaction. I. Equilibrium studies. J. Mol. Biol. *48*, 67–83.

Rivas, F.V., Tolia, N.H., Song, J.J., Aragon, J.P., Liu, J., Hannon, G.J., and Joshua-Tor, L. (2005). Purified Argonaute2 and an siRNA form recombinant human RISC. Nat. Struct. Mol. Biol. *12*, 340–349.

Salomon, W.E., Jolly, S.M., Moore, M.J., Zamore, P.D., and Serebrov, V. (2015). Single-molecule imaging reveals that argonaute reshapes the binding properties of its nucleic acid guides. Cell *162*, 84–95.

Schirle, N.T., Sheu-Gruttadauria, J., Chandradoss, S.D., Joo, C., and MacRae, I.J. (2015). Water-mediated recognition of t1-adenosine anchors Argonaute2 to microRNA targets. eLife *4*, e07646.

Schuck, P. (1997). Reliable determination of binding affinity and kinetics using surface plasmon resonance biosensors. Curr. Opin. Biotechnol. *8*, 498–502.

Schwarz, D.S., Tomari, Y., and Zamore, P.D. (2004). The RNA-induced silencing complex is a Mg2+-dependent endonuclease. Curr. Biol. *14*, 787–791.

Selbach, M., Schwanhäusser, B., Thierfelder, N., Fang, Z., Khanin, R., and Rajewsky, N. (2008). Widespread changes in protein synthesis induced by microRNAs. Nature *455*, 58–63.

Sharma, D., Zagore, L.L., Brister, M.M., Ye, X., Crespo-Hernández, C.E., Licatalosi, D.D., and Jankowsky, E. (2021). The kinetic landscape of an RNA-binding protein in cells. Nature *591*, 152–156.

Sheu-Gruttadauria, J., Xiao, Y., Gebert, L.F., and MacRae, I.J. (2019). Beyond the seed: structural basis for supplementary microRNA targeting by human Argonaute2. EMBO J. *38*, e101153.

Shi, X., and Herschlag, D. (2009). Fluorescence polarization anisotropy to measure RNA dynamics. Methods Enzymol. *469*, 287–302.

Shin, C., Nam, J.W., Farh, K.K., Chiang, H.R., Shkumatava, A., and Bartel, D.P. (2010). Expanding the microRNA targeting code: functional sites with centered pairing. Mol. Cell *38*, 789–802.

Smith, C.S., Jouravleva, K., Huisman, M., Jolly, S.M., Zamore, P.D., and Grunwald, D. (2019). An automated Bayesian pipeline for rapid analysis of single-molecule binding data. Nat. Commun. *10*, 272.

Smolarsky, M., and Tal, M. (1970). Novel method for measuring polyuridylic acid binding to ribosomes. Biochim. Biophys. Acta *199*, 447–452.

Sugimoto, Y., König, J., Hussain, S., Zupan, B., Curk, T., Frye, M., and Ule, J. (2012). Analysis of CLIP and iCLIP methods for nucleotide-resolution studies of protein-RNA interactions. Genome Biol. *13*, R67.

Taliaferro, J.M., Lambert, N.J., Sudmant, P.H., Dominguez, D., Merkin, J.J., Alexis, M.S., Bazile, C., and Burge, C.B. (2016). RNA sequence context effects measured in vitro predict in vivo protein binding and regulation. Mol. Cell *64*, 294–306.

Tome, J.M., Ozer, A., Pagano, J.M., Gheba, D., Schroth, G.P., and Lis, J.T. (2014). Comprehensive analysis of RNA-protein interactions by high-throughput sequencing-RNA affinity profiling. Nat. Methods *11*, 683–688.

Turner, D.H., and Mathews, D.H. (2010). NNDB: the nearest neighbor parameter database for predicting stability of nucleic acid secondary structure. Nucleic Acids Res. *38*, D280–D282.

Van Nostrand, E.L., Freese, P., Pratt, G.A., Wang, X., Wei, X., Xiao, R., Blue, S.M., Chen, J.Y., Cody, N.A.L., Dominguez, D., et al. (2020). A large-scale binding and functional map of human RNA-binding proteins. Nature *583*, 711–719.

Virtanen, P., Gommers, R., Oliphant, T.E., Haberland, M., Reddy, T., Cournapeau, D., Burovski, E., Peterson, P., Weckesser, W., Bright, J., et al. (2020). SciPy 1.0: fundamental algorithms for scientific computing in Python. Nat Methods *17*, 261–272.

Wang, Y., Sheng, G., Juranek, S., Tuschl, T., and Patel, D.J. (2008). Structure of the guide-strand-containing Argonaute silencing complex. Nature *456*, 209–213.

Wee, L.M., Flores-Jasso, C.F., Salomon, W.E., and Zamore, P.D. (2012). Argonaute divides its RNA guide into domains with distinct functions and RNA-binding properties. Cell *151*, 1055–1067.

Winter, R.B., and von Hippel, P.H. (1981). Diffusion-driven mechanisms of protein translocation on nucleic acids. 2. The Escherichia coli repressor–operator interaction: equilibrium measurements. Biochemistry *20*, 6948–6960.

Wong, I., and Lohman, T.M. (1992). Allosteric effects of nucleotide cofactors on Escherichia coli Rep helicase-DNA binding. Science *256*, 350–355.

Wong, I., and Lohman, T.M. (1993). A double-filter method for nitrocellulose-filter binding: application to protein-nucleic acid interactions. Proc. Natl. Acad. Sci. U S A *90*, 5428–5432.

Xiao, Y., and MacRae, I.J. (2020). Robust differential microRNA targeting driven by supplementary interactions in vitro. RNA *26*, 162–174.

Zhao, J., Ohsumi, T.K., Kung, J.T., Ogawa, Y., Grau, D.J., Sarma, K., Song, J.J., Kingston, R.E., Borowsky, M., and Lee, J.T. (2010). Genome-wide identification of polycomb-associated RNAs by RIP-seq. Mol. Cell *40*, 939–953.

Zykovich, A., Korf, I., and Segal, D.J. (2009). Bind-n-Seq: high-throughput analysis of in vitro protein-DNA interactions using massively parallel sequencing. Nucleic Acids Res. *37*, e151.

STAR★METHODS

KEY RESOURCES TABLE

REAGENT or RESOURCE	SOURCE	IDENTIFIER
**Chemicals, peptides, and recombinant proteins**		
DMEM, high glucose, GlutaMAX Supplement	Thermo Fisher	10566024
Heat-inactivated FBS	Life Technologies	10082-147
Polybrene	Sigma	TR-1003
Puromycin dihydrochloride	Thermo Fisher	A1113803
Klenow fragment 3′-to-5′ exo-minus	New England Biolabs	M0212S
Streptavidin	New England Biolabs	N7021S
Heparin	Sigma	H4784
Protocatechuic acid	Aldrich	37580
Pseudomonas sp. protocatechuate 3,4-Dioxygenase	Sigma	P8279
Trolox	Aldrich	238813
Propyl gallate	Sigma	P3130
4-nitrobenzyl alcohol	Aldrich	N12821
Proteinase K	EMD Millipore	70663-5
AccuPrime *Pfx* DNA Polymerase	Invitrogen	12344024
3XFLAG	Sigma Aldrich	F4799
**Critical commercial assays**		
Anti-FLAG M2 paramagnetic beads	Sigma	M8823
Dynabeads MyOne Streptavidin T1	Life Technologies	65602
Protran nitrocellulose	Sigma	GE10600002
Hybond-XL	Cytiva	RPN303S
SuperScript III First-Strand Synthesis System	Invitrogen	18080051
TransIT-2020	Mirus Bio	MIR 5406
**Deposited data**		
Raw sequencing data from mouse RISC RBNS	This study	NCBI: PRJNA807105
Raw sequencing data from human RISC RBNS	McGeary et al. (2019)	GSE140220
**Experimental models: Cell lines**		
HEK 293T stably overexpressing FLAG-tagged mouse AGO2	This study	N/A
**Oligonucleotides**		
Table S1	This study	N/A
**Recombinant DNA**		
pScalps Puro EGFP 3XFLAG-AGO2	This study	N/A
psPAX2	Gift of Ken-Edwin Aryee	N/A
pMD2.G	Gift of Ken-Edwin Aryee	N/A
**Software and algorithms**		
Python v2.7.11	Python Software Foundation	https://www.python.org
Python module NumPy v1.16.6	Harris et al., 2020	https://numpy.org/
Python module SciPy v1.2.3	Virtanen et al., 2020	http://www.scipy.org
MATLAB vR2020b	Natick, MA: MathWorks	https://www.mathworks.com
CoSMoS pipeline v1	Smith et al. (2019)	https://github.com/qnano/cosmos_pipeline
Estimation of K_D values and kinetic parameters	This study	https://doi.org/10.6084/m9.figshare.19180952

CellPress
OPEN ACCESS

RESOURCE AVAILABILITY

Lead contact

Further information and requests for resources and reagents should be directed to, and will be fulfilled by, the lead contact, Phillip D. Zamore (phillip.zamore@umassmed.edu), or by completing the request form at https://www.zamorelab.umassmed.edu/reagents.

Materials availability

Plasmids generated in this study are available for non-commercial use upon request without restriction.

Data and code availability

RBNS sequencing data have been deposited at National Center for Biotechnology Information Sequence Read Archive and are publicly available as of the date of publication using accession number PRJNA807105.

All published software required to reanalyze the data reported in this paper is described in the quantification and statistical analysis section below. All original code has been deposited at https://figshare.com/articles/software/MicroRNA-binding_thermodynamics_and_kinetics_by_RNA_Bind-n-Seq/19180952 and is publicly available as of the date of publication.

Any additional information required to reanalyze the data reported in this paper is available from the lead contact upon request.

EXPERIMENTAL MODEL AND SUBJECT DETAILS

Cell lines

Studies used HEK293T cells stably overexpressing FLAG-AGO2. AGO2 cDNA was amplified by RT-PCR from mouse testis total RNA. Restriction cloning was used to add the AGO2 coding sequence to pScalps Puro EGFP, fusing the sequence in-frame with N-terminal 3XFLAG tag. Lentivirus transfer vectors were packaged by co-transfection with psPAX2 and pMD2.G (4:3:1) using TransIT-2020 (Mirus Bio) in HEK293T cells. Supernatant containing lentivirus was used to transduce HEK293T cells in the presence of 16 μg/ml polybrene (Sigma) to obtain stable FLAG-AGO2-expressing cell lines. Three sequential transductions were performed to maximize recombinant protein production. The transduced cells were selected in the presence of 2 $\mu g \cdot ml^{-1}$ puromycin for 2 weeks, then the cells expressing the 5% highest EGFP fluorescence were selected by FACS (UMASS Medical School Flow Cytometry Core) and expanded. Cells were cultured at 37°C, 5% CO_2 in DMEM (Gibco, Life Technologies) supplemented with 10% heat-inactivated fetal bovine serum (Sigma).

METHOD DETAILS

RISC purification

Cells stably expressing recombinant FLAG-AGO2 protein were expanded. Cells were washed once with ice-cold PBS, collected by scraping and centrifuged at 500 × *g* for 5 min. Cell pellets were flash-frozen in liquid nitrogen and stored at −80°C. Cell extract was essentially prepared as described (Dignam et al., 1983). Briefly, the cell pellet was washed three times in ice-cold PBS and once in Buffer A (10 mM HEPES-KOH pH 7.9, 10 mM potassium acetate, 1.5 mM magnesium acetate, 0.01% (w/v) CHAPS, 0.5 mM DTT, 1 mM AEBSF, hydrochloride, 0.3 μM Aprotinin, 40 μM Bestatin, hydrochloride, 10 μM E-64, 10 μM Leupeptin hemisulfate). Next, the pellet was resuspended in twice its volume with buffer A and incubated on ice for 20 min to allow the cells to swell. The cells were subsequently lysed on ice with a Dounce homogenizer using 40 strokes of a tight pestle (B type). The homogenate was centrifuged at 2,000 × *g* to remove nuclei and cell membranes. Next, 0.11 volumes (relative to the volume of the clarified supernatant from the low speed centrifugation) of Buffer B (300 mM HEPES-KOH, pH 7.9, 1.4 M potassium acetate, 30 mM magnesium acetate, 0.01% (w/v) CHAPS, 0.5 mM DTT, 1 mM AEBSF, hydrochloride, 0.3 μM Aprotinin, 40 μM Bestatin, hydrochloride, 10 μM E-64, 10 μM Leupeptin, hemisulfate) was added, followed by centrifugation at 100,000 × *g* for 20 min at 4°C; the supernatant corresponds to the S100 extract. Ice-cold 80% (w/v) glycerol was then added to achieve a 20% (w/v) final glycerol concentration and mixed by gentle inversion. S100 was aliquoted, frozen in liquid nitrogen, and stored at −80°C.

To capture AGO2 protein, clarified lysate was incubated for 2 h at 4°C rotating with 20 μl anti-FLAG M2 paramagnetic beads (Sigma) per ml lysate. Beads were washed three times with wash buffer (30 mM HEPES-KOH, pH 7.9, 120 mM potassium acetate, 3.5 mM magnesium acetate, 2 mM DTT, 0.01% (w/v) CHAPS). Immobilized AGO2 was loaded by incubating with 1 μM single-stranded miRNA guide (Table S2) in wash buffer for 1 h at 37°C. Unbound miRNA guide was removed by washing the beads three times with wash buffer. AGO2 and AGO2:miRNA were eluted for 1 h at room temperature with 100 $ng \cdot \mu l^{-1}$ 3XFLAG peptide in wash buffer.

miRISC was purified as described (Flores-Jasso et al., 2013). Briefly, the assembled miRISC was incubated overnight at 4°C with a biotinylated, 2′-*O*-methyl capture oligonucleotide (Table S2) linked to streptavidin paramagnetic beads (Dynabeads MyOne Streptavidin T1, Life Technologies). miRISC was eluted with a competitor oligonucleotide (Table S2) for 2 h at room temperature. Excess competitor oligonucleotide was removed by incubating the eluate with streptavidin paramagnetic beads (Dynabeads MyOne Streptavidin T1, Life Technologies) for 15 min at room temperature. Finally, miRISC was dialyzed at 4°C against three changes (3 h each) of a 3,000-fold excess of wash buffer supplemented with 20% (w/v) glycerol. miRISC was aliquoted, frozen in liquid

nitrogen, and stored at −80°C. For single-molecule analysis, guide strands were labeled with 3′ Alexa Fluor 555 (Life Technologies).

Northern blotting

Northern blotting was essentially performed as described (Pall and Hamilton, 2008). Briefly, miRNA guide standards and miRISC were first resolved on a denaturing 15% polyacrylamide gel, transferred to Hybond-XL (Cytiva) by semi-dry transfer at 20 V for 1 h, and the RNA crosslinked to the membrane with 0.16 M EDC in 0.13 M 1-methylimidazole, pH 8.0, at 60°C for 1 h. The crosslinked membrane was pre-hybridized in Church's buffer (1% w/v BSA, 1 mM EDTA, 0.5 M phosphate buffer, and 7% w/v SDS) at 37°C for 1 h. Radiolabeled, 25 pmol 5′ ^{32}P-DNA probe (Table S2) in Church buffer was added to the membrane and allowed to hybridize overnight at 37°C, followed by two washes with 2× SSC containing 0.1% w/v SDS and two washes with 1× SSC containing 0.1% w/v SDS at 37°C for 15 min. The membrane was air dried and exposed to a storage phosphor screen.

Quantification of active miRISC by double-filter binding assay

Binding assays were essentially performed as described (Wee et al., 2012). Double-filter binding assays measured the equilibrium binding of active, binding-competent miRISC with 28-nt long RNA target fully complementary to the miRNA guide. To block cleavage, the target RNA contained a phosphorothioate linkage flanked by 2′-*O*-methyl ribose at positions t10 and t11 (Schwarz et al., 2004; Rivas et al., 2005; Wee et al., 2012). Binding reactions were performed in 5 μl in the presence of 30 mM HEPES-KOH, pH 7.9, 120 mM potassium acetate, 3.5 mM magnesium acetate, 2 mM DTT, 0.01% (w/v) CHAPS. A 5′ ^{32}P-RNA target (0.5 nM) complementary to the seed region of the miRNA guide (Table S2) was incubated with a range of miRISC concentrations from 0.05 nM to 5 nM. The assay also included a no-miRISC binding reaction using miRISC storage buffer. Binding reactions were incubated at 37°C for 1 hr. RNA binding was measured by capturing protein-RNA complexes on Protran nitrocellulose (Whatman, GE Healthcare Bioscience, Pittsburgh, PA) and unbound RNA on a Hybond-XL (Cytiva) in a Bio-Dot apparatus (Bio-Rad, Hercules, CA). After applying the sample under vacuum, membranes were washed with 10 μl equilibration buffer (30 mM HEPES-KOH, pH 7.9, 120 mM potassium acetate, 3.5 mM magnesium acetate, 2 mM DTT). Membranes were air-dried and signals detected by phosphorimaging.

Co-localization single-molecule spectroscopy

Single-stranded RNA targets were generated as described (Salomon et al., 2015). Typically, 100 pmol RNA target (Table S2) was mixed with a 1.5-fold molar excess of Klenow template oligonucleotide (Table S2) in 7.5 μl of 10 mM HEPES-KOH, pH 7.4, 20 mM sodium chloride, and 0.1 mM EDTA. Samples were incubated at 90°C for 5 min in a heat block. The heat block was then switched off and allowed to cool to room temperature. Afterwards, the annealed strands (30% of final reaction volume) were added without further purification to a 3′ extension reaction, comprising 1× NEB Buffer 2 (New England Biolabs, Ipswich, MA), 1 mM dATP, 1 mM dCTP, 0.12 mM Alexa Fluor 647-aminohexylacrylamido-dUTP (Life Technologies), and 0.2 U/μl Klenow fragment (3′-to-5′ exo-minus, New England Biolabs) and incubated at 37°C for 1 h. The reaction was quenched with 500 mM (f.c.) ammonium acetate and 20 mM (f.c.) EDTA. A 15-fold molar excess of 'trap' oligonucleotide (Table S2) was added to the Klenow template oligonucleotide. The entire reaction was precipitated overnight at −20°C with three volumes of ethanol. The labeled target was recovered by centrifugation, dried, dissolved in loading buffer (7 M Urea, 25 mM EDTA), incubated at 95°C for 5 min, and resolved on a 6% polyacrylamide gel and isolated by electroelution.

Single-molecule experiments were performed and analyzed as described (Smith et al., 2019). Fresh cover glasses were prepared for each day of imaging. Cover glasses (Gold Seal 24 Å∼ 60 mM, No. 1.5, Cat. #3423), and glass coverslips (Gold Seal 25 Å∼ 25 mM, No. 1, Cat. #3307) were cleaned by sonicating for 30 min in NanoStrip (KMG Chemicals, Houston, TX), washed with 10 changes of deionized water, and dried with a stream of nitrogen. Two ∼2 mm diameter lines of high vacuum grease (Dow Corning, Midland, MI) were applied to the cover glass to create a flow cell. Three layers of self-sticking labeling tape (Fisher, Cat. No. 159015R) were applied outside of the flow cell. The coverslip was placed on top of the cover glass with a ∼0.5 mm gap between the cover glass and coverslip. To minimize non-specific binding of protein and RNA to the glass surface, microfluidic chambers were incubated with 2 mg·ml^{-1} poly-L-lysine-graft-PEG-biotin in 10 mM HEPES-KOH, pH 7.5, at room temperature for 30 min and washed extensively with imaging buffer (30 mM HEPES-KOH, pH 7.9, 120 mM potassium acetate, 3.5 mM magnesium acetate, 20% (w/v) glycerol) immediately before use. To immobilize biotinylated RNA targets, streptavidin (0.01 mg·ml^{-1}, Sigma) was incubated for 5 min in each microfluidic chamber. Unbound streptavidin was washed away with imaging buffer.

Immediately before each experiment, a flow cell was incubated for 2 min with imaging buffer supplemented with 75 μg·ml^{-1} heparin (Sigma H4784), oxygen scavenging system (Crawford et al., 2008; Aitken et al., 2008) (2.5 mM protocatechuic acid [Aldrich 37580] and 0.5 U·ml^{-1} *Pseudomonas sp*. protocatechuate 3,4-Dioxygenase [Sigma P8279]), triplet quenchers (Dave et al., 2009) (1 mM Trolox [Aldrich 238813], 1 mM propyl gallate (Sigma P3130), and 1 mM 4-nitrobenzyl alcohol [Aldrich N12821]). The chamber was then filled with ∼100 pM target in imaging buffer supplemented with 75 μg·ml^{-1} heparin, oxygen scavenging system, and triplet quenchers. Target deposition was monitored by taking a series of images; once the desired density was achieved, the flow cell was washed three times with imaging buffer supplemented with oxygen scavenging system and triplet quenchers. A syringe pump (KD Scientific, Holliston, MA) running in withdrawal mode at 0.15 ml·min^{-1} was applied to the flow cell outlet to introduce AGO2:miRNA complex (pre-heated to 37°C) supplemented with an oxygen scavenging system and triplet quenchers. Typically, 3,000 frames were collected at 5 frames per s. A digitally-controlled heater (TP-LH, Tokai Hit) maintained objective temperature at 42°C. A custom fabricated heating stage (Smith et al., 2019) was heated to 40°C to achieve a sample temperature of 37°C. The temperature on

the surface of the cover glass was independently monitored with a Type E, 0.25 mM O.D. thermocouple (Omega Engineering Inc., Sutton, MA) inserted between the top and the bottom cover glasses.

Imaging was performed on an IX81-ZDC2 zero-drift inverted microscope equipped with a cell^TIRF motorized multicolor TIRF illuminator with 561 and 640 nm 100 mW lasers and a 100×, oil immersion, 1.49 numerical aperture UAPO N TIRF objective with FN=22 (Olympus, Tokyo, Japan). Fluorescence signals were split with a main dichroic mirror (Olympus OSF-LFQUAD) and triple emission filter (Olympus U-CZ491561639M). The primary image was relayed to two ImagEM X2 EM-CCD cameras (C9100-23B, Hamamatsu Photonics, Hamamatsu, Japan) using a Cairn three-way splitter equipped with a longpass dichroic mirror (T635lpxr-UF2, Chroma). Illumination and acquisition parameters were controlled with cell^TIRF and MetaMorph software (Molecular Devices, Sunnyvale, CA), respectively.

Images were recorded as uncompressed TIFF files and merged into stacked TIFF files. Images were processed using the pipeline (Smith et al., 2019) as described in the manual. Co-localization events required that (1) the intensity of AGO2:miRNA complex >150 photons, (2) ratio intensity of the AGO2:miRNA complex to the local background >1, (3) the distance between the target and guide was < 1.2 pixel, and (4) sigma < 4.6. To exclude short, non-specific events, the minimal event duration was set to 2 frames. To overcome short temporary loss of miRISC fluorescent signal due to fluorescent dye blinking, the gap parameter was set to 2 frames. Only the first binding event at each target location was used to estimate arrival time and dwell time, to minimize errors caused by occupation of sites by photobleached molecules. The same analysis was automatically performed on 'dark' locations, i.e., regions that contained no target molecules; these served as a control for non-specific binding of AGO2:miRNA complex to the surface of the cover glass.

RNA bind-*n*-Seq for de novo site discovery and K_D measurements

Two libraries of RNA oligonucleotides, each containing a central region of 20 random-sequence positions (Table S2), were synthesized with equal ratio of bases (25:25:25:25) (IDT), 5′ ^{32}P-radiolabeled, and gel-purified. After phenol-chloroform extraction and ethanol precipitation, RNA was denatured at 90°C for 1 min, annealed to BRTP primer (Table S2) and reverse transcribed using SuperScript III. RNA was degraded by alkaline hydrolysis using 0.4 M sodium hydroxide for 1 h at 55°C, and cDNA was recovered by ethanol precipitation. The sample was then amplified with AccuPrime *Pfx* DNA Polymerase (Invitrogen). The reactions were run on a 2% agarose gel, amplicons were purified then sequenced using a NextSeq 500 (Illumina) to obtain 75-nt, single-end reads.

Because of the randomness of the central region, sequence composition differed between the two libraries. For example, frequencies of the four nucleotides at each position within the randomized region of RNA molecules varied between the two libraries (Figure S1A). RNA pool 1 was used in RBNS of AGO2:miR-34b trials 1 and 2, AGO2:let-7a trial1, and let-7a trials 1 and 2; RNA pool 2 was used in RBNS of AGO2:let-7a trial 2, AGO2:let-7a trials 1 and 2 (recovery of bound RNA using M2 FLAG beads), AGO2:miR-449a – blockers trials 1 and 2, AGO2:miR-449a + blockers trials 1 and 2, miR-34b trials 1 and 2, miR-449a trials 1 and 2, and association experiments. DNA blocking oligonucleotides were synthesized (IDT) and annealed to RNA library in 30 mM HEPES-KOH, pH 7.5, 120 mM potassium acetate, 3.5 mM magnesium acetate using a 1:1.2 molar ratio of RNA pool to DNA blockers by first incubating at 95°C for 1 min, then at 65°C for 10 min, and finally cooled to room temperature.

Each experiment included five or six binding reactions. The highest concentration of miRISC used corresponded to 40% (v/v) of the stock solution and equaled 0.8–5 nM (f.c.) active protein. For additional reactions, the stock was serially diluted 3.2-fold in storage buffer. Each experiment also included a mock binding reaction (no-RISC control) using protein storage buffer without miRISC. For each miRNA, we performed an additional binding reaction using protein storage buffer with miRNA guide at the highest miRISC concentration assayed, but lacking AGO2 protein. All binding reactions (20 μl) were performed in 25 mM HEPES-KOH, pH 7.9, 110 mM potassium acetate, 3.5 mM magnesium acetate, 0.01% (w/v) CHAPS, 2 mM DTT, 8% (w/v) glycerol and contained 100 nM (f.c.) RNA library. To reduce non-specific binding, each reaction also included 2.5 $\mu g \cdot \mu l^{-1}$ BSA and 0.5 $\mu g \cdot \mu l^{-1}$ yeast tRNA. Reactions were incubated for 2 h at 37°C and then filtered through a Protran nitrocellulose membrane (Whatman, GE Healthcare Bioscience, Pittsburgh, PA) on top of a Hybond-XL (Cytiva) nylon membrane in a Bio-Dot apparatus (Bio-Rad, Hercules, CA). To reduce retention of free single-stranded RNA, we pre-conditioned nitrocellulose and nylon membranes prior to use as described (Smolarsky and Tal, 1970; Wong and Lohman, 1993). Nitrocellulose filters were pre-soaked in 0.4 M potassium hydroxide for 10 min. Nylon filters were incubated in 0.1 M EDTA, pH 8.2 for 10 min, washed three times in 1 M sodium chloride for 10 min each followed by a quick rinse (~15 s) in 0.5 M sodium hydroxide. Nitrocellulose and nylon filters were then rinsed in water until the pH returned to neutral and equilibrated in wash buffer (20 mM HEPES-KOH, pH 7.9, 100 mM potassium acetate, 3.5 mM magnesium acetate, 1 mM DTT) for at least 1 h at 37°C. After applying the sample under vacuum, membranes were washed with 100 μl wash buffer for 3 s. Membranes were air-dried and signals detected by phosphorimaging to monitor binding. The nitrocellulose membranes containing miRISC-bound RNA were excised and incubated with 1 $\mu g \cdot \mu l^{-1}$ Proteinase K (Thermo Fischer) in 100 mM Tris-HCl, pH 7.5, 10 mM EDTA, 150 mM sodium chloride, 1% (w/v) SDS for 1 h at 45°C shaking at 300 rpm. After phenol-chloroform extraction and ethanol precipitation, RNA was reverse transcribed, amplified, and sequenced using the procedure described above for the RNA pool.

RNA bind-*n*-Seq using paramagnetic beads

Binding reactions were assembled as described above. After incubation for 1 h at 37°C, reactions were transferred to tubes containing 10 μl anti-FLAG M2 paramagnetic beads (Sigma). Prior to use, beads were washed three times with wash buffer

(20 mM HEPES-KOH, pH 7.9, 100 mM potassium acetate, 3.5 mM magnesium acetate, 1 mM DTT) and incubated for 1 h at 37°C with wash buffer containing 2.5 $\mu g \cdot \mu l^{-1}$ BSA and 0.5 $\mu g \cdot \mu l^{-1}$ yeast tRNA. After adding binding reactions to beads, samples were incubated at 37°C for 1 h. Beads were captured in a magnetic stand, the supernatant containing unbound RNA removed, and the beads washed with 100 μl wash buffer for 3 s. miRISC and bound RNA were eluted twice for 45 min at room temperature with 100 $ng \cdot ml^{-1}$ 3XFLAG peptide in wash buffer. RNA libraries were prepared as described above.

RNA bind-*n*-Seq for kinetic measurements

The nitrocellulose and nylon membranes were prepared as described above. Binding reactions were performed in 25 mM HEPES-KOH, pH 7.9, 110 mM potassium acetate, 3.5 mM magnesium acetate, 0.01% (w/v) CHAPS, 2 mM DTT, 8% (w/v) glycerol, and contained 100 nM (f.c.) RNA library (RNA pool 2) and 136 pM (trial 1) or 170 pM (trial 2) AGO2:let-7a. To reduce non-specific binding, each reaction also included 2.5 $\mu g \cdot \mu l^{-1}$ BSA and 0.5 $\mu g \cdot \mu l^{-1}$ yeast tRNA. All components except the RNA pool were combined to generate a master mix, which was aliquoted and placed at 37°C. The RNA pool was added to the first aliquot, and the timer started. After 1 h, the RNA pool was added to the second aliquot. After 1.5 h, the RNA pool was added to the third aliquot. This procedure was repeated until all aliquots received RNA pool at the desired time points. After 2 h from initiating the first binding reaction, all the samples were applied under vacuum to the membranes, which were washed with 100 μl wash buffer. The nitrocellulose membranes containing miRISC-bound RNA were excised, RNA extracted, reverse-transcribed, amplified, and sequenced as described above.

QUANTIFICATION AND STATISTICAL ANALYSIS

Quantification of active miRISC by double-filter binding assay

To measure concentration of active, binding-competent miRISC, titration data were fit to

$$f(r) = f_{max} \times \frac{r + K_D + n - \sqrt{(r + K_D + n)^2 - 4 \times r \times n}}{2 \times n}$$

where *K*D is the apparent dissociation constant, *r* is the molar ratio of [RISC] to [RNA], *n* is the stochiometric equivalence point, *f* is the fraction bound, *f*max is the maximum fraction bound.

Co-localization single-molecule spectroscopy

The individual datasets were saved and combined. The binding rate (k_{on}) was determined by fitting the cumulative fractions of miRISC arrivals to:

$$f(t) = 1 - (1 - h) \times e^{-k_{on} \times t} - h \times e^{-(k_{on} + k_{NS}) \times t}$$

and reported per time unit and concentration of introduced miRISC. A dwell time distribution was fitted as

$$f(t) = N \times e^{-k_{off} \times t} + A \times e^{-k_{NS_1} \times t} + B \times e^{-k_{NS_2} \times t}.$$

Parameters relative to non-specific association of miRISC with the glass surface (k_{NS}, on-rate for non-specific arrivals; *h*, fraction of control locations having received non-specific arrivals; k_{NS1} and k_{NS2}, off-rate for non-specific binding events and rate of photobleaching; *A* and *B*, their respective amplitudes) were determined from the fitting of data for control locations. Values of k_{on} and k_{off} were derived from data collected from >900 individual RNA target molecules. Error was evaluated by 1,000-cycle bootstrapping of 90% of the data.

Quality control of high-throughput sequencing data

Only Illumina reads containing TGG (the first nucleotides of the 3′ adapter) at positions 21–23 were analyzed. Sequences were filtered (Phred quality score ≥20 for all nucleotides, and "N" base calls disallowed), and the 3′ adapter sequence (5′-TGG AAT TCT CGG GTG CCA AGG-3′) removed.

Enrichment values

Occurrences of all 10-nt long motifs (10-mers) were counted in all the reads of each RBNS sample. These counts were then divided by the total count of all 10-mers to give motif frequencies. Enrichment of a motif was computed as the ratio of the motif frequency in the protein-bound samples over the frequency in the RNA pool. *Z*-score of a motif was computed as $Z = \frac{R - \overline{R}}{S}$ where *R* is enrichment of the motif, $\overline{R}$ is the mean of enrichment values of all 10-mers, and *S* is the sample standard deviation of enrichment values of all 10-mers. A motif was considered significant if its *Z*-score was ≥99.9 percentile.

De novo site discovery

Enrichments in the library from the binding reaction with the greatest miRISC concentration were used for the following iterative procedure: (1) enrichment values of all 10-mers were calculated; (2) the hundred most enriched 10-mers were interrogated for

CellPress
OPEN ACCESS

base-pairing with the guide miRNA; (3) the most enriched site type was identified; (4) Z-scores of motifs belonging to the site type were compared to the Z-score threshold; (5) all reads containing the binding site were masked in the miRISC-bound library and the RNA pool so that stepwise enrichments of subsequent 10-mers could be used to eliminate subsequent 'shadow' motifs; (6) all enrichment values were then recalculated on the masked read sets to obtain the resulting most enriched 10-mers. This process continued until the Z-score of the most enriched binding site (calculated from the original enrichment values) was < 99.9 percentile.

To identify a binding site at each iteration, the one-hundred most enriched 10-mers were tested for base-pairing with the guide miRNAs. If perfect complementarity was not observed, the 10-mer was tested for any of the following in this order: (1) complementarity to nine contiguous miRNA positions, allowing a single bulged target nucleotide; (2) complementarity to ten contiguous miRNA positions while allowing for wobble pairing; (3) complementarity to ten contiguous miRNA positions while allowing a non-wobble mismatch. If none of these configurations allowed assigning the motif to a binding site, the procedure was repeated with two 9-mers within the 10-mer, the three 8-mers within the 10-mer, etc., until a configuration of base-pairing was identified.

Read assignments

Each sequencing read in RNA pool and miRISC-bound libraries was interrogated for presence of all binding sites of interest. The entire single-stranded sequence was interrogated: the 20-nt random-sequence region flanked by constant primer-binding sequences in the case when blockers were not used and the 20-nt random-sequence region flanked by 4 nucleotides of constant primer-binding sequence on either side in the case when blockers were annealed to the RNA pool. For analysis of published datasets (McGeary et al., 2019), the entire 87-nt sequenced encompassing the 37-nt random sequence region and constant primer-binding regions was searched. A read was assigned to a site category if it contained one single binding motif. Reads containing multiple instances of binding sites (from the same or a different site category) and reads containing partially overlapping sites were not included in the analysis and represented $\leq 1\%$ of libraries. Reads that did not have any of binding motifs of interest were classified as reads with a no-site.

Modeling of RBNS experiments

RBNS data was modeled by simulating the equilibrium binding of an RNA Binding Protein (RBP) with an RNA input pool. The stock solution of the RBP was set to 2.1 nM (dataset 1) and 8.1 nM (datasets 2 and 3). Each in silico experiment included five binding reactions. The highest concentration of the RBP used corresponded to 40% (v/v) of the stock solution. For additional reactions, the stock was serially diluted 3.2-fold. Each experiment also included a mock binding reaction (no-RISC control). We considered the RBP binding to an RNA pool (100 nM f.c.) containing four binding sites with affinities equal to 5, 20, 100 and 500 pM (datasets 1 and 2) or 10, 50, 250 and 1,000 pM (dataset 3). The concentration of specific motifs was set to 15 pM. We also included nonspecific binding sites ($K_{D,\ nosite} = 5$ nM). We constructed a system of equations relating the concentrations of the free and bound states of RPB and binding sites to the K_D values for each binding site and the total concentrations of each species. This system was solved numerically for each input value of RBP in MATLAB using function *fsolve*. We allowed recovery of 0.1 nM background RNA.

Kinetic RBNS data was modeled by simulating the association of an RBP with an RNA input pool. Concentration of the RBP was set to 150 pM. Each in silico experiment included fourteen time points, ranging from 0 to 7,200 s. We considered the RBP binding to an RNA pool (100 nM f.c.) containing four binding sites with k_{on}^1=0.1 nM^{-1}s^{-1}, k_{on}^2=0.01 nM^{-1}s^{-1}, k_{on}^3=0.001 nM^{-1}s^{-1}, k_{on}^4=0.0001 nM^{-1}s^{-1}, $k_{on}^{no\text{-}site}$=0.0001 nM^{-1}s^{-1} and k_{off}^1=0.0001 s^{-1}, k_{off}^2=k_{off}^3=k_{off}^4=0.01 s^{-1}, $k_{off}^{no\text{-}site}$=10 s^{-1} (dataset 1) and k_{on}^1=k_{on}^2=k_{on}^3=k_{on}^4=0.1 nM^{-1}s^{-1}, $k_{on}^{no\text{-}site}$=0.001 nM^{-1}s^{-1} and k_{off}^1=0.00001 s^{-1}, k_{off}^2=0.001 s^{-1}, k_{off}^3=0.01 s^{-1}, k_{off}^4=0.1 s^{-1}, $k_{off}^{no\text{-}site}$=10 s^{-1} (dataset 2). We constructed a system of equations relating the concentrations of the free and bound states of RPB and binding sites to the K_D values for each binding site and the total concentrations of each species. This system was solved numerically for each input value of RBP in MATLAB using function *solve*. We allowed recovery of 0.1 nM background RNA.

Concentrations of each site recovered in equilibrium or kinetic RBNS in silico experiments were converted to number of molecules. The input RNA pool and bound RNA were subsampled with no replacement to yield 20×10^6 molecules—our typical sequencing depth.

Maximum likelihood estimation (MLE) of parameters from RBNS data

Let $\mathcal{M}$ be a mathematical model that predicts read counts of each binding site type given information about the RNA pool and a set of K_D values for each site type. Let $\mathcal{D}$ be observed data from RNA sequencing of n binding reactions. Maximum Likelihood Estimation (MLE) is used to estimate K_D values, so that $\mathcal{D}$ is the most probable given $\mathcal{M}$. We note the likelihood function $\mathcal{L}(\mathcal{D}|\mathcal{M})$:

$$\mathcal{L}(\mathcal{D}|\mathcal{M}) = \mathcal{L}\left((\text{Reads}_1,\ \ldots,\ \text{Reads}_j,\ \ldots,\text{Reads}_n)\middle|(K_{D_1},\ \ldots,\ K_{D_i},\ \ldots,\ K_{D_m})\right) \quad \text{(Equation 1)}$$

where Reads_j is sequencing data from the binding reaction j and K_{D_i} is dissociation constant for binding site i. Binding in reaction j is independent from binding in reaction $j+1$, so the joint probability is a product of individual probabilities:

$$P\left((\text{Reads}_1,\ \ldots,\ \text{Reads}_j,\ \ldots,\text{Reads}_n)\middle|(K_{D_1},\ \ldots,\ K_{D_i},\ \ldots,\ K_{D_m})\right) = \prod_{j=1}^{n} P\left(\text{Reads}_j\middle|(K_{D_1},\ \ldots,\ K_{D_i},\ \ldots,\ K_{D_m})\right) \quad \text{(Equation 2)}$$

Each probability is a value comprised between 0 and 1. Multiplying small values is prone to numerical underflow and introduces errors because the computer can only store a certain number of digits. Therefore, we describe $\mathcal{L}$ in terms of log conditional probabilities:

$$\mathcal{L}(\mathcal{D}|\mathcal{M}) = \ln\left[P\left(\left(\text{Reads}_1,\ \ldots,\ \text{Reads}_j,\ \ldots, \text{Reads}_n\right)\middle|\left(K_{D_1},\ \ldots,\ K_{D_i},\ \ldots,\ K_{D_m}\right)\right)\right]$$

$$\mathcal{L}(\mathcal{D}|\mathcal{M}) = \ln\left[\prod_{j=1}^{n} P\left(\text{Reads}_j\middle|\left(K_{D_1},\ \ldots,\ K_{D_i},\ \ldots,\ K_{D_m}\right)\right)\right]$$

$$\mathcal{L}(\mathcal{D}|\mathcal{M}) = \sum_{j=1}^{n} \ln\left[P\left(\text{Reads}_j\middle|\left(K_{D_1},\ \ldots,\ K_{D_i},\ \ldots,\ K_{D_m}\right)\right)\right] \quad \text{(Equation 3)}$$

In the binding reaction j, observations from RNA sequencing follow Negative Multinomial Distribution with possible outcomes $\{\text{site}_1,\ \text{site}_2,\ \ldots,\ \text{site}_{m-1},\ \text{noiste}\}$. Therefore, the probability mass function is:

$$P_j\left(\left(reads_{1,j},\ \ldots, reads_{i,j},\ \ldots,\ reads_{m-1,j}\middle| reads_{m,j}\right),\ \{p_1,\ \ldots,\ p_i,\ \ldots,\ p_{m-1}\}\right)$$
$$= \left(\sum_{i=1}^{m} reads_{i,j} - 1\right)! \times \frac{p_{m,j}^{reads_{m,j}}}{(reads_{m,j} - 1)!} \times \prod_{i=1}^{m-1} \frac{p_{i,j}^{reads_{i,j}}}{(reads_{i,j})!} \quad \text{(Equation 4)}$$

where pi,j is the exected frequency of a binding site type i in a binding reaction j and is given by the mathematical model:

$$p_{i,j} = \frac{x_{i,j}}{\sum_{i=1}^{m} x_{i,j}} \quad \text{(Equation 5)}$$

with xi,j being the concentration of a binding site i in binding reaction j predicted by $\mathcal{M}$.

After combining Equations (3), (4), and (5), $\mathcal{L}(\mathcal{D}|\mathcal{M})$ becomes:

$$\mathcal{L}(\mathcal{D}|\mathcal{M}) = \sum_{j=1}^{n} \ln\left[\left(\sum_{i=1}^{m} reads_{i,j}\right)! \times \frac{reads_{m,j}}{\sum_{i=1}^{m} reads_{i,j}} \times \prod_{i=1}^{m} \frac{\left(x_{i,j}/\sum_{i=1}^{m} x_{i,j}\right)^{reads_{i,j}}}{(reads_{i,j})!}\right] \quad \text{(Equation 6)}$$

Cost function for parameter fitting

We define a cost function, f_{cost}, as $f_{\text{cost}} = -\mathcal{L}(\mathcal{D}|\mathcal{M})$. (Equation 7)

A cost function maps values of variables into a real number, intuitively representing some "cost" associated with the event. An optimization procedure seeks to maximize the likelihood function, so that the model looks the most like the data. Because we define the cost function as the opposite of the likelihood function, f_{cost} will be minimized upon fitting.

$$f_{\text{cost}} = -\sum_{j=1}^{n} \ln\left[\left(\sum_{i=1}^{m} reads_{i,j}\right)! \times \frac{reads_{m,j}}{\sum_{i=1}^{m} reads_{i,j}} \times \prod_{i=1}^{m} \frac{\left(x_{i,j}/\sum_{i=1}^{m} x_{i,j}\right)^{reads_{i,j}}}{(reads_{i,j})!}\right] \quad \text{(Equation 8)}$$

Using product, quotient and power rules of natural logarithm, Equation (8) can be simplified to yield:

$$f_{\text{cost}} = \sum_{j=1}^{n} \left[\ln((reads_{i,j})!) - \ln\left(\left(\sum_{i=1}^{m} reads_{i,j}\right)!\right) - \ln\left(\frac{reads_{m,j}}{\sum_{i=1}^{m} reads_{i,j}}\right) + \ln\left(\sum_{i=1}^{m} x_{i,j}\right) \times \sum_{i=1}^{m} reads_{i,j} - \sum_{i=1}^{m}\left(reads_{i,j} \times \ln(x_{i,j})\right)\right] \quad \text{(Equation 9)}$$

We note that some terms in the expression of f_{cost} do not depend on parameters from the mathematical model. Therefore, f_{cost} can be written as

$$f_{\text{cost}} = \sum_{j=1}^{n} \left[\ln\left(\sum_{i=1}^{m} x_{i,j}\right) \times \sum_{i=1}^{m} reads_{i,j} - \sum_{i=1}^{m}\left(reads_{i,j} \times \ln(x_{i,j})\right)\right] + C$$

where $C = \sum_{j=1}^{n} \left[\ln((reads_{i,j})!) - \ln\left(\left(\sum_{i=1}^{m} reads_{i,j}\right)!\right) - \ln\left(\frac{reads_{m,j}}{\sum_{i=1}^{m} reads_{i,j}}\right)\right]$

Inclusion of a constant term may result in a loss of significant figures. Moreover, a function f and a function f+C have the same optima; therefore minimizing Equation (9) is equivalent to minimizing

$$f_{\text{cost}} = \sum_{j=1}^{m}\left[\ln\left(\sum\nolimits_{i=1}^{m} x_{i,j}\right) \times \sum_{i=1}^{m} reads_{i,j} - \sum_{i=1}^{m}\left(reads_{i,j} \times \ln(x_{i,j})\right)\right] \quad \text{(Equation 10)}$$

Implementation of MLE

Minimization of the cost function is performed by *minimize* function from a Python-based library SciPy using the Limited-memory Broyden–Fletcher–Goldfarb–Shanno algorithm (L-BFGS) with default parameters on acceptance of convergence. *Minimize* requires two inputs: the function f_{cost} to minimize and an initial guess of the variables. Both MLE of K_D values and MLE of kinetic parameters use f_{cost} defined above, but with xi,j coming from the appropriate mathematical model (see Mathematical Model for MLE of KD values and Mathematical Model for MLE of kon and koff values below). The BFGS method does not require the gradient of the cost function, as it can be estimated using fine differences. Nevertheless, the analytical gradient can be supplied and enhances the efficiency of the optimization process. Therefore, derivation of the cost function for MLE of K_D values and MLE of kinetic parameters is developed below.

Mathematical model for MLE of K_D values

We considered the simplified reaction:

$$\text{RISC} + \text{RNA} \leftrightharpoons \text{RISC:RNA}$$

For a given binding reaction j and a miRNA binding site i, the recovered concentration of site$_i$, xi,j, is a sum of si,j and nsi,j, which correspond to concentrations of miRISC-bound and non-specifically recovered site$_i$, respectively.

$$x_{i,j} = s_{i,j} + ns_{i,j} \quad \text{(Equation 11)}$$

Concentrations of miRISC-bound site$_i$, depends on dissociation constant K_D of site i:

$$K_{D_i} = \frac{[site_{i,j}]_{\text{free}} \times [\text{AGO:miRNA}_{\text{free}}]_j}{s_{i,j}} \quad \text{(Equation 12)}$$

where $[\text{AGO:miRNA}_{\text{free}}]_j$ is concentration of unbound miRISC in the reaction j and $[\text{site}_{i,j}]_{\text{free}}$ is the concentration of unbound site i in the binding reaction j.

Let $[\text{site}_i]_{\text{pool}}$ be the concentration of a binding site i in the RNA pool used in RBNS. After substituting $[site_{i,j}]_{\text{free}}$ by $[\text{site}_i]_{\text{pool}} - s_{i,j}$, Equation (12) becomes:

$$s_{i,j} = \frac{[\text{AGO:miRNA}_{\text{free}}]_j \times [\text{site}_i]_{\text{pool}}}{K_{D_i} + [\text{AGO:miRNA}_{\text{free}}]_j} \quad \text{(Equation 13)}$$

We describe concentration of non-specifically recovered site$_i$ by:

$$ns_{i,j} = c_j \times [\text{site}_{i,j}]_{\text{free}} \quad \text{(Equation 14)}$$

where c_j is a sample-specific proportionality constant and $[\text{site}_{i,j}]_{\text{free}}$ is the concentration of unbound site i in the binding reaction j.

We consider *background* to be the total concentration of all non-specifically recovered RNA, which is assumed to be the same in all binding reactions:

$$background = \sum_{i=1}^{m} ns_{i,j} = \sum_{i=1}^{m} c_j \times [\text{site}_{i,j}]_{\text{free}} = c_j \times \sum_{i=1}^{m} [\text{site}_{i,j}]_{\text{free}}$$

$$\text{Therefore,}\quad c_j = \frac{background}{\sum_{i=1}^{m} [\text{site}_{i,j}]_{\text{free}}} \quad \text{(Equation 15)}$$

After substituting si,j by expression Equation (13) and combining with Equations (14) and (15), xi,j can be expressed in terms of K_D of site i and background:

$$x_{i,j} = [\text{site}_i]_{\text{pool}} \times \left(\frac{[\text{AGO:miRNA}_{\text{free}}]_j}{K_{D_i} + [\text{AGO:miRNA}_{\text{free}}]_j}\right) \times \left(1 - \frac{background}{\sum_{k=1}^{m}[\text{site}_k]_{\text{pool}} - \sum_{k=1}^{m}\frac{[\text{AGO:miRNA}_{\text{free}}]_j \times [\text{site}_k]_{\text{pool}}}{K_{D_j} + [\text{AGO:miRNA}_{\text{free}}]_j}}\right) + \frac{background}{\sum_{k=1}^{m}[\text{site}_k]_{\text{pool}} - \sum_{k=1}^{m}\frac{[\text{AGO:miRNA}_{\text{free}}]_j \times [\text{site}_k]_{\text{pool}}}{K_{D_j} + [\text{AGO:miRNA}_{\text{free}}]_j}} \quad \text{(Equation 16)}$$

Equation (16) predicts concentrations of each binding site type, given information on RNA pool and a set of K_D values for each site type. We note that this equation also contains $[\text{AGO:miRNA}_{\text{free}}]_j$, concentration of unbound miRISC in a reaction j. $[\text{AGO:miRNA}_{\text{free}}]_j$ cannot be calculated explicitly, as it depends on concentration of miRISC bound to all site types. Therefore, $[\text{AGO:miRNA}_{\text{free}}]_j$ is approximated at each iteration of the optimization routine by solving the equation:

$$DF_j \times ago - [\text{AGO:miRNA}_{\text{free}}]_j - \sum_{i=1}^{m}\frac{[\text{AGO:miRNA}_{\text{free}}]_j \times [\text{site}_i]_{\text{pool}}}{K_{D_i} + [\text{AGO:miRNA}_{\text{free}}]_j} = 0 \quad \text{(Equation 17)}$$

To find root of Equation (17), *minimize_scalar* from a Python-based library SciPy is used within the interval (0, *ago*).

Parameters fitted during MLE of K_D values

The parameters to optimize includes K_D values of site_1, …, site_{m-1}, and no-site, as well as *background*, the total concentration of all non-specifically recovered RNA, and *ago*, the stock concentration of active AGO:miRNA complex. Upon optimization, some parameters may receive negative values, which is meaningless for affinities and concentrations. Therefore, we perform exponential transformation and define θ_1, …, θ_k, …, θ_m, θ_{m+1}, and θ_{m+2} as

$K_{D_k} = e^{\theta_k}$ for k in [1, m]

$ago = e^{\theta_{m+1}}$

$$background = e^{\theta_{m+2}} \quad \text{(Equation 18)}$$

Derivation of f_{cost} for MLE of K_D values

The function $f_{grad}(\theta)$ returns the derivative of f_{cost} with respect to each θ :

$$f_{grad}(\theta_1, \ \ldots, \ \theta_k, \ \ldots, \ \theta_{m+2}) = \left(\frac{df_{cost}}{d\theta_1}, \ \ldots, \ \frac{df_{cost}}{d\theta_k}, \ \ldots, \ \frac{df_{cost}}{d\theta_{m+2}}\right) \quad \text{(Equation 19)}$$

We derive $\frac{df_{cost}}{d\theta_k}$ using the chain rule:

$$\frac{df_{cost}}{d\theta_k} = \sum_{j=1}^{n}\sum_{i=1}^{m}\frac{\partial f_{cost}}{\partial x_{i,j}} \times \frac{\partial x_{i,j}}{\partial \theta_k} \quad \text{(Equation 20)}$$

First, to derive $\frac{\partial f_{cost}}{\partial x_{i,j}}$, substitute f_{cost} by Equation (10):

$$\frac{\partial f_{cost}}{\partial x_{i,j}} = \frac{\partial\left(\sum_{j=1}^{n}\left[\ln\left(\sum_{i=1}^{m}x_{i,j}\right) \times \sum_{i=1}^{m}reads_{i,j} - \sum_{i=1}^{m}(reads_{i,j} \times \ln(x_{i,j}))\right]\right)}{\partial x_{i,j}}$$

After using natural logarithm properties, the expression is simplified to:

$$\frac{\partial f_{cost}}{\partial x_{i,j}} = \frac{\sum_{z=1}^{m}reads_{z,j}}{\sum_{z=1}^{m}x_{z,j}} - \frac{reads_{i,j}}{x_{i,j}} \quad \text{(Equation 20.1)}$$

Next, to derive $\frac{df_{cost}}{d\theta_k}$, we note that xi,j contains si,j and *background*, which depend on θ_k. Therefore, we use the chain rule to write:

$$\frac{\partial x_{i,j}}{\partial \theta_k} = \frac{\partial x_{i,j}}{\partial\, background} \times \frac{\partial\, background}{\partial \theta_k} + \sum_{z=1}^{m}\left(\frac{\partial x_{i,j}}{\partial s_{z,j}} \times \frac{\partial s_{z,j}}{\partial \theta_k}\right) \quad \text{(Equation 20.2)}$$

To calculate the partial derivative of xi,j with respect to *background*, we substitute xi,j by Equation (11), and combining it with Equations (14) and (15) yields:

$$\frac{\partial x_{i,j}}{\partial\, background} = \frac{\partial\left(s_{i,j} + background \times \frac{[\text{site}_i]_{\text{pool}} - s_{i,j}}{\sum_{z=1}^{m}\left([\text{site}_z]_{\text{pool}} - s_{z,j}\right)}\right)}{\partial\, background} = \frac{[\text{site}_i]_{\text{pool}} - s_{i,j}}{\sum_{z=1}^{m}\left([\text{site}_z]_{\text{pool}} - s_{z,j}\right)} \quad \text{(Equation 20.2.1)}$$

To calculate the partial derivative of *background* with respect to θ_k, we note that *background* $= e^{\theta_{m+2}}$; therefore:

$$\frac{\partial\, background}{\partial\theta_k} = background \times \delta_{k(m+2)}, \quad \text{where } \delta_{k(m+2)} = \begin{cases} 1 \text{ if } k = m+2 \\ 0 \text{ if } k \neq m+2 \end{cases} \qquad \text{(Equation 20.2.2)}$$

To calculate the partial derivative of xi,j with respect to si,j, we substitute $x_{i,j}$ by Equation (11), and combining it with Equations (14) and (15) yields:

$$\frac{\partial x_{i,j}}{\partial s_{z,j}} = \frac{\partial\left(s_{i,j} \times \left(1 - \frac{background}{\sum_{k=1}^{m}\left([\text{site}_k]_{pool} - s_{k,j}\right)}\right) + \frac{background \times [\text{site}_i]_{pool}}{\sum_{k=1}^{m}\left([\text{site}_k]_{pool} - s_{k,j}\right)}\right)}{\partial s_{z,j}}$$

After using the composite function rule and rearranging, the partial derivative of $x_{i,j}$ with respect to si,j becomes:

$$\frac{\partial x_{i,j}}{\partial s_{z,j}} = \left(1 - \frac{background}{\sum_{k=1}^{m}\left([\text{site}_k]_{\text{pool}} - s_{k,j}\right)}\right) \times \delta_{zi} + background \times \frac{[\text{site}_k]_{\text{pool}} - s_{i,j}}{\left(\sum_{k=1}^{m}[\text{site}_k]_{\text{pool}} - \sum_{k=1}^{m} s_{k,j}\right)^2}, \quad \text{where } \delta_{zi} = \begin{cases} 1 & \text{if } z = i \\ 0 & \text{if } z \neq i \end{cases} \qquad \text{(Equation 20.2.3)}$$

By combining expressions of partial derivatives of $x_{i,j}$ with respect to background and si,j, expression Equation (20.2) becomes:

$$\frac{\partial x_{i,j}}{\partial\theta_k} = \frac{[\text{site}_i]_{\text{pool}} - s_{i,j}}{\sum_{z=1}^{m}\left([\text{site}_z]_{\text{pool}} - s_{z,j}\right)} \times background \times \delta_{k(m+2)}$$
$$+ \sum_{z=1}^{m}\left(\left[\left(1 - \frac{\text{background}}{\sum_{k=1}^{m}\left([\text{site}_k]_{\text{pool}} - s_{k,j}\right)}\right) \times \delta_{zi} + background \times \frac{[\text{site}_k]_{\text{pool}} - s_{i,j}}{\left(\sum_{k=1}^{m}[\text{site}_k]_{\text{pool}} - \sum_{k=1}^{m} s_{k,j}\right)^2}\right] \times \frac{\partial s_{z,j}}{\partial\theta_k}\right)$$

which can be further simplified to yield:

$$\frac{\partial x_{i,j}}{\partial\theta_k} = \frac{[\text{site}_i]_{\text{pool}} - s_{i,j}}{\sum_{z=1}^{m}\left([\text{site}_z]_{\text{pool}} - s_{z,j}\right)} \times background \times \delta_{k(m+2)}$$
$$+ background \times \frac{[\text{site}_k]_{\text{pool}} - s_{i,j}}{\left(\sum_{k=1}^{m}[\text{site}_k]_{\text{pool}} - \sum_{k=1}^{m} s_{k,j}\right)^2} \times \sum_{z=1}^{m}\frac{\partial s_{z,j}}{\partial\theta_k} \qquad \text{(Equation 20.3)}$$
$$+ \left(1 - \frac{background}{\sum_{k=1}^{m}[\text{site}_k]_{\text{pool}} - \sum_{k=1}^{m} s_{k,j}}\right) \times \frac{\partial s_{i,j}}{\partial\theta_k}$$

We note that $\frac{\partial x_{i,j}}{\partial\theta_k}$ requires expression of $\frac{\partial s_{i,j}}{\partial\theta_k}$. si,j is function of K_{D_i} and $[\text{AGO:miRNA}_{\text{free}}]_j$; therefore we apply the chain rule:

$$\frac{\partial s_{i,j}}{\partial\theta_k} = \frac{\partial s_{i,j}}{\partial K_{D_i}} \times \frac{\partial K_{D_i}}{\partial\theta_k} + \frac{\partial s_{i,j}}{\partial[\text{AGO:miRNA}_{\text{free}}]_j} \times \frac{\partial[\text{AGO:miRNA}_{\text{free}}]_j}{\partial\theta_k} \qquad \text{(Equation 20.4)}$$

To calculate the partial derivative of si,j with respect to K_D of site i, we substitute $s_{i,j}$ by Equation (13):

$$\frac{\partial s_{i,j}}{\partial K_{D_i}} = \frac{\partial\left(\frac{[\text{AGO:miRNA}_{\text{free}}]_j \times [\text{site}_i]_{\text{pool}}}{K_{D_i} + [\text{AGO:miRNA}_{\text{free}}]_j}\right)}{\partial K_{D_i}}$$

We use the composite function rule to write:

$$\frac{\partial s_{i,j}}{\partial K_{D_i}} = -\frac{[\text{AGO:miRNA}_{\text{free}}]_j \times [\text{site}_i]_{\text{pool}}}{\left(K_{D_i} + [\text{AGO:miRNA}_{\text{free}}]_j\right)^2} \qquad \text{(Equation 20.4.1)}$$

To calculate the partial derivative of K_{D_i} with respect to θ_k, we note that $K_{D_i} = e^{\theta_i}$ for i in [1, m]:

$$\frac{\partial K_{D_i}}{\partial\theta_k} = \frac{\partial e^{\theta_i}}{\partial\theta_k} = e^{\theta_i} \times \delta_{ki} = K_{D_i} \times \delta_{ki}, \quad \text{where } \delta_{ki} = \begin{cases} 1 & \text{if } k = i \\ 0 & \text{if } k \neq i \end{cases} \qquad \text{(Equation 20.4.2)}$$

To calculate the partial derivative of $s_{i,j}$ with respect to $[\text{AGO:miRNA}_{\text{free}}]_j$, we substitute $s_{i,j}$ by Equation (13):

$$\frac{\partial s_{i,j}}{\partial[\text{AGO:miRNA}_{\text{free}}]_j}=\frac{\partial}{\partial[\text{AGO:miRNA}_{\text{free}}]_j}\left(\frac{[\text{AGO:miRNA}_{\text{free}}]_j\times[\text{site}_i]_{\text{pool}}}{K_{D_i}+[\text{AGO:miRNA}_{\text{free}}]_j}\right)$$

After using the composite function rule and rearranging, the expression above becomes:

$$\frac{\partial s_{i,j}}{\partial[\text{AGO:miRNA}_{\text{free}}]_j}=\frac{K_{D_i}\times[\text{site}_i]_{\text{pool}}}{\left(K_{D_i}+[\text{AGO:miRNA}_{\text{free}}]_j\right)^2} \quad \text{(Equation 20.4.3)}$$

To calculate the partial derivative of $[\text{AGO:miRNA}_{\text{free}}]_j$ with respect to θ_k, we note that $[\text{AGO:miRNA}_{\text{free}}]_j = DF_j\times ago-\sum_{i=1}^{m}s_{i,j}$, where DF_j is the dilution factor of miRISC in the binding reaction j. Therefore, we can write:

$$\frac{\partial[\text{AGO:miRNA}_{\text{free}}]_j}{\partial\theta_k}=DF_j\times ago\times\delta_{k(m+1)}-\frac{\partial\left(\sum_{i=1}^{m}s_{i,j}\right)}{\partial\theta_k},\quad \text{where } \delta_{k(m+1)}=\begin{cases}1 & \text{if } k=m+1\\ 0 & \text{if } k\neq m+1\end{cases} \quad \text{(Equation 20.4.4.1)}$$

Use Equation (20.4) to calculate $\frac{\partial\left(\sum_{i=1}^{1}s_{i,j}\right)}{\partial\theta_k}$:

$$\frac{\partial\left(\sum_{i=1}^{m}s_{i,j}\right)}{\partial\theta_k}=\sum_{i=1}^{m}\left(\frac{\partial s_{i,j}}{\partial K_{D_i}}\times\frac{\partial K_{D_i}}{\partial\theta_k}\right)+\sum_{i=1}^{m}\frac{\partial s_{i,j}}{\partial[\text{AGO:miRNA}_{\text{free}}]_j}\times\frac{\partial[\text{AGO:miRNA}_{\text{free}}]_j}{\partial\theta_k} \quad \text{(Equation 20.4.4.2)}$$

Combining Equation (20.4.4.2) with Equation (20.4.4.1) and substituting $\frac{\partial s_{i,j}}{\partial K_{D_i}}$ by Equation (20.4.1), $\frac{\partial K_{D_i}}{\partial\theta_k}$ by Equation (20.4.2) and $\frac{\partial s_{i,j}}{[\text{AGO:miRNA}_{\text{free}}]_j}$ by Equation (20.4.3) yields after simplification:

$$\frac{\partial\left(\sum_{i=1}^{m}s_{i,j}\right)}{\partial\theta_k}=\frac{\sum_{i=1}^{m}\left(-\frac{[\text{AGO:miRNA}_{\text{free}}]_j\times[\text{site}_i]_{\text{pool}}}{\left(K_{D_i}+[\text{AGO:miRNA}_{\text{free}}]_j\right)^2}\times K_{D_i}\times\delta_{ki}\right)+\sum_{i=1}^{m}DF_j\times ago\times\delta_{k(m+1)}\times\frac{K_{D_i}\times[\text{site}_i]_{\text{pool}}}{\left(K_{D_i}+[\text{AGO:miRNA}_{\text{free}}]_j\right)^2}}{1+\sum_{i=1}^{m}\frac{K_{D_i}\times[\text{site}_i]_{\text{pool}}}{\left(K_{D_i}+[\text{AGO:miRNA}_{\text{free}}]_j\right)^2}}$$

(Equation 20.4.4.3)

We note that $\sum_{i=1}^{m}\left(-\frac{[\text{AGO:miRNA}_{\text{free}}]_j\times[\text{site}_i]_{\text{pool}}}{\left(K_{D_i}+[\text{AGO:miRNA}_{\text{free}}]_j\right)^2}\times K_{D_i}\times\delta_{ki}\right)$ can be simplified:

$$\sum_{i=1}^{m}\left(-\frac{[\text{AGO:miRNA}_{\text{free}}]_j\times[\text{site}_i]_{\text{pool}}}{\left(K_{D_i}+[\text{AGO:miRNA}_{\text{free}}]_j\right)^2}\times K_{D_i}\times\delta_{ki}\right)=-\frac{[\text{AGO:miRNA}_{\text{free}}]_j\times[\text{site}_i]_{\text{pool}}\times K_{D_i}}{\left(K_{D_i}+[\text{AGO:miRNA}_{\text{free}}]_j\right)^2}\times\mathbb{1}_{[1\ldots m]}(k),$$

where $\mathbb{1}_{[1\ldots m]}(k)=\begin{cases}1 & \text{if } k\in[1\ldots m]\\ 0 & \text{if } k\in[1\ldots m]\end{cases}$

Therefore, Equation (20.4.4.3) becomes:

$$\frac{\partial\left(\sum_{i=1}^{m}s_{i,j}\right)}{\partial\theta_k}=\frac{-\frac{[\text{AGO:miRNA}_{\text{free}}]_j\times[\text{site}_i]_{\text{pool}}\times K_{D_i}}{\left(K_{D_i}+[\text{AGO:miRNA}_{\text{free}}]_j\right)^2}\times\mathbb{1}_{[1\ldots m]}(k)+\sum_{i=1}^{m}DF_j\times ago\times\delta_{k(m+1)}\times\frac{K_{D_i}\times[\text{site}_i]_{\text{pool}}}{\left(K_{D_i}+[\text{AGO:miRNA}_{\text{free}}]_j\right)^2}}{1+\sum_{i=1}^{m}\frac{K_{D_i}\times[\text{site}_i]_{\text{pool}}}{\left(K_{D_i}+[\text{AGO:miRNA}_{\text{free}}]_j\right)^2}}$$

(Equation 20.4.4.4)

Finally, we can combine all the partial derivatives given by Equations (20.4.1), (20.4.2), (20.4.3), (20.4.4.1), and (20.4.4.4) to calculate $\frac{\partial s_{i,j}}{\partial\theta_k}$:

$$\frac{\partial s_{i,j}}{\partial\theta_k}=-\frac{[\text{AGO:miRNA}_{\text{free}}]_j\times[\text{site}_i]_{\text{pool}}}{\left(K_{D_i}+[\text{AGO:miRNA}_{\text{free}}]_j\right)^2}\times K_{D_i}\times\delta_{ki}+\frac{K_{D_i}\times[\text{site}_i]_{\text{pool}}}{\left(K_{D_i}+[\text{AGO:miRNA}_{\text{free}}]_j\right)^2}\times$$

$$\left[DF_j\times ago\times\delta_{k(m+1)}-\frac{-\frac{[\text{AGO:miRNA}_{\text{free}}]_j\times\times[\text{site}_i]_{\text{pool}}\times K_{D_i}}{\left(K_{D_i}+[\text{AGO:miRNA}_{\text{free}}]_j\right)^2}\times\mathbb{1}_{[1\ldots m]}(k)+\sum_{i=1}^{m}DF_j\times ago\times\delta_{k(m+1)}\times\frac{K_{D_i}\times[\text{site}_i]_{\text{pool}}}{\left(K_{D_i}+[\text{AGO:miRNA}_{\text{free}}]_j\right)^2}}{1+\sum_{i=1}^{m}\frac{K_{D_i}\times[\text{site}_i]_{\text{pool}}}{\left(K_{D_i}+[\text{AGO:miRNA}_{\text{free}}]_j\right)^2}}\right]$$

(Equation 20.4.4.5)

Equation (20.4.4.5) can be further re-arranged:

$$\frac{\partial s_{i,j}}{\partial \theta_k} = -\frac{[\text{AGO:miRNA}_{\text{free}}]_j \times [\text{site}_i]_{\text{pool}} \times K_{D_i}}{\left(K_{D_i} + [\text{AGO:miRNA}_{\text{free}}]_j\right)^2} \times \left(\delta_{ki} - \frac{K_{D_i} \times [\text{site}_i]_{\text{pool}}}{\left(K_{D_i} + [\text{AGO:miRNA}_{\text{free}}]_j\right)^2} \times \frac{\mathbb{I}_{[1\ldots m]}(k)}{1 + \sum_{i=1}^{m} \frac{K_{D_i} \times [\text{site}_i]_{\text{pool}}}{\left(K_{D_i} + [\text{AGO:miRNA}_{\text{free}}]_j\right)^2}}\right) + \frac{K_{D_i} \times [\text{site}_i]_{\text{pool}}}{\left(K_{D_i} + [\text{AGO:miRNA}_{\text{free}}]_j\right)^2} \times \frac{DF_j \times ago \times \delta_{k(m+1)}}{1 + \sum_{i=1}^{m} \frac{K_{D_i} \times [\text{site}_i]_{\text{pool}}}{\left(K_{D_i} + [\text{AGO:miRNA}_{\text{free}}]_j\right)^2}} \tag{Equation 20.5}$$

The gradient of f_{cost} can now be computed and is given by Equation (20):

$$\frac{df_{\text{cost}}}{d\theta_k} = \sum_{j=1}^{n} \sum_{i=1}^{m} \frac{\partial f_{\text{cost}}}{\partial x_{i,j}} \times \frac{\partial x_{i,j}}{\partial \theta_k}$$

Substituting $\frac{\partial f_{\text{cost}}}{\partial x_{i,j}}$ by Equation (20.1) and $\frac{\partial x_{i,j}}{\partial \theta_k}$ by Equation (20.3) yields:

$$\frac{df_{\text{cost}}}{d\theta_k} = \sum_{j=1}^{n} \sum_{i=1}^{m} \left(\frac{\sum_{z=1}^{m} reads_{z,j}}{\sum_{z=1}^{m} x_{z,j}} - \frac{reads_{i,j}}{x_{i,j}} \right) \times \left\{ \frac{[\text{site}_i]_{\text{pool}} - s_{i,j}}{\sum_{z=1}^{m} \left([\text{site}_z]_{\text{pool}} - \sum_{k=1}^{m} s_{z,j} \right)} \times background \times \delta_{k(m+2)} + background \times \frac{[\text{site}_k]_{\text{pool}} - s_{i,j}}{\sum_{k=1}^{m} \left([\text{site}_k]_{\text{pool}} - \sum_{k=1}^{m} s_{k,j} \right)^2} \times \sum_{z=1}^{m} \frac{\partial s_{z,j}}{\partial \theta_k} + \left(1 - \frac{background}{\sum_{k=1}^{m} [\text{site}_k]_{\text{pool}} - \sum_{k=1}^{m} s_{k,j}} \right) \times \frac{\partial s_{i,j}}{\partial \theta_k} \right\}$$

where $\frac{\partial\left(\sum_{i=1}^{m} s_{i,j}\right)}{\partial \theta_k}$ is given by Equation (20.4.4.4) and $\frac{\partial s_{i,j}}{\partial \theta_k}$ is given by Equation (20.5).

Initial guess for MLE of K_D values and calculation of 95% cIs

Bootstrapping of 95% of the data was performed ten times on sequencing reads from each binding reaction and the RNA pool. MLE of K_D values was performed on each bootstrapped sample by using 100 different combinations of 10 initial guesses of miRISC concentration (in the range 0.5–25 nM) and 10 initial guesses of K_D for RNA with no enriched site (in the range 0.5–10 nM). K_D values were initialized as the inverse of the average enrichment values. The background was initialized at 0.1 nM. All the initial guesses were partially randomized by adding a value drawn from a normal distribution with mean 0 and standard deviation 0.1. The cost function was evaluated in the presence of physically meaningful constraints on the parameters: 0.1 pM $\le K_D^{\text{site}} \le$ 100 nM, 100 pM $\le K_D^{\text{no-site}}$ $\le$ 10,000 nM, 100 pM $\le$ *ago* $\le$ 100 nM, and 5 pM $\le$ *background* $\le$ 5 nM. Any of the fitted parameters were at the boundaries at the end of the optimization routine. K_D estimates, the background, and the stock concentration of miRISC provided by MLE were used to predict counts of each binding site type in sequencing data. These counts were compared with observed sequencing data, and MLE results were retained if Pearson correlation coefficient was >0.90. Results from independent starting points satisfying this criterion were combined. All bootstrapped samples were combined. Finally, estimates from two independent RBNS assays were merged. Median and 95% confidence intervals on medians were reported.

Mathematical model for MLE of k_{on} and k_{off} values

We considered the simplified reaction:

$$\text{RISC} + \text{RNA} \underset{k_{\text{off}}}{\overset{k_{\text{on}}}{\rightleftharpoons}} \text{RISC:RNA}$$

For a binding reaction at time *t* and miRNA binding site *i*, the recovered concentration of site$_i$ $x_{i,t}$ is a sum of miRISC-bound site$_i$, si,t, and non-specifically recovered site$_i$. Similar to how Equation (11) was obtained above to estimate K_D, xi,t is described by

$$x_{i,t} = s_{i,t} \times \left(1 - \frac{background}{\sum_{k=1}^{m} \left([\text{site}_k]_{\text{pool}} - s_{k,t} \right)} \right) + \frac{background \times [\text{site}_i]_{\text{pool}}}{\sum_{k=1}^{m} \left([\text{site}_k]_{\text{pool}} - s_{k,t} \right)} \tag{Equation 21}$$

where si,t is given by

$$\frac{ds_i}{dt} = k_{on_i} \times [\text{site}_i]_{free} \times [\text{AGO:miRNA}_{free}] - k_{off_i} \times s_i \qquad \text{(Equation 22)}$$

As $[\text{site}_i]_{free} = [\text{site}_i]_{pool} - s_i$, Equation (22) becomes:

$$\frac{ds_i}{dt} + s_i \times \left(k_{on_i} \times [\text{AGO:miRNA}_{free}] + k_{off_i}\right) = k_{on_i} \times [\text{site}_i]_{pool} \times [\text{AGO:miRNA}_{free}] \qquad \text{(Equation 23)}$$

This is a linear differential equation with constant coefficient of the type:
$y'(x)+ay(x)=b$, whose solution is $y(x) = \frac{b}{a} + ce^{-ax}$.
Therefore,

$$s_{i,t} = \frac{k_{on_i} \times [\text{site}_i]_{pool} \times [\text{AGO:miRNA}_{free}]_t}{k_{on_i} \times [\text{AGO:miRNA}_{free}]_t + k_{off_i}} + c \times e^{-\left(k_{on_i} \times [\text{AGO:miRNA}_{free}]_t + k_{off_i}\right)^t}$$

To define c, we consider initial conditions. There is no complex formation at $t = 0$, i.e., $s_{i,0}=0$ and $[\text{AGO:miRNA}_{free}]_0 = [\text{AGO:miRNA}]_{total}$.
Therefore,

$$c = -\frac{k_{on_i} \times [\text{site}_i]_{pool} \times [\text{AGO:miRNA}]_{total}}{k_{on_i} \times [\text{AGO:miRNA}]_{total} + k_{off_i}}$$

and

$$s_{i,t} = -\frac{k_{on_i} \times [\text{site}_i]_{pool} \times [\text{AGO:miRNA}_{free}]_t}{k_{on_i} \times [\text{AGO:miRNA}_{free}]_t + k_{off_i}} - \frac{k_{on_i} \times [\text{site}_i]_{pool} \times [\text{AGO:miRNA}]_{total}}{k_{on_i} \times [\text{AGO:miRNA}]_{total} + k_{off_i}} \times e^{-\left(k_{on_i} \times [\text{AGO:miRNA}_{free}]_t + k_{off_i}\right)^t} \qquad \text{(Equation 24)}$$

After substituting si,t by Equation (24), the Equation (21) becomes:

$$x_{i,t} = \left(\frac{k_{on_i} \times [\text{site}_i]_{pool} \times [\text{AGO:miRNA}_{free}]_t}{k_{on_i} \times [\text{AGO:miRNA}_{free}]_t + k_{off_i}} - \frac{k_{on_i} \times [\text{site}_i]_{pool} \times [\text{AGO:miRNA}]_{total}}{k_{on_i} \times [\text{AGO:miRNA}]_{total} + k_{off_i}} \times e^{-\left(k_{on_i} \times [\text{AGO:miRNA}_{free}]_t + k_{off_i}\right)^t}\right) \times$$

$$\left(1 - \frac{background}{\sum_{k=1}^{m} [\text{site}_k]_{pool} - \sum_{k=1}^{m}\left(\frac{k_{on_k} \times [\text{site}_k]_{pool} \times [\text{AGO:miRNA}_{free}]_t}{k_{on_k} \times [\text{AGO:miRNA}_{free}]_t + k_{off_k}} - \frac{k_{on_k} \times [\text{site}_k]_{pool} \times [\text{AGO:miRNA}]_{total}}{k_{on_k} \times [\text{AGO:miRNA}]_{total} + k_{off_k}} \times e^{-\left(k_{on_k} \times [\text{AGO:miRNA}_{free}]_t + k_{off_k}\right)^t}\right)}\right) +$$

$$\frac{background \times [\text{site}_i]_{pool}}{\sum_{k=1}^{m} [\text{site}_k]_{pool} - \sum_{k=1}^{m}\left(\frac{k_{on_k} \times [\text{site}_k]_{pool} \times [\text{AGO:miRNA}_{free}]_t}{k_{on_k} \times [\text{AGO:miRNA}_{free}]_t + k_{off_k}} - \frac{k_{on_k} \times [\text{site}_k]_{pool} \times [\text{AGO:miRNA}]_{total}}{k_{on_k} \times [\text{AGO:miRNA}]_{total} + k_{off_k}} \times e^{-\left(k_{on_k} \times [\text{AGO:miRNA}_{free}]_t + k_{off_k}\right)^t}\right)}$$

(Equation 25)

Equation (25) predicts concentrations of each binding site type at time t, given information on RNA pool and a set of k_{on} and k_{off} values for each site type. Our typical binding experiment consists of 10 time points and measures concentrations of 10 binding sites, yielding a system of 100 $x_{i,t}$ equations in total. Some of these equations are likely linearly dependent on others; but we reasoned that there should remain enough independent equations to estimate 21 variables (k_{on} and k_{off} values of 10 sites, and the *background*). Therefore, we do not impose any additional constrains.

We also note that the Equation (25) contains $[\text{AGO:miRNA}_{free}]_t$, concentration of unbound miRISC at time t. $[\text{AGO:miRNA}_{free}]_t$ cannot be calculated explicitly, as it depends on the concentration of miRISC bound to all site types. Therefore, $[\text{AGO:miRNA}_{free}]_t$ is approximated at each iteration of the optimization routine by solving the equation:

$$[\text{AGO:miRNA}]_{total} - [\text{AGO:miRNA}_{free}]_t - \sum_{i=1}^{m}\left(\frac{k_{on_i} \times [\text{site}_i]_{pool} \times [\text{AGO:miRNA}_{free}]_t}{k_{on_i} \times [\text{AGO:miRNA}_{free}]_t + k_{off_i}} - \frac{k_{on_i} \times [\text{site}_i]_{pool} \times [\text{AGO:miRNA}]_{total}}{k_{on_i} \times [\text{AGO:miRNA}]_{total} + k_{off_i}} \times e^{-\left(k_{on_i} \times [\text{AGO:miRNA}_{free}]_t + k_{off_i}\right)^t}\right) = 0$$

(Equation 26)

To find root of Equation (26), *minimize_scalar* from a Python-based library SciPy is used within the interval (0, $[\text{AGO:miRNA}]_{total}$). In the current version of implementation, $[\text{AGO:miRNA}]_{total}$ is supplied by the user, not fit.

Parameters fitted during MLE of k_{on} and k_{off} values

The set of parameters to optimize contains k_{on} and k_{off} values of site$_1$, …, site$_{m-1}$, and no-site, as well as *background*, the total concentration of all non-specifically recovered RNA. Upon optimization, some parameters may receive negative values, which is

meaningless for kinetic rate constants and concentrations. Therefore, we perform exponential transformation and define θ_1, …, θ_m, …, θ_{2m}, and θ_{2m+1} as

$k_{on_k} = e^{\theta_k}$ for k in [1, m]

$k_{off_k} = e^{\theta_{m+k}}$ for k in [1, m]

$$background = e^{\theta_{2m+1}} \quad \text{(Equation 27)}$$

Derivation of f_{cost} for MLE of k_{on} and k_{off} values

The function $f_{grad}\theta$ returns the derivative of f_{cost} with respect to each θ :

$$f_{grad}(\theta_1, \ \ldots, \theta_{2m+1}) = \left(\frac{df_{cost}}{d\theta_1}, \ \ldots, \frac{df_{cost}}{d\theta_{2m+1}}\right) \quad \text{(Equation 28)}$$

We derive $\frac{df_{cost}}{d\theta_k}$ using the chain rule:

$$\frac{df_{cost}}{d\theta_k} = \sum_{t=1}^{n}\sum_{i=1}^{m}\frac{\partial f_{cost}}{\partial x_{i,t}} \times \frac{\partial x_{i,t}}{\partial \theta_k} \quad \text{(Equation 29)}$$

As demonstrated above, $\frac{\partial f_{cost}}{\partial x_{i,t}}$ is given by the Equation (20.1) and $\frac{\partial x_{i,t}}{\partial \theta_k}$ is given by the Equation (20.3). The Equation (20.3) contains $\frac{\partial s_{i,t}}{\partial \theta_k}$ and $\sum_{z=1}^{m}\frac{\partial s_{z,j}}{\partial \theta_k}$ whose expressions differ from RBNS experiments at equilibrium, and therefore should be calculated.

si,t contains k_{on_i}, k_{off_i}, and $[\text{AGO:miRNA}_{free}]_t$ that depend on θ_k, so partial derivatives are required. We use the chain rule to write:

$$\frac{\partial s_{i,t}}{\partial \theta_k} = \frac{\partial s_{i,t}}{\partial k_{on_i}} \times \frac{\partial k_{on_i}}{\partial \theta_k} + \frac{\partial s_{i,t}}{\partial k_{off_i}} \times \frac{\partial k_{off_i}}{\partial \theta_k} + \frac{\partial s_{i,t}}{\partial [\text{AGO:miRNA}_{free}]_t} \times \frac{\partial [\text{AGO:miRNA}_{free}]_t}{\partial \theta_k} \quad \text{(Equation 30)}$$

To calculate the partial derivative of si,j with respect to k_{on_i}, we use the expression of si,j from Equation (24). After applying the quotient rule and some rearrangements, we obtain:

$$\frac{\partial s_{i,t}}{\partial k_{on_i}} = \frac{[\text{site}_i]_{pool} \times [\text{AGO:miRNA}_{free}]_t \times k_{off_i}}{\left(k_{on_i} \times [\text{AGO:miRNA}_{free}]_t + k_{off_i}\right)^2} - \frac{[\text{site}_i]_{pool} \times [\text{AGO:miRNA}]_{total}}{k_{on_i} \times [\text{AGO:miRNA}]_{total} + k_{off_i}} \times e^{-\left(k_{on_i} \times [\text{AGO:miRNA}_{free}]_t + k_{off_i}\right)^t} \times \left(\frac{k_{off_i}}{k_{on_i} \times [\text{AGO:miRNA}]_{total} + k_{off_i}} - [\text{AGO:miRNA}_{free}]_t \times t \times k_{on_i}\right) \quad \text{(Equation 30.1)}$$

To calculate the partial derivative of k_{on_i} with respect to θ_k, we note that $k_{on_k} = e^{\theta_k}$ for k in [1, m]:

$$\frac{\partial k_{on_i}}{\partial \theta_k} = \frac{\partial e^{\theta_i}}{\partial \theta_k} = e^{\theta_i} \times \delta_{ki} = k_{on_i} \times \delta_{ki}, \quad \text{where} \quad \delta_{ki} = \begin{cases} 1 & \text{if } k = i \\ 0 & \text{if } k \neq i \end{cases} \quad \text{(Equation 30.2)}$$

To calculate the partial derivative of si,j with respect to k_{off_i}, we use the expression of si,j from Equation (24). After applying the quotient rule and some rearrangements, we obtain:

$$\frac{\partial s_{i,t}}{\partial k_{off_i}} = -\frac{k_{on_i} \times [\text{site}_i]_{pool} \times [\text{AGO:miRNA}_{free}]_t}{\left(k_{on_i} \times [\text{AGO:miRNA}_{free}]_t + k_{off_i}\right)^2} + \frac{k_{on_i} \times [\text{site}_i]_{pool} \times [\text{AGO:miRNA}]_{total}}{\left(k_{on_i} \times [\text{AGO:miRNA}_{free}]_t + k_{off_i}\right)^2} \times e^{-\left(k_{on_i} \times [\text{AGO:miRNA}_{free}]_t + k_{off_i}\right)^t} \times \left(t + \frac{1}{k_{on_i} \times [\text{AGO:miRNA}]_{total} + k_{off_i}}\right) \quad \text{(Equation 30.3)}$$

To calculate the partial derivative of k_{off_i} with respect to θ_k, we note that $k_{off_k} = e^{\theta_{m+k}}$ for k in [1, m]:

$$\frac{\partial k_{off_i}}{\partial \theta_k} = \frac{\partial e^{\theta(m+i)}}{\partial \theta_k} = e^{\theta(m+i)} \times \delta_{k(m+i)} = k_{off_i} \times \delta_{k(m+i)}, \quad \text{where} \quad \delta_{k(m+i)} = \begin{cases} 1 & \text{if } k = m+i \\ 0 & \text{if } k \neq m+i \end{cases} \quad \text{(Equation 30.4)}$$

To calculate the partial derivative of si,j with respect to $[\text{AGO:miRNA}_{free}]_t$, we use the expression of si,j from Equation (24). After applying the quotient rule and some rearrangements, we obtain:

$$\frac{\partial s_{i,t}}{\partial [\text{AGO:miRNA}_{free}]_t} = \frac{k_{on_i} \times [\text{site}_i]_{pool} \times k_{off_i}}{\left(k_{on_i} \times [\text{AGO:miRNA}_{free}]_t + k_{off_i}\right)^2} + \frac{k_{on_i} \times [\text{site}_i]_{pool} \times [\text{AGO:miRNA}]_{total} k_{on_i} \times t}{k_{on_i} \times [\text{AGO:miRNA}]_{total} + k_{off_i}} \times e^{-\left(k_{on_i} \times [\text{AGO:miRNA}_{free}]_t + k_{off_i}\right)^t} \quad \text{(Equation 30.5)}$$

To calculate the partial derivative of $[\text{AGO:miRNA}_{\text{free}}]_t$ with respect to θ_k, we note that

$$\frac{\partial[\text{AGO:miRNA}_{\text{free}}]_t}{\partial\theta_k} = \frac{\partial\left([\text{AGO:miRNA}]_{\text{total}} - \sum_{i=1}^{m} s_{i,t}\right)}{\partial\theta_k} = -\frac{\partial\sum_{i=1}^{m} s_{i,t}}{\partial\theta_k}$$

Substituting $\frac{\partial s_{i,t}}{\partial\theta_k}$ by Equation (30) and simplifying the expression yields:

$$\frac{\partial[\text{AGO:miRNA}_{\text{free}}]_t}{\partial\theta_k} = -\frac{\sum_{i=1}^{m}\left(\frac{\partial s_{i,t}}{\partial k_{\text{on}_i}} \times \frac{\partial k_{\text{on}_i}}{\partial\theta_k}\right) + \sum_{i=1}^{m}\left(\frac{\partial s_{i,t}}{\partial k_{\text{off}_i}} \times \frac{\partial k_{\text{off}_i}}{\partial\theta_k}\right)}{1 + \sum_{i=1}^{m}\frac{\partial s_{i,t}}{\partial[\text{AGO:miRNA}_{\text{free}}]_t}}$$

Some terms can be simplified further:

$$\sum_{i=1}^{m}\left(\frac{\partial s_{i,t}}{\partial k_{\text{on}_i}} \times \frac{\partial k_{\text{on}_i}}{\partial\theta_k}\right) = \sum_{i=1}^{m}\left(\frac{\partial s_{i,t}}{\partial k_{\text{on}_i}} \times k_{\text{on}_i} \times \delta_{ki}\right) = \frac{\partial s_{i,t}}{\partial k_{\text{on}_i}} \times k_{\text{on}_i} \times \mathbb{1}_{[1\ldots m]}(k),\ \text{where } \mathbb{1}_{[1\ldots m]}(k) = \begin{cases} 1 \text{ if } k \in [1\ldots m] \\ 0 \text{ if } k \in [1\ldots m] \end{cases}$$

$$\sum_{i=1}^{m}\left(\frac{\partial s_{i,t}}{\partial k_{\text{off}_i}} \times \frac{\partial k_{\text{off}_i}}{\partial\theta_k}\right) = \sum_{i=1}^{m}\left(\frac{\partial s_{i,t}}{\partial k_{\text{off}_i}} \times k_{\text{off}_i} \times \delta_{k(m+i)}\right) = \frac{\partial s_{i,t}}{\partial k_{\text{off}_i}} \times k_{\text{off}_i} \times \mathbb{1}_{[m+1\ldots 2m]}\left(k\right),\ \text{where}$$

$$\mathbb{1}_{[m+1\ldots 2m]}\left(k\right) = \begin{cases} 1 \text{ if } k \in [m+1\ldots 2m] \\ 0 \text{ if } k \in [m+1\ldots 2m] \end{cases}$$

After simplifying, we obtain:

$$\frac{\partial[\text{AGO:miRNA}_{\text{free}}]_t}{\partial\theta_k} = -\frac{\frac{\partial s_{i,t}}{\partial k_{\text{on}_i}} \times k_{\text{on}_i} \times \mathbb{1}_{[1\ldots m]}(k) + \frac{\partial s_{i,t}}{\partial k_{\text{off}_i}} \times k_{\text{off}_i} \times \mathbb{1}_{[m+1\ldots 2m]}(k)}{1 + \sum_{i=1}^{m}\frac{\partial s_{i,t}}{\partial[\text{AGO:miRNA}_{\text{free}}]_t}} \quad \text{(Equation 30.6)}$$

The gradient of f_{cost} can now be computed.
Because of the complexity of the equations, the full solution of $\frac{df_{\text{cost}}}{d\theta_k}$ is not shown.
$\frac{df_{\text{cost}}}{d\theta_k}$ is given by Equation (29), where $\frac{\partial x_{i,t}}{\partial\theta_k}$ is substituted by Equation (20.3), $\frac{\partial s_{i,t}}{\partial\theta_k}$ by Equation (30), $\frac{\partial s_{i,t}}{\partial k_{\text{on}_i}} \times \frac{\partial k_{\text{on}_i}}{\partial\theta_k}$ by Equations (30.1) and (30.2), $\frac{\partial s_{i,t}}{\partial k_{\text{off}_i}} \times \frac{\partial k_{\text{off}_i}}{\partial\theta_k}$ by Equations (30.3) and (30.4), $\frac{\partial s_{i,t}}{\partial[\text{AGO:miRNA}_{\text{free}}]_t}$ by Equation (30.5), and $\frac{\partial[\text{AGO:miRNA}_{\text{free}}]_t}{\partial\theta_k}$ by Equation (30.6).

Initial guess for MLE of k_{on} and k_{off} values and calculation of 95% cIs

Bootstrapping of 95% of the data was performed ten times on sequencing reads from each time point and the RNA pool. MLE was performed on each bootstrapped sample 100 times. The k_{on} values of all sites were initialized as $\frac{Enrichment_{site}^{30\text{ s}} - Enrichment_{site}^{0\text{ s}}}{(t_{30\text{ s}} - t_{0\text{ s}}) \times [RISC]_{total}}$. The k_{off} values of binding sites were initialized as the inverse of the average enrichment values, and the k_{off} for no-site was set to 100 s^{-1}. The background was initialized at 0.1 nM. All the initial guesses were partially randomized by adding a value drawn from a normal distribution with mean 0 and standard deviation 0.001 (except that standard deviation 1 or 0.1 was used for $k_{\text{off}}{}^{\text{no-site}}$ and the background, respectively). The cost function was evaluated in the presence of physically meaningful constraints on the parameters: $10^5\ M^{-1}\ s^{-1} \leq k_{\text{on}}{}^{\text{all}} \leq 10^9\ M^{-1}\ s^{-1}$, $0.0005\ s^{-1} \leq k_{\text{off}}{}^{\text{site}} \leq 20\ s^{-1}$, $0.0005\ s^{-1} \leq k_{\text{off}}{}^{\text{no-site}} \leq 200\ s^{-1}$, and 5 pM $\leq$ *background* $\leq$ 10 nM. None of the fitted parameters were at the boundaries at the end of the optimization routine. The estimates provided by MLE were used to predict counts of each binding site type in sequencing data. These counts were compared with observed sequencing data, and MLE results were retained if Pearson correlation coefficient was >0.90. Results from independent starting points satisfying this criterion were combined. All bootstrapped samples were combined. Finally, estimates from two independent RBNS assays were merged. Median and 95% confidence intervals on medians were reported.

Cell Reports Methods, Volume *2*

Supplemental information

Principles and pitfalls of high-throughput analysis of microRNA-binding thermodynamics and kinetics by RNA Bind-n-Seq

Karina Jouravleva, Joel Vega-Badillo, and Phillip D. Zamore

Table S1. Concentration of de novo identified binding sites in the RNA input pool, Related to STAR Methods

Concentrations of lsy-6, miR-1, miR-7, miR-124, miR-155, and let-7a^{hsa}, and let-7a^{mmu}, miR-34b and miR-449a sites were calculated in datasets generated in McGeary et al. (2019) and in this study, respectively.

lsy-6 sites	Concentration (nM)
8mer	0.071
7mer-m8	0.216
7mer-A1	0.141
6mer	0.571
6mer-m8	0.791
6mer-A1	0.699
5mer-m2.6	2.34
8mer-w8	0.053
8mer-b5.6A	0.015
8mer-b6.7G	0.014
8mer-x3U	0.067
8mer-x7A	0.066
10mer-m11.20	0.002

miR-1 sites	Concentration (nM)
8mer	0.069
7mer-m8	0.206
7mer-A1	0.221
6mer	0.656
6mer-m8	0.748
6mer-A1	0.637
5mer-m2.6	1.92
5mer-A1	2.83
8mer-w7	0.065
7mer-w7	0.185
8mer-b3.4U	0.019
8mer-b5.6A	0.017

miR-7 sites	Concentration (nM)
8mer	0.039
7mer-m8	0.103
7mer-A1	0.101
6mer	0.557
6mer-A1	0.506
5mer-m2.6	1.56
8mer-x7G	0.039
8mer-b6.7U	0.008
8mer-b6.7G	0.011
8mer-b7.8A	0.104

miR-124 sites	Concentration (nM)
8mer	0.042
7mer-m8	0.152
7mer-A1	0.139
6mer	0.521
6mer-m8	0.559
6mer-A1	0.594
5mer-m2.6	2.12
8mer-w5	0.038
AA-4mer-m5.8	0.606
8mer-b3.4A	0.011
8mer-b3.4C	0.011
8mer-b4.5A	0.011
8mer-b5.6U	0.012
8mer-b5.6G	0.011
8mer-b6.7A	0.011
8mer-b6.7U	0.011
A-7mer-b4.5A-w5	0.011
AA-7mer-b4.5U	0.002
AA-7mer-b5.6A	0.003
AA-7mer-b5.6U	0.002
10mer-m9.18	0.003
10mer-m10.19	0.002
10mer-m11.20	0.003
9mer-m11.19	0.009

miR-155 sites	Concentration (nM)
8mer	0.062
7mer-m8	0.169
7mer-A1	0.173
6mer	0.497
6mer-m8	0.754
8mer-w6	0.046
8mer-x5U	0.053
7mer-A1-x5U	0.015
10mer-m11.20	0.003
10mer-m12.21	0.002
10mer-m13.22	0.002
10mer-m13.22-w17	0.003
9mer-m13.21	0.009
10mer-m14.23	0.003
9mer-m14.22	0.008

let-7a^{hsa} sites	Concentration (nM)
8mer	0.071
7mer-m8	0.197
7mer-A1	0.121
6mer	0.331
6mer-m8	0.878
6mer-A1	0.716
5mer-m2.6	2.03
5mer-A1	3.16
8mer-w8	0.067
7mer-w8	0.182
8mer-b5.6A	0.018

let-7a^{mmu} sites	Concentration (nM)
8mer	0.039
7mer-m8	0.106
7mer-A1	0.049
6mer	0.136
6mer-m8	0.574
6mer-A1	0.335
5mer-m2.6	0.935
5mer-A1	2.06
8merw8	0.027
7mer-w8	0.077
8mer-w6	0.046
7mer-w6	0.127
8mer-b5.6A	0.009
10mer-m11.20	0.002
10mer-m12.21	0.001

miR-34b and miR-449a sites	Concentration (nM)
8mer	0.056
7mer-m8	0.149
7mer-A1	0.057
6mer	0.156
6mer-m8	0.645
6mer-A1	0.623
5mer-m2.6	1.78
8mer-w8	0.035
7mer-w8	0.098
8mer-w7	0.062

Table S2. RNA and DNA Oligos Used in This Study, Related to STAR Methods

RISC loading	Sequence<br>Seed; m indicates 2′-O-methyl ribose; p indicates 5′ monophosphate
Guide strand for unlabeled let-7a RISC	pUGA GGU AGU AGG UUG UAU AGU
Guide strand for Alexa let-7a RISC	pUGA GGU AGU AGG UUG UAU AGU-NH_2
Guide strand for miR-449a RISC	pUGG CAG UGU AUU GUU AGC UGG U
Guide strand for miR-34b RISC	pAGG CAG UGU AAU UAG CUG AUU GU
**RISC purification**	**Sequence**<br>RNA, DNA; m, 2′-O-methyl; p, 5′ phosphate; Bio, Biotin-6-carbon spacer
Capture Oligo to affinity purify let-7a RISC	Bio-mAmUmA mGmAmC mUmGmC mGmAmC mAmAmU mAmGmC mCmUmA mCmCmU mCmCmG mAmAmC mG
DNA competitor to elute let-7a RISC	CGT TCG GAG GTA GGC TAT TGT CGC AGT CTA T-Bio
Capture Oligo to affinity purify miR-449a RISC	Bio-mGmAmU mCmAmA mCmAmA mUmAmA mCmCmC mAmCmC mAmCmU mGmCmC mUmAmU mAmGmA
DNA competitor to elute miR-449a RISC	Bio-TTA TAG GCA GTG GTG GGT TAT TGT TGA TC
Capture Oligo to affinity purify miR-34b RISC	Bio-mGmAmU mAmUmU mCmAmA mGmCmU mAmCmC mAmCmC mAmCmU mGmCmC mUmAmU mAmA
DNA competitor to elute miR-34b RISC	Bio-TTA TAG GCA GTG GTG GTA GCT TGA ATA TC
**RISC quantification**	**Sequence**<br>RNA, DNA; p, 5′ phosphate
DNA probe to quantify total concentration of let-7a RISC by Northern Blot	GAT ACT ATA CAA CCT ACT ACC TCA ACC T
DNA probe to quantify total concentration of miR-449a RISC by Northern Blot	ATG ACC AGC TAA CAA TAC ACT GCC AAC T
DNA probe to quantify total concentration of miR-34b RISC by Northern Blot	ATG ACA ATC AGC TAA TTA CAC TGC CTA CT

RNA target to quantify active concentration of let-7a RISC by double-filter binding	pAAA AUG AUA ACA AGG AUC UAC CUC AAA A
RNA target to quantify active concentration of miR-449a and miR-34b RISCs by double-filter binding	pAUG AAA UCG AUA UCU AUC ACU GCC AAC U
**CoSMoS**	**Sequence**<br>RNA, DNA; Bio, Biotin-6-carbon spacer; target region; U, Alexa Fluor 647 deoxyuridine
Klenow polymerase template to synthesize 3′ DNA extension containing 17 Alexa Fluor 647 dyes for let-7a RNA with t10–t19 site adjacent to t3–t6 site	ATT GTT GTT ATT GTT GTT ATT GTT GTT ATT GTT GTT ATT GTT GTT ATT GTT<br>GTT ATT GTT GTT ATT GTT GTT ATT GTT GTT ATT GTT GTT ATT GTT GTT ATT<br>GTT GTT ATT GTT GTT ATT GTT GTT ATT GTT GTT ATT GTT GTT ATT TAC ATC<br>TAG GTC TTA GTC
Trap oligonucleotide for the preceding template (fully complementary)	GAC TAA GAC CTA GAT GTA AAT AAC AAC AAT AAC AAC AAT AAC AAC AAT AAC<br>AAC AAT AAC AAC AAT AAC AAC AAT AAC AAC AAT AAC AAC AAT AAC AAC AAT<br>AAC AAC AAT AAC AAC AAT AAC AAC AAT AAC AAC AAT AAC AAC AAT AAC AAC<br>AAT AAC AAC AAT
5′-tethered, RNA target with let-7a t10–t19 site adjacent to t3–t6 site and a 3′ DNA extension containing 17 Alexa Fluor 647 dyes	Bio-AUU AAA UAU ACA ACC UGA CUA AGA CCU AGA UGU AAA UAA CAA CAA UAA<br>CAA CAA UAA CAA CAA UAA CAA CAA UAA CAA CAA UAA CAA CAA UAA CAA CAA<br>UAA CAA CAA UAA CAA CAA UAA CAA CAA UAA CAA CAA UAA CAA CAA UAA CAA<br>CAA UAA CAA CAA UAA CAA CAA UAA CAA CAA U
Klenow polymerase template to synthesize 3′ DNA extension containing 17 Alexa Fluor 647 dyes for let-7a RNA with 6mer site	ATT GTT GTT ATT GTT GTT ATT GTT GTT ATT GTT GTT ATT GTT GTT ATT GTT<br>GTT ATT GTT GTT ATT GTT GTT ATT GTT GTT ATT GTT GTT ATT GTT GTT ATT<br>GTT GTT ATT GTT GTT ATT GTT GTT ATT GTT GTT ATT GTT GTT ATT TAC ATC<br>TAG AGA GGT ATA
Trap oligonucleotide for the preceding template (fully complementary)	TAT ACC TCT CTA GAT GTA AAT AAC AAC AAT AAC AAC AAT AAC AAC AAT AAC<br>AAC AAT AAC AAC AAT AAC AAC AAT AAC AAC AAT AAC AAC AAT AAC AAC AAT<br>AAC AAC AAT AAC AAC AAT AAC AAC AAT AAC AAC AAT AAC AAC AAT AAC AAC<br>AAT AAC AAC AAT
5′-tethered, RNA target with let-7a 6mer site and a 3′ DNA extension containing 17 Alexa Fluor 647 dyes	Bio-AUU AAA UAA UGG AGA AUU AAA UAU ACC UCU CUA GAU GUA AAU AAC AAC<br>AAU AAC AAC AAU AAC AAC AAU AAC AAC AAU AAC AAC AAU AAC AAC AAU AAC<br>AAC AAU AAC AAC AAU AAC AAC AAU AAC AAC AAU AAC AAC AAU AAC AAC AAU<br>AAC AAC AAU AAC AAC AAU AAC AAC AAU AAC AAC AAU
Klenow polymerase template to synthesize 3′ DNA extension containing 17 Alexa Fluor 647 dyes for other let-7a RNA targets	ATT GTT GTT ATT GTT GTT ATT GTT GTT ATT GTT GTT ATT GTT GTT ATT GTT<br>GTT ATT GTT GTT ATT GTT GTT ATT GTT GTT ATT GTT GTT ATT GTT GTT ATT<br>GTT GTT ATT GTT GTT ATT GTT GTT ATT GTT GTT ATT GTT GTT ATT TAC ATC<br>TTC GTC TTA GTC

Trap oligonucleotide for the preceding template (fully complementary)	GAC TAA GAC GAA GAT GTA AAT AAC AAC AAT AAC AAC AAT AAC AAC AAT AAC<br>AAC AAT AAC AAC AAT AAC AAC AAT AAC AAC AAT AAC AAC AAT AAC AAC AAT<br>AAC AAC AAT AAC AAC AAT AAC AAC AAT AAC AAC AAT AAC AAC AAT AAC AAC<br>AAT AAC AAC AAT
5′-tethered, RNA target with let-7a t10–t19 site and a 3′ DNA extension containing 17 Alexa Fluor 647 dyes	Bio-AUU AAA UAU ACA ACC UGA CUA AGA CGA AGA UGU AAA UAA CAA CAA UAA<br>CAA CAA UAA CAA CAA UAA CAA CAA UAA CAA CAA UAA CAA CAA UAA CAA CAA<br>UAA CAA CAA UAA CAA CAA UAA CAA CAA UAA CAA CAA UAA CAA CAA UAA CAA<br>CAA UAA CAA CAA UAA CAA CAA UAA CAA CAA U
5′-tethered, RNA target with let-7a 6mer-A1 site and a 3′ DNA extension containing 17 Alexa Fluor 647 dyes	Bio-AUC UAA AUA AAC CUC AGA CUA AGA CGA AGA UGU AAA UAA CAA CAA UAA<br>CAA CAA UAA CAA CAA UAA CAA CAA UAA CAA CAA UAA CAA CAA UAA CAA CAA<br>UAA CAA CAA UAA CAA CAA UAA CAA CAA UAA CAA CAA UAA CAA CAA UAA CAA<br>CAA UAA CAA CAA UAA CAA CAA UAA CAA CAA U
5′-tethered, RNA target with let-7a 6mer-m8 site and a 3′ DNA extension containing 17 Alexa Fluor 647 dyes	Bio-AUU UCC AAG CCU ACC UGA CUA AGA CGA AGA UGU AAA UAA CAA CAA UAA<br>CAA CAA UAA CAA CAA UAA CAA CAA UAA CAA CAA UAA CAA CAA UAA CAA CAA<br>UAA CAA CAA UAA CAA CAA UAA CAA CAA UAA CAA CAA UAA CAA CAA UAA CAA<br>CAA UAA CAA CAA UAA CAA CAA UAA CAA CAA U
**RBNS**	**Sequence**<br>RNA, DNA
RBNS RNA pool	GAG UUC UAC AGU CCG ACG AUC NNN NNN NNN NNN NNN NNN NNU GGA AUU CUC<br>GGG UGC CAA
RT primer	CCT TGG CAC CCG AGA ATT CCA
PCR Forward primer	AAT GAT ACG GCG ACC ACC GAG ATC TAC ACG TTC AGA GTT CTA CAG TCC GAC<br>GAT C
Multiplexing PCR Reverse Primer (XXXXXX represents barcode)	CAA GCA GAA GAC GGC ATA CGA GAT XXX XXX GTG ACT GGA GTT CCT TGG CAC<br>CCG AGA ATT CCA

Table S3. Dissociation constants for 3′-only sites, Related to Figure 6

Values are reported in pM. Intervals correspond to 95% confidence intervals on the median.

miRNA	6mer	10mer-m10.19	10mer-m11.20	10mer-m12.21	10mer-m13.22	10mer-m14.23
let-7a^{mmu}	348 [337,387]	630 [469, 830]	158 [145, 165]	323 [297, 335]	NA	NA
let-7a^{hsa}	82.5 [81.7, 83.6]	689 [677, 698]	512 [507, 519]	1414 [1379, 1475]	2733 [2699, 2771]	NA
miR-124	475 [472, 478]	110 [89, 141]	226 [221, 233]	635 [624, 643]	1390 [1381, 1401]	NA
miR-155	154 [152, 155]	591 [586, 597]	250 [248, 252]	94.2 [92.7, 95.2]	36.7 [36.3, 37.0]	139 [138, 141]
miR-1	62.1 [62.0, 62.2]	2441 [2402, 2480]	1199 [1181, 1218]	1298 [1281, 1327]	2334 [2282, 2395]	NA
lsy-6	450 [447, 451]	241 [239, 243]	386 [380, 393]	776 [763, 784]	710 [704, 717]	NA
miR-7	140 [140, 140]	2128 [2090, 2175]	1420 [1394, 1439]	1397 [1373, 1414]	1745 [1711, 1771]	2301 [2262, 2338]
miR-34b	167 [720, 733]	3211 [3160, 3271]	726 [720, 733]	998 [986, 1010]	1058 [1049, 1067]	1712 [1702, 1726]
miR-449a	62.4 [57.5, 62.8]	448 [443, 451]	869 [852, 889]	679 [670, 687]	936 [921, 962]	NA

Table S4. Robustness of fitted k_{on} and k_{off} parameters, Related to Figure 7

Maximum likelihood estimation was performed on sub-datasets where each sample was removed one by one. Pearson's *r* was calculated for each of the fifty-five pairwise combinations.

	0 s	15 s	30 s	1 min	2.5 min	5 min	10 min	15 min	30 min	1 h
**15 s**	1.00									
**30 s**	0.993	0.993								
**1 min**	0.999	0.999	0.995							
**2.5 min**	1.00	1.00	0.993	0.999						
**5 min**	1.00	1.00	0.993	0.999	1.00					
**10 min**	1.00	1.00	0.994	0.999	1.00	1.00				
**15 min**	1.00	1.00	0.993	0.999	1.00	1.00	1.00			
**30 min**	1.00	1.00	0.993	0.999	1.00	1.00	1.00	1.00		
**1 h**	1.00	1.00	0.993	0.999	1.00	1.00	1.00	1.00	1.00	
**2 h**	1.00	1.00	0.993	0.999	1.00	1.00	1.00	1.00	1.00	1.00

SUPPLEMENTAL FIGURES AND LEGENDS

Figure S1. RBNS biases relative to RNA pool design, Related to Figure 1

(A) Frequency of the four nucleotides at each position within the randomized region of RNA molecules. Adenine (orange), uridine (magenta), cytidine (cyan), and guanosine (blue).

(**B**) Distribution of frequencies of 10-nt long motifs within RNA molecules. f_{expected} is the theoretical frequency of a given 10-mer, $1/4^{10} = 9.54 \times 10^{-7}$.

(C) Canonical sites have contiguous pairing (vertical bar) to the miRNA seed (bold), and some include an additional match to miRNA nucleotide 8 or an A opposite miRNA nucleotide g1. B indicates not A, i.e., C, G, or U.

(D) Enrichment of canonical sites at each position (counted from the 5′ end) within the randomized region of RNA molecules. We note that cytosine—the last nucleotide of the 5′ constant region and the t8 nucleotide of let-7a, miR-34b and miR-449a canonical binding sites—is adjacent to a favorable dinucleotide context (McGeary et al., 2019), yielding ~2–3-fold greater enrichment of sites starting at this position.

(E) Rastergram summaries of traces of individual target molecules, each in a single row and sorted according to their arrival time. Blue intervals indicate binding events. Above: representative fluorescence intensity time traces for miRISC (turquoise) binding RNA targets (magenta) with different extents of complementarity to the miRNA guide. Gray, background levels of green fluorescence; black,binding events.

(F) Comparison of RBNS results with and without DNA blocking oligonucleotides. Enrichment of miR-449a canonical sites was 2.5–5-fold greater when DNA blockers were used, reflecting a decreased fraction of RNA with no binding site (right).

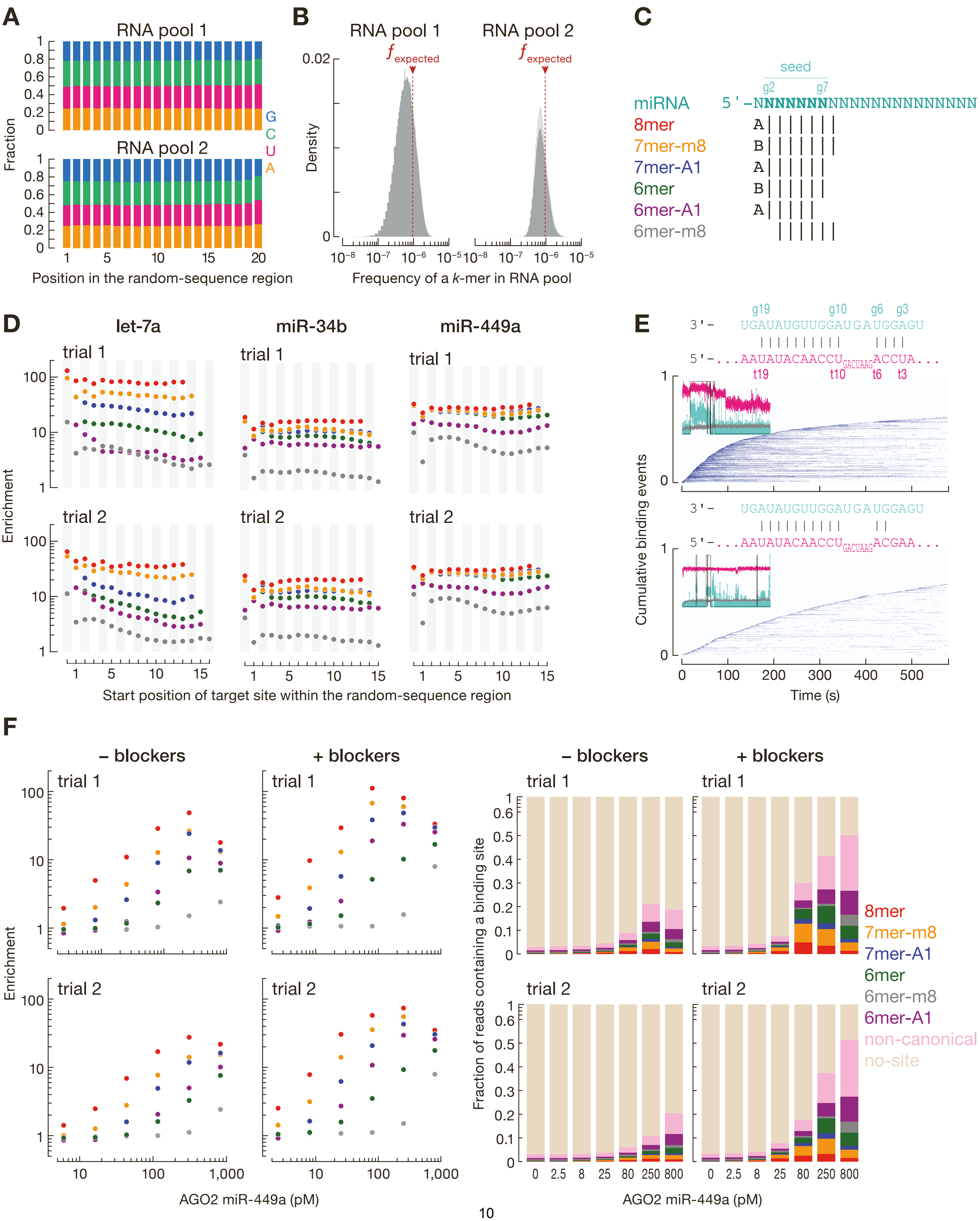
A
RNA pool 1
RNA pool 2
Fraction
G
C
U
A
Position in the random-sequence region
B
RNA pool 1
RNA pool 2
f expected
Density
Frequency of a k-mer in RNA pool
C
seed
g2
g7
miRNA
5'-NNNNNNNNNNNNNNNNNNNNN
8mer
7mer-m8
7mer-A1
6mer
6mer-A1
6mer-m8
D
let-7a
miR-34b
miR-449a
trial 1
trial 2
Enrichment
Start position of target site within the random-sequence region
E
g19 g10 g6 g3
3'- UGAUAUGUUGGAUGAUGGAGU
5'-...AAUAUACAACCU GACUAAG ACCUA...
t19 t10 t6 t3
3'- UGAUAUGUUGGAUGAUGGAGU
5'-...AAUAUACAACCU GACUAAG ACGAA...
Cumulative binding events
Time (s)
F
– blockers
+ blockers
trial 1
trial 2
Enrichment
Fraction of reads containing a binding site
8mer
7mer-m8
7mer-A1
6mer
6mer-m8
6mer-A1
non-canonical
no-site
AGO2 miR-449a (pM)

Figure S2. RBNS biases relative to technical procedures, Related to Figure 3

(A) Overview of RNA Bind-n-Seq using pull-down on magnetic beads coupled to anti-FLAG antibody.

(B) Enrichment profile of canonical let-7a sites observed at each of the five miRISC concentrations in RBNS including pull-down step, determined as ratio of site frequency in protein sample over site frequency in the RNA pool.

(C) Comparison of binding sites identified by de novo discovery in RBNS including double-filter or pull-down step. Minus sign indicates sites that were not identified by de novo site analysis.

(D) Comparison of dissociation constants estimated from RBNS including double-filter or pull-down step. Error bars indicate 95% confidence intervals on the median. Indicated are the site types showing >2-fold difference. The solid orange and red lines indicate 2-fold and 10-fold difference, respectively. Dashed diagonal lines show $y = x$.

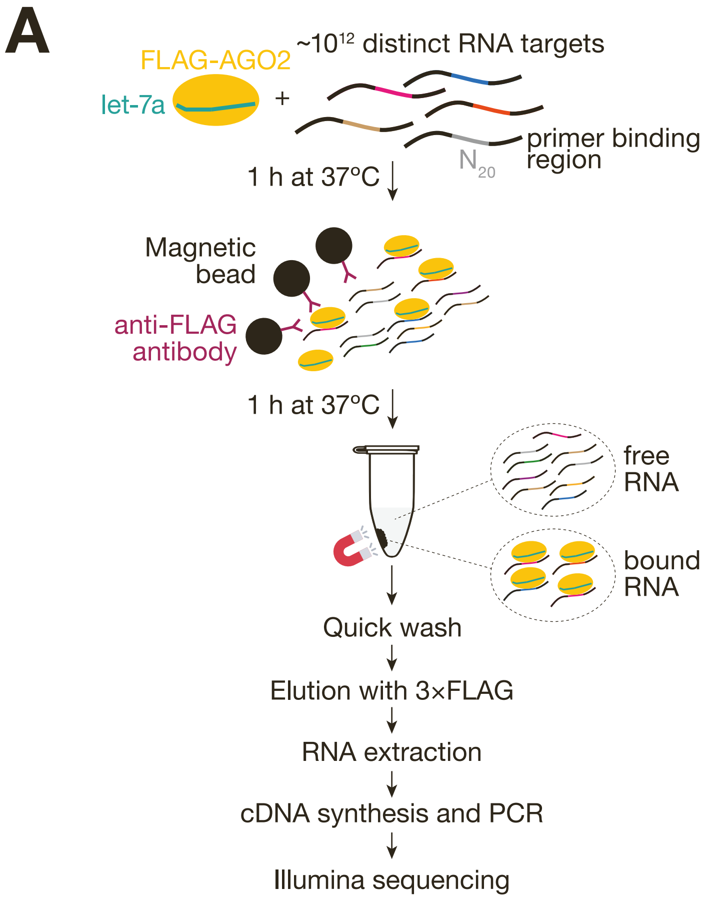

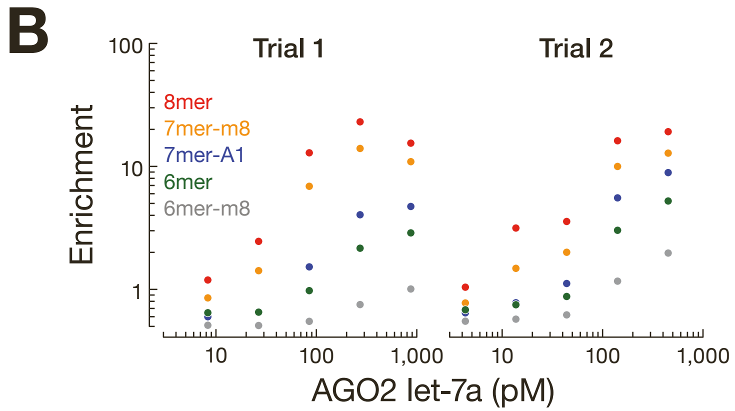

C

		Nitrocellulose filter trial # 1	Nitrocellulose filter trial # 2	FLAG beads trial # 1	FLAG beads trial # 2
canonical	8mer	•	•	•	•
	7mer-m8	•	•	•	•
	7mer-A1	•	•	•	•
	6mer	•	•	•	•
	6mer-m8	•	•	•	•
seed-matched	8mer-w8	•	•	•	•
	7mer-w8	•	•	•	•
	6mer-A1	•	•	•	•
	5mer-m2.6	•	•	•	•
	8mer-w6	•	•	•	•
	7mer-w6	•	•	•	–
	8mer-w5	–	•	•	–
	7mer-w5	–	•	•	–
	8mer-w4	–	–	–	•
	5mer-A1	•	•	•	•
	8mer-b5.6A	•	•	•	•
3'-only	10mer-m11.20	•	•	–	–
	10mer-m12.21	•	•	–	–

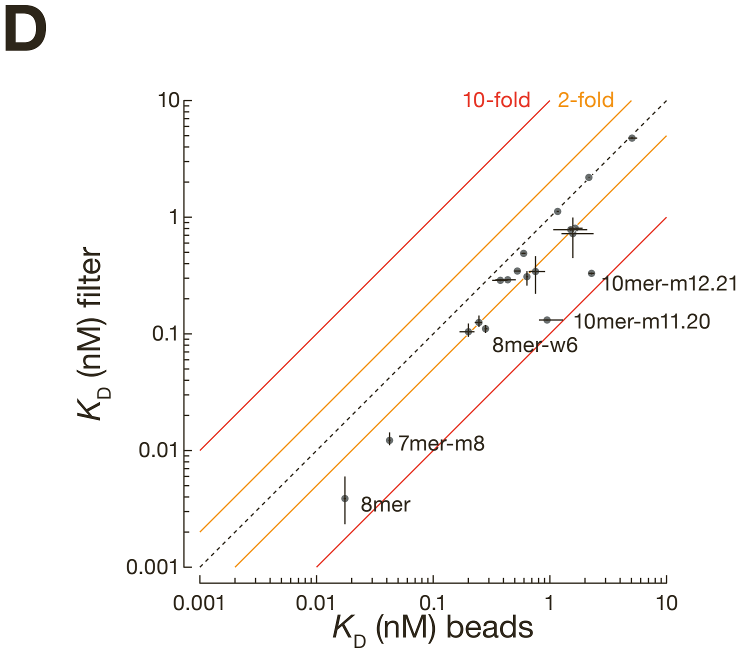

Figure S3. RBNS biases relative to protein concentration and significance criteria for de novo site discovery, Related to Figure 3

(A) Enrichment of reads containing canonical let-7a sites in no-RISC and guide-only binding reactions. Dashed line indicates $y = x$.

(B) Comparison of binding sites identified by de novo discovery when using binding reactions of different miRISC concentrations.

(C) Illustration of de novo site discovery algorithm, using enrichment threshold instead of *Z*-score threshold presented in Figure 3C.

(D) Comparison of binding sites identified by de novo discovery in RBNS including double-filter or pull-down step when using enrichment threshold.

(E) Distribution of *Z*-scores in RBNS data including double-filter or pull-down step and choice of the *Z*-score for significance.

A

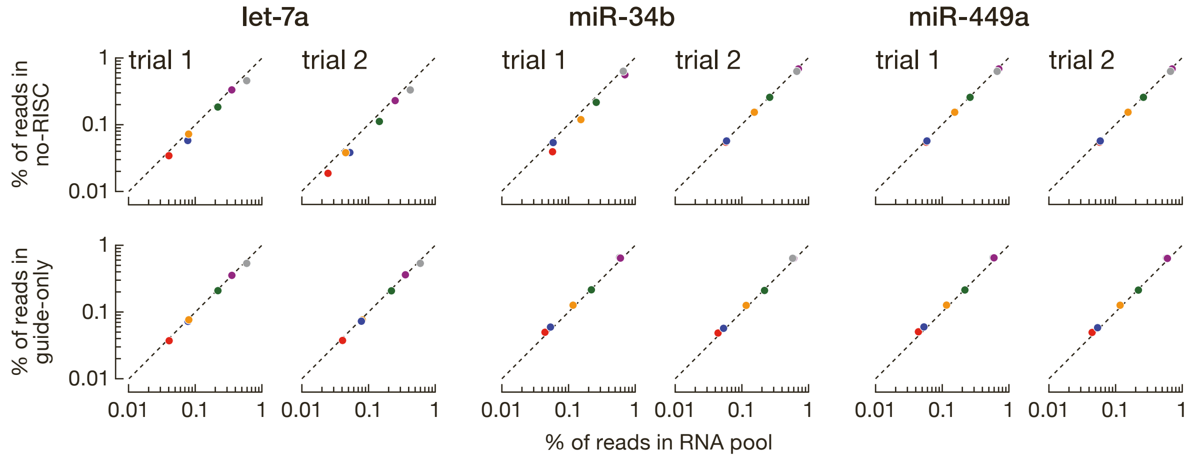

B

	8 pM	25 pM	80 pM	253 pM	800 pM
8mer	•	•	•	•	•
7mer-m8	•	•	•	•	•
7mer-A1	–	•	•	•	•
6mer	–	–	•	•	•
6mer-A1	–	–	•	•	•
6mer-m8	–	–	•	•	•
10mer-m11.20	–	–	•	•	•
8mer-w6	–	–	•	•	•
8mer-w8	–	–	–	•	•
7mer-w8	–	–	–	–	•
7mer-w6	–	–	–	–	•
10mer-m12.21	–	–	–	–	•
8mer-b5.6A	–	–	–	–	•
5mer-m2.6	–	–	–	–	•
5mer-A1	–	–	–	–	•

C

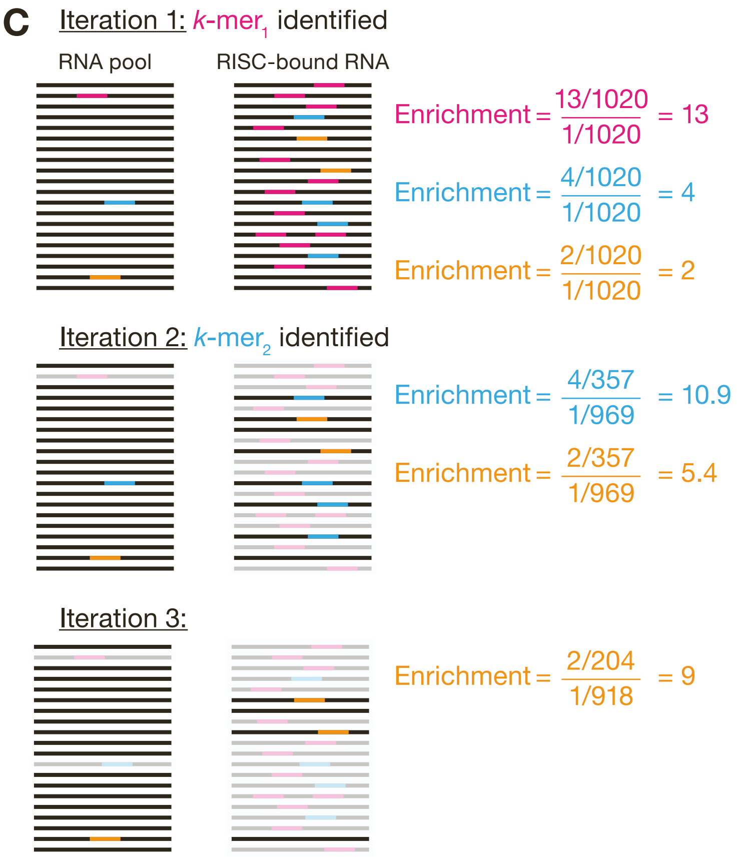

D

		nitrocellulose filter		FLAG beads	
	trial #	1	2	1	2
canonical	8mer	•	•	•	•
	7mer-m8	•	•	•	•
	7mer-A1	•	•	•	•
	6mer	•	•	–	–
	6mer-m8	•	•	–	–
seed-matched	8mer-w8	•	•	–	•
	7mer-w8	•	•	–	–
	6mer-A1	•	•	–	–
	5mer-m2.6	•	•	–	–
	8mer-w6	•	•	•	–
	7mer-w6	•	•	–	–
	8mer-w4	•	•	–	–
	8mer-w5	•	•	–	–
	7mer-w5	•	•	–	–
	5mer-A1	•	•	–	–
	8mer-b5.6A	•	•	–	–
3'-only	10mer-m11.20	•	•	–	–
	10mer-m12.21	•	•	–	–

E

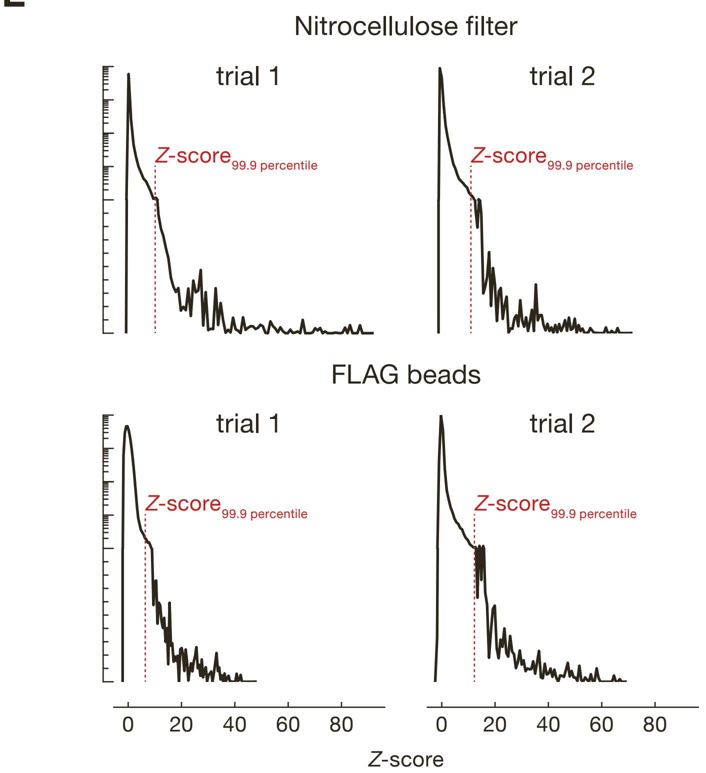

Figure S4. Estimation of K_D values by RBNS is robust and reproducible, Related to Figure 4

(A–B) Testing K_D estimation with simulated data. RBNS data were modeled by simulating miRISC binding to RNA pool containing four binding sites and no-site molecules. Stock concentration of miRISC was equal to 2.1 nM (dataset 1) or 8.1 nM (datasets 2 and 3). Each simulated RBNS experiment contained six binding reactions—five logarithmically spaced miRISC dilutions and a no-RISC binding reaction—and RNA pool at 100 nM. Background was set to 0.1 nM and $K_{D,\ nosite}$ was set to 5 nM. **(A)** Convergence of a representative MLE fit. **(B)** Each fitting optimization was performed 1,000 times using a different set of initial parameter values. Intervals correspond to 95% confidence intervals on the median.

(C) To estimate the robustness of fitted parameters, maximum likelihood estimation was performed on sub-datasets where each sample was removed one by one. Pearson's *r* was calculated for each of the fifteen pairwise possibilities.

(D) K_D values for t2–t8, t2–t7 and t2–t6 let-7a targets with different t1 nucleotides. Adenine (orange), uridine (magenta), cytidine (cyan), and guanosine (blue).

(E, F) Measuring k_{on}, k_{off} and K_D values of 6mer, 6mer-A1 and 6mer-m8 let-7a sites by CoSMoS. **(F)** Representative fluorescence intensity time traces of miRISC (turquoise) binding RNA target (magenta) with different extents of complementarity to the miRNA guide. Gray indicates background levels of green fluorescence, whereas the black line denotes binding events. Cumulative fraction of let-7a-guided AGO2 molecules binding for the first time to a single RNA target and dwell time distribution of these binding events. Data points are plotted in black, and the curve in blue shows the rate of binding or departure after correcting for non-specific association of miRISC with the glass surface. k_{on} and k_{off} values were derived from data collected from >900 individual RNA target molecules; standard error from bootstrapping is reported.

A

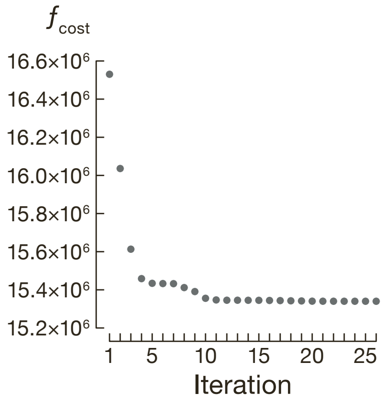

B

		$[RISC]_{stock}$	$K_{D,nosite}$	background
Set 1	Ground truth	2.1 nM	5 nM	0.1 nM
	Estimated	3.7 nM [3.4, 3.9]	2.3 nM [2.2, 2.4]	0.09 nM [0.09, 0.10]
Set 2	Ground truth	8.1 nM	5 nM	0.1 nM
	Estimated	8.7 nM [8.1, 9.3]	1.5 nM [1.4, 1.5]	0.03 nM [0.03, 0.03]
Set 3	Ground truth	8.1 nM	5 nM	0.1 nM
	Estimated	25 nM [25, 26]	3.7 nM [3.7, 3.8]	0.05 nM [0.05, 0.06]

C

	0 pM	8 pM	25 pM	80 pM	250 pM
**8 pM**	0.999				
**25 pM**	1.00	0.999			
**80 pM**	1.00	1.00	1.00		
**250 pM**	1.00	0.999	1.00	1.00	
**800 pM**	0.979	0.969	0.978	0.974	0.979

E

Binding site	k_{on} (M^{-1} s^{-1})	k_{off} (s^{-1})	K_D (nM)
6mer	1.00 ± 0.02 × 10^8	0.040 ± 0.001	0.40 ± 0.02
6mer-m8	1.40 ± 0.02 × 10^8	0.290 ± 0.002	2.1 ± 0.04
6mer-A1	1.50 ± 0.01 × 10^8	0.330 ± 0.002	2.2 ± 0.03

D

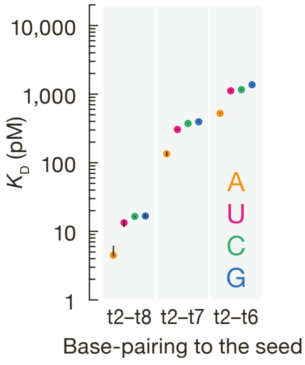

F

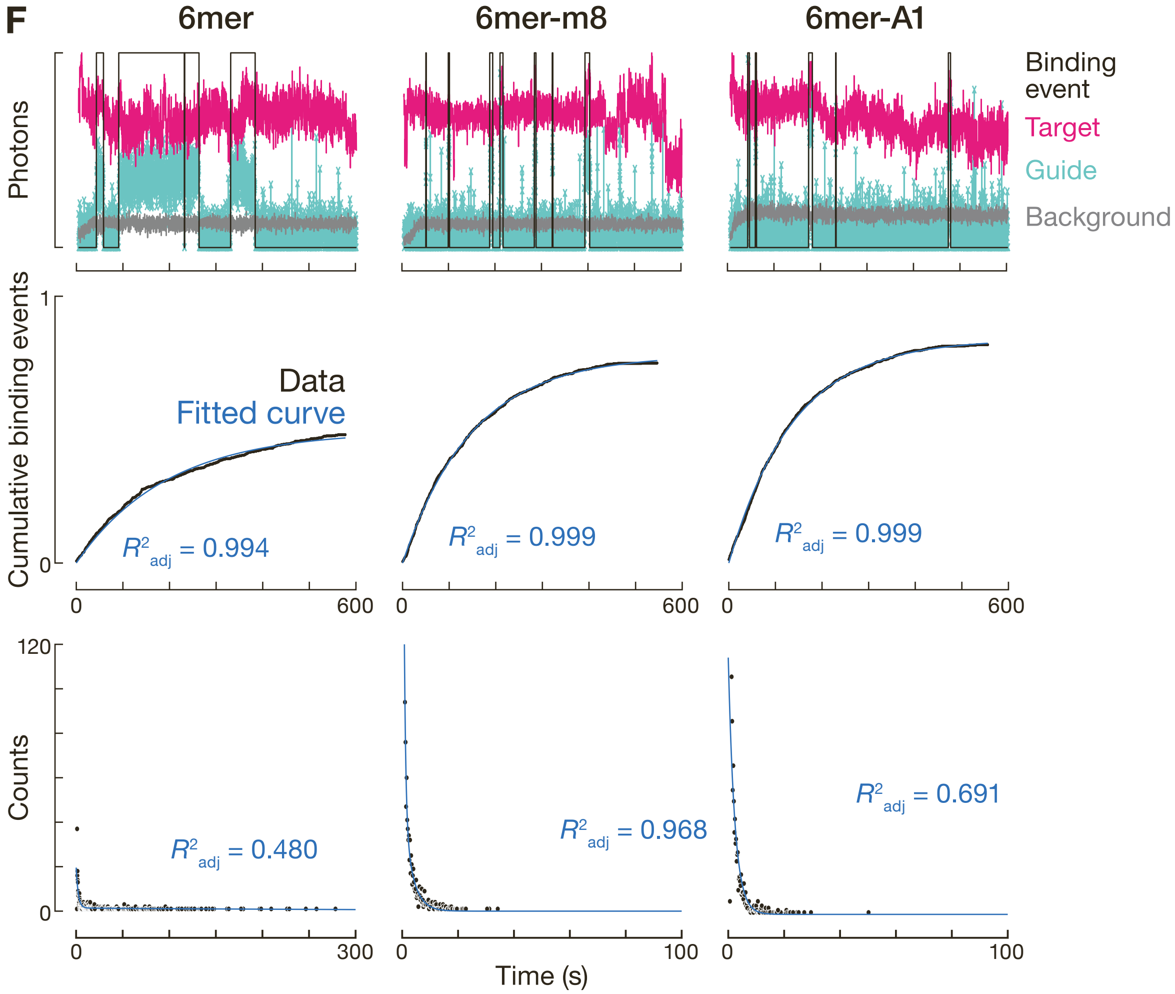

Figure S5. Sequence-specific differences in miRNA affinities, Related to Figure 6

(A) Dissociation constants fitted for AGO2 miR-1, lsy-6 and miR-155 RBNS from a prior study (McGeary et al., 2019). On the left is pairing of enriched sites identified by de novo site discovery. Enrichment profile of canonical sites is shown on the right. **(B)** The relationship between the observed and predicted K_D values for t2–t8, t2–t7, t2–t6 and t3–t8 binding sites of eight miRNAs. While affinity increased with increased predicted pairing stability, the correlation between measured affinity and affinity predicted by nearest-neighbor free energy was significant only for 7mer-m8 binding sites. Nearest neighbor analysis predicts that miR-449, the most stably pairing miRNA in our study, should bind much more tightly to its targets than lsy-6: >17,500-fold for a 6mer, >8,300-fold for a 5mer-m2.6, and >4,200-fold for 6mer-m8 sites. Yet when bound to AGO2, the actual ratios of the affinities of the two miRNAs are quite small: 6.9-fold for a 6mer, 2.6-fold for a 5mer-m2.6, and 0.97-fold for 6mer-m8 sites. In other words, AGO2 reduces the dynamic range of intrinsic differences in seed-pairing stabilities by several orders of magnitude.

Error bars indicate 95% confidence intervals of the median.

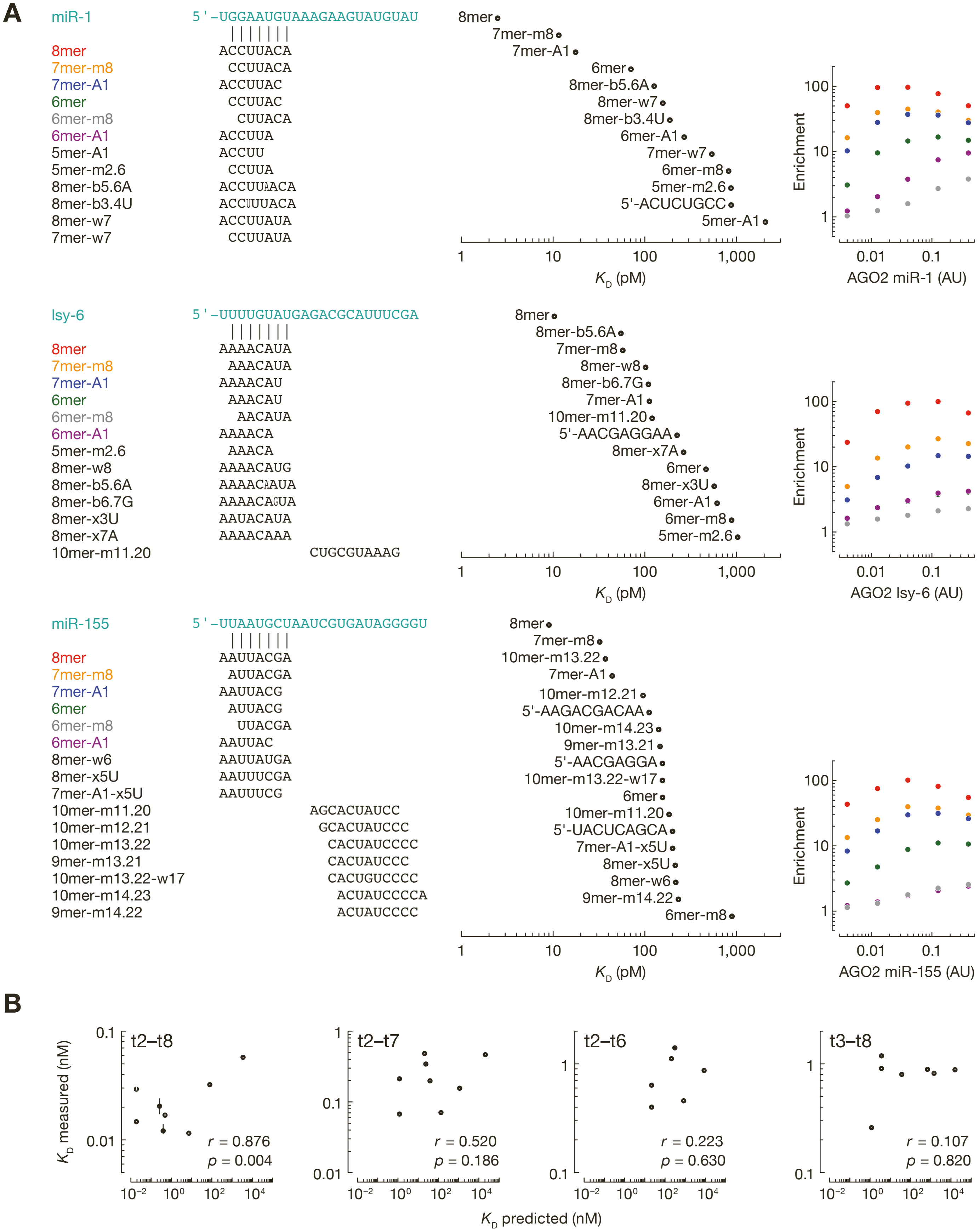
A
miR-1
5'-UGGAAUGUAAAGAAGUAUGUAU
8mer ACCUUACA
7mer-m8 CCUUACA
7mer-A1 ACCUUAC
6mer CCUUAC
6mer-m8 CUUACA
6mer-A1 ACCUUA
5mer-A1 ACCUU
5mer-m2.6 CCUUA
8mer-b5.6A ACCUUAACA
8mer-b3.4U ACCUUUACA
8mer-w7 ACCUUAUA
7mer-w7 CCUUAUA
8mer
7mer-m8
7mer-A1
6mer
8mer-b5.6A
8mer-w7
8mer-b3.4U
6mer-A1
7mer-w7
6mer-m8
5mer-m2.6
5'-ACUCUGCC
5mer-A1
1
10
100
1,000
K_D (pM)
Enrichment
100
10
1
0.01
0.1
AGO2 miR-1 (AU)
lsy-6
5'-UUUUGUAUGAGACGCAUUUCGA
8mer AAAACAUA
7mer-m8 AAACAUA
7mer-A1 AAAACAU
6mer AAACAU
6mer-m8 AACAUA
6mer-A1 AAAACA
5mer-m2.6 AAACA
8mer-w8 AAAACAUG
8mer-b5.6A AAAACAAUA
8mer-b6.7G AAAACAGUA
8mer-x3U AAUACAUA
8mer-x7A AAAACAAA
10mer-m11.20 CUGCGUAAAG
8mer
8mer-b5.6A
7mer-m8
8mer-w8
8mer-b6.7G
7mer-A1
10mer-m11.20
5'-AACGAGGAA
8mer-x7A
6mer
8mer-x3U
6mer-A1
6mer-m8
5mer-m2.6
1
10
100
1,000
K_D (pM)
Enrichment
100
10
1
0.01
0.1
AGO2 lsy-6 (AU)
miR-155
5'-UUAAUGCUAAUCGUGAUAGGGGU
8mer AAUUACGA
7mer-m8 AUUACGA
7mer-A1 AAUUACG
6mer AUUACG
6mer-m8 UUACGA
6mer-A1 AAUUAC
8mer-w6 AAUUAUGA
8mer-x5U AAUUUCGA
7mer-A1-x5U AAUUUCG
10mer-m11.20 AGCACUAUCC
10mer-m12.21 GCACUAUCCC
10mer-m13.22 CACUAUCCCC
9mer-m13.21 CACUAUCCC
10mer-m13.22-w17 CACUGUCCCC
10mer-m14.23 ACUAUCCCCA
9mer-m14.22 ACUAUCCCC
8mer
7mer-m8
10mer-m13.22
7mer-A1
10mer-m12.21
5'-AAGACGACAA
10mer-m14.23
9mer-m13.21
5'-AACGAGGA
10mer-m13.22-w17
6mer
10mer-m11.20
5'-UACUCAGCA
7mer-A1-x5U
8mer-x5U
8mer-w6
9mer-m14.22
6mer-m8
1
10
100
1,000
K_D (pM)
Enrichment
100
10
1
0.01
0.1
AGO2 miR-155 (AU)
B
K_D measured (nM)
t2–t8
0.1
0.01
r = 0.876
p = 0.004
t2–t7
1
0.1
0.01
r = 0.520
p = 0.186
t2–t6
1
0.1
r = 0.223
p = 0.630
t3–t8
1
0.1
r = 0.107
p = 0.820
10^{-2} 10^{0} 10^{2} 10^{4}
K_D predicted (nM)

Figure S6. Dissociation constants of enriched sites estimated for miR-7, miR-124, miR-34b, miR-449a and let-7a RBNS datasets, Related to Figure 6

Dissociation constants fitted for AGO2 miR-7 and miR-124 RBNS from a prior study (McGeary et al., 2019) **(A)**, and miR-34b **(B)**, miR-449a **(C)** and let-7a **(D)** from this study. Error bars indicate 95% confidence intervals on the median. **(A, B and D)** Pairing is shown for enriched sites identified by de novo site discovery. **(C)** Pairing of enriched sites as in **(B)**. **(A and B)** Enrichment profile of canonical sites is shown on the right.

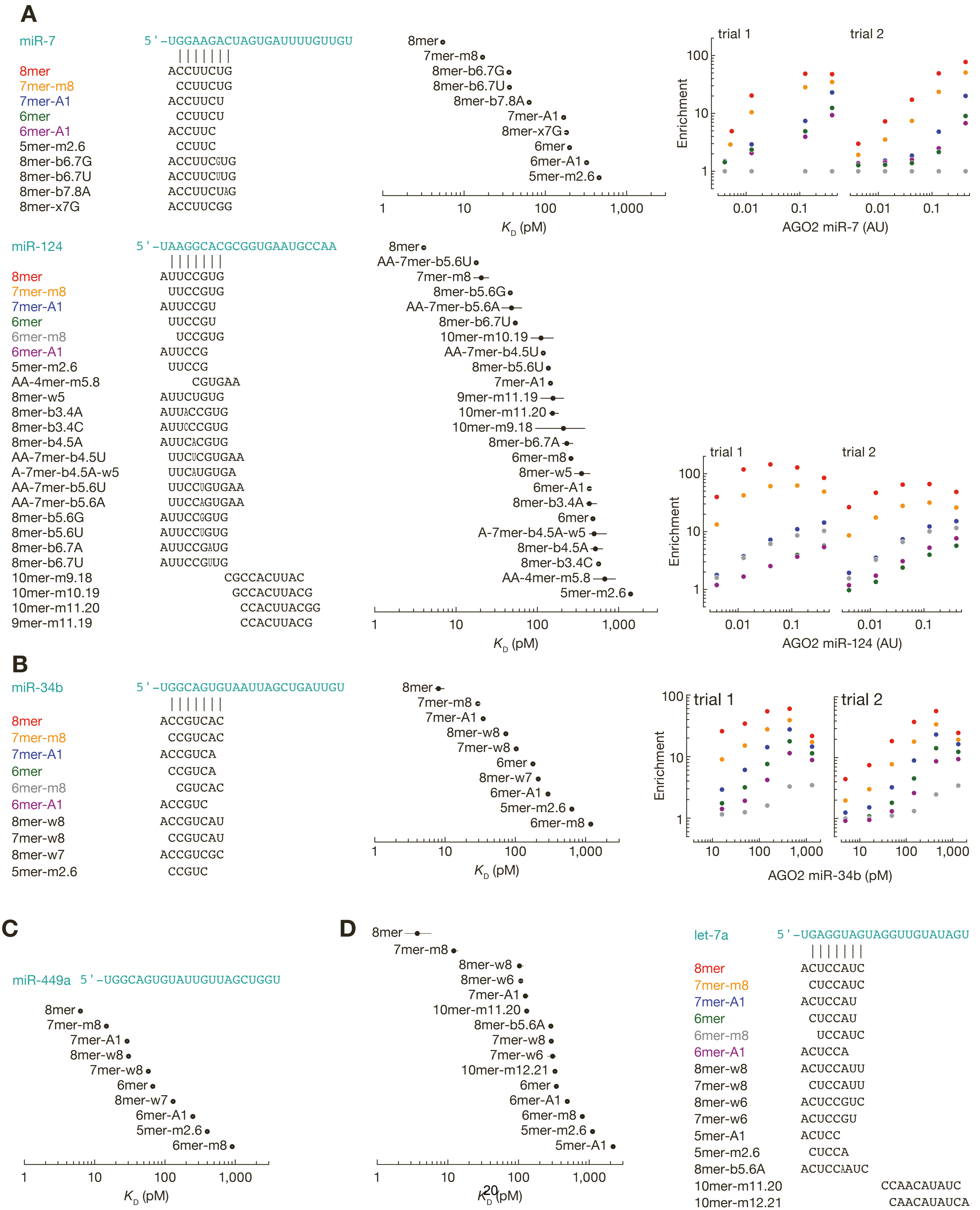
A
miR-7 5'-UGGAAGACUAGUGAUUUUGUUGU
8mer ACCUUCUG
7mer-m8 CCUUCUG
7mer-A1 ACCUUCU
6mer CCUUCU
6mer-A1 ACCUUC
5mer-m2.6 CCUUC
8mer-b6.7G ACCUUCGUG
8mer-b6.7U ACCUUCUUG
8mer-b7.8A ACCUUCUAG
8mer-x7G ACCUUCGG
K_D (pM)
trial 1
trial 2
Enrichment
AGO2 miR-7 (AU)
miR-124 5'-UAAGGCACGCGGUGAAUGCCAA
8mer AUUCCGUG
7mer-m8 UUCCGUG
7mer-A1 AUUCCGU
6mer UUCCGU
6mer-m8 UCCGUG
6mer-A1 AUUCCG
5mer-m2.6 UUCCG
AA-4mer-m5.8 CGUGAA
8mer-w5 AUUCUGUG
8mer-b3.4A AUUACCGUG
8mer-b3.4C AUUCCCGUG
8mer-b4.5A AUUCACGUG
AA-7mer-b4.5U UUCUCGUGAA
A-7mer-b4.5A-w5 UUCAUGUGA
AA-7mer-b5.6U UUCCUGUGAA
AA-7mer-b5.6A UUCCAGUGAA
8mer-b5.6G AUUCCGGUG
8mer-b5.6U AUUCCUGUG
8mer-b6.7A AUUCCGAUG
8mer-b6.7U AUUCCGUUG
10mer-m9.18 CGCCACUUAC
10mer-m10.19 GCCACUUACG
10mer-m11.20 CCACUUACGG
9mer-m11.19 CCACUUACG
K_D (pM)
AGO2 miR-124 (AU)
B
miR-34b 5'-UGGCAGUGUAAUUAGCUGAUUGU
8mer ACCGUCAC
7mer-m8 CCGUCAC
7mer-A1 ACCGUCA
6mer CCGUCA
6mer-m8 CGUCAC
6mer-A1 ACCGUC
8mer-w8 ACCGUCAU
7mer-w8 CCGUCAU
8mer-w7 ACCGUCGC
5mer-m2.6 CCGUC
K_D (pM)
AGO2 miR-34b (pM)
C
miR-449a 5'-UGGCAGUGUAUUGUUAGCUGGU
K_D (pM)
D
K_D (pM)
let-7a 5'-UGAGGUAGUAGGUUGUAUAGU
8mer ACUCCAUC
7mer-m8 CUCCAUC
7mer-A1 ACUCCAU
6mer CUCCAU
6mer-m8 UCCAUC
6mer-A1 ACUCCA
8mer-w8 ACUCCAUU
7mer-w8 CUCCAUU
8mer-w6 ACUCCGUC
7mer-w6 ACUCCGU
5mer-A1 ACUCC
5mer-m2.6 CUCCA
8mer-b5.6A ACUCCAAUC
10mer-m11.20 CCAACAUAUC
10mer-m12.21 CAACAUAUCA

Figure S7. Estimation of k_{on} and k_{off} values by RBNS, Related to Figure 7

(A) Overview of RNA Bind-n-Seq extended to kinetic studies.

(B) Testing k_{on} and k_{off} estimation with simulated data. RBNS data were modeled by simulating miRISC binding to RNA pool containing four binding sites and no-site molecules. Total miRISC concentration was equal to 0.15 nM. Background was set to 0.1 nM. Dataset 1 contains binding sites with similar k_{off} but different k_{on} values. Dataset 2 contains binding sites with similar k_{on} but different k_{off} values. Estimated absolute values of kinetic parameters are not accurately recovered from the ground truth.

(C) k_{on} values for let-7a 7mer-A1, 6mer-A1 and 5mer-A1 sites separated into 4^4 = 256 sites according to the dinucleotide sequences immediately flanking the 5′ and the 3′ ends of the site. To increase statistical power, we classified these 256 sites into 5 bins based on the number of A/U and G/C nucleotides and determined k_{on} for each. Error bars indicate 95% confidence intervals on the median. G and C flanking nucleotides reduced k_{on}, likely by stabilizing RNA secondary structures that occlude the binding site. This effect of flanking dinucleotides on k_{on} was consistent among the three site types, but was greater for shorter, lower-affinity sites: for the 7mer-A1 sites, the most favorable (A/U-rich) contexts were just twice as fast as the most detrimental (G/C-rich), but for the 5mer-A1 sites, the A/U-rich contexts were 29-fold faster than the G/C-rich. Similarly, k_{on} for a 7mer-A1 bracketed by di-adenosines was 7-fold faster than when flanked by di-guanosines; for the 5mer-A1 flanked by di-adenosines was ~93-fold faster than with di-guanosines.

A

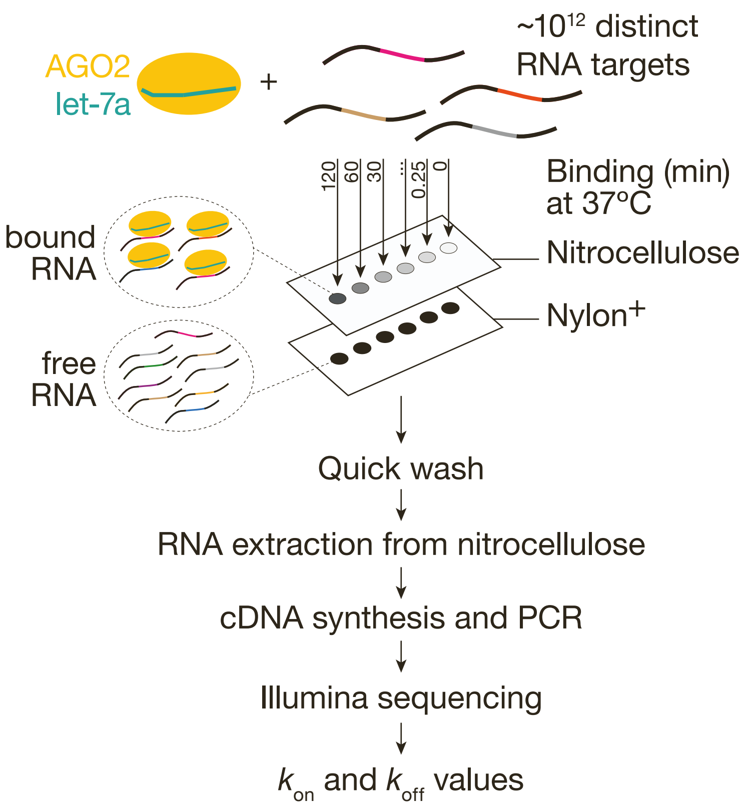

B

		Dataset 1 True	Dataset 1 Estimated	Dataset 2 True	Dataset 2 Estimated
k_{on} (nM^{-1} s^{-1})	Site 1	0.1	5.04	0.1	2.08
	Site 2	0.01	0.543	0.1	2.01
	Site 3	0.001	0.056	0.1	1.95
	Site 4	0.0001	0.007	0.1	5.72
	No-site	0.0001	0.026	0.001	0.010
k_{off} (s^{-1})	Site 1	0.0001	0.001	0.00001	0.0005
	Site 2	0.01	0.285	0.001	0.048
	Site 3	0.01	0.323	0.001	0.495
	Site 4	0.01	0.363	0.1	11.9
	No-site	10	18.1	10	40.5
background (nM)		0.1	0.150	0.1	0.088

C

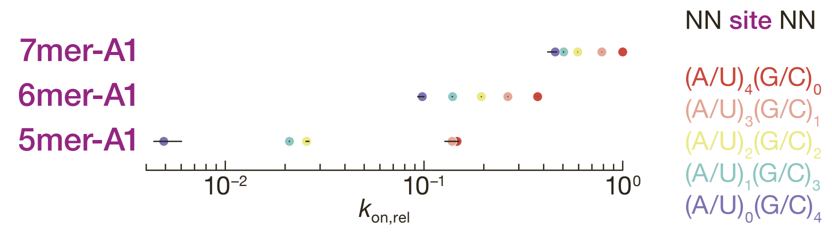

Supplement: Document S2. Article plus supplemental information [file mmc2.pdf]
